# Dissecting the meteorological and genetic factors affecting rice grain quality in Northeast China

**DOI:** 10.1007/s13258-021-01121-z

**Published:** 2021-06-24

**Authors:** Mojun Chen, Zhao Li, Jie Huang, Yongfeng Yan, Tao Wu, Mingdi Bian, Jinsong Zhou, Yongjun Wang, Yanjie Lyv, Guanghui Hu, Yong-Mei Jin, Kai Huang, Liping Guo, Wenzhu Jiang, Xinglin Du

**Affiliations:** 1 Jilin Province Engineering Laboratory of Plant Genetic Improvement, College of Plant Science, Jilin University, No. 5333 Xi’an Road, Changchun, 130062 China; 2 Jilin Academy of Agricultural Sciences, Changchun, 130033 China; 3 Huazhi Rice Bio-Tech Co., Ltd., Changsha, 410125 China; 4 Heilongjiang Academy of Agricultural Sciences, Harbin, 150086 China

**Keywords:** Rice, Grain quality, Meteorological factors, Genetic factors, RNA-seq

## Abstract

**Background:**

The Northeast Plain of China, which is an important region for the production of high grain quality rice in China. However, the grain quality of the rice produced varies across this region, even for the same cultivar.

**Objective:**

In order to explore the meteorological factors that have the greatest influence on quality and the transcriptional level differences between different cultivars and different locations at grain filling stage.

**Methods:**

We grew eight rice cultivars in three locations in Northeast China during two growing seasons (2017 and 2018). We recorded meteorological conditions, including air temperature, air temperature range, and photosynthetically active radiation (PAR) during the grain-filling stage of each cultivar, and analyzed the grain quality of those eight cultivars.

**Results:**

Across all eight cultivars, meteorological factors had a stronger effect on eating quality than genotype, while genotype had a stronger effect on milling quality. Of the three environmental factors assessed, PAR was significantly correlated with the most grain quality traits. Using RNA-sequencing analysis, we identified 573 environment-specific DEGs (Differentially Expressed Genes), and 119 genotype-specific DEGs; 11 DEGs were responsive to genotype × environment interactions. These DEGs were involved in many key metabolic processes.

**Conclusion:**

Our results indicated that interactions among environmental factors, especially PAR, affected rice quality in Northeast China. Further analyses of the DEGs identified herein may provide useful information for future breeding programs aiming to develop high grain quality rice varieties suitable for cultivation across Northeast China.

**Supplementary Information:**

The online version contains supplementary material available at 10.1007/s13258-021-01121-z.

## Introduction

Rice (*Oryza sativa* L.) is staple food for more than half of the world population and is especially important in the developing Asian countries (Global Rice Science Partnership, 2013). Proliferating human populations and improved living standards have concomitantly increased the demand for higher-quality and higher-yield rice (Zeng et al. 2017). However, the higher priority for yield than grain quality has hampered the development of rice varieties with both high yield and high quality (Zhang 2007). In addition, the characteristics defining rice grains perceived as high quality vary among people from different regions, increasing the difficulties and complexities associated with the design of breeding programs to improve rice grain quality (Custodio et al. 2019).

Generally, rice grain quality is judged based on three sets of features: raw-grain properties (including appearance and milling quality), eating and cooking quality (ECQ), and nutritional levels (Kaul 1970). The edible portion of each rice grain primarily consists of starch and protein, the compound species and proportion of which greatly affect the grain quality (Juliano 1990; Ma et al. 2020). As a complex quantitative trait, grain quality is affected by genotype, environment, and genotype × environment interactions (Bao et al. 2004; Kibanda and Luzi-Kihupi 2007; Chen et al. 2012). Using genetic approaches such as quantitative trait locus (QTL) mapping and association mapping, genes associated with grain quality have begun to be characterized (Tian et al. 2009; Liu et al. 2011; Bao 2012; Li et al. 2014; Ren et al. 2016). However, environmental factors, such as light, temperature, water, and soil conditions, also have significant effects on the grain quality (Cheng et al. 2003; Shi et al. 2016; Li et al. 2018). Due to the close interaction between genotypic and environmental effects, the effectiveness of traditional genetic approaches remains limited in dissecting the mechanisms underlying grain quality.

The transcriptome includes all genes expressed in certain tissues or cells under certain conditions, thereby reflecting the sum total of the genotypic, developmental, and environmental responses (Wang et al. 2009). High-throughput transcriptomic analyses based on next-generation sequencing, also known as RNA sequencing (RNA-seq), is an efficient method to identify genes involved in rice grain development (Yi et al. 2013; Sun et al. 2015). Therefore, RNA-seq might help to decipher the genotypic and environmental effects underlying grain quality in rice.

Due to its unique ecology, the Northeast Plain is one of the primary Chinese regions where high grain quality rice (e.g., *japonica* varieties) is produced (Cheng et al. 2002). However, as the area of the Northeast Plain devoted to rice cultivation spans over four million hectares, rice grain quality varies across this area, with Heilongjiang province generally producing the highest-quality rice (Xu et al. 2010). In addition to these geographical variations, the consistent production of high-quality rice is also inhibited by the lack of elite varieties, global climate change, and inappropriate cultivation methods (e.g., the overuse of nitrogen fertilizers). In particular, Jilin province, one of the major rice production bases in Northeast China, produces rice grains of inconsistent and relatively low quality. Current efforts to improve the quality of the rice produced in Northeast China suffer from lack of materials, techniques, and theoretical foundations. Therefore, in this study, we aimed to characterize the effects of genetic and environmental factors on rice grain quality across the various ecological zones of Northeast China. Moreover, the targets we identified could provide new solutions for the breeding of high-quality rice adapted to the various ecological zones of Northeast China.

## Materials and Methods

### Plant materials and growth conditions

**Fig. 1:**
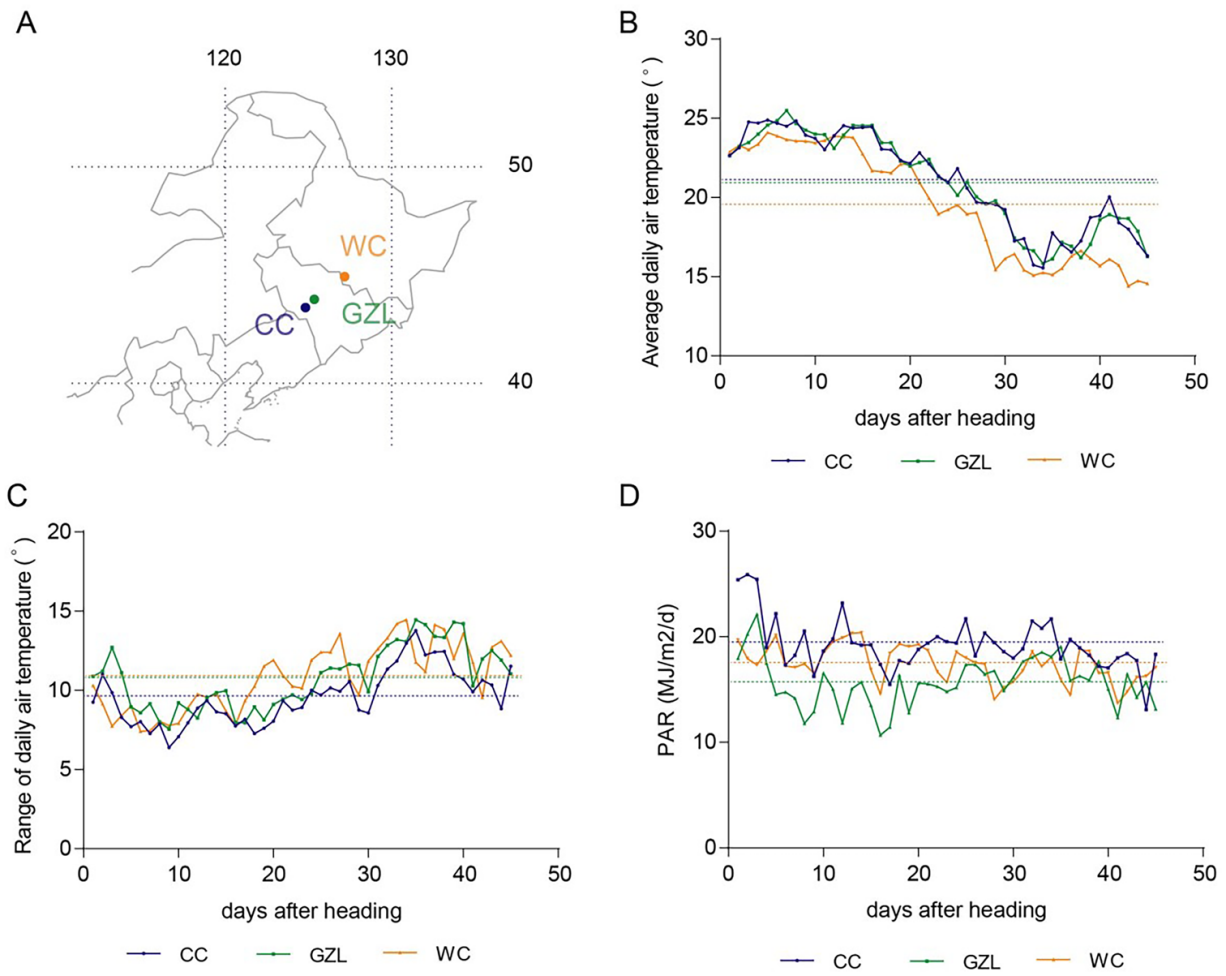
The three locations in Northeast China used in this study. **A** Schematic map of the three locations; numbers at the top and right represent longitude and latitude, respectively. **B**–**D** Environmental factors during the 45 days following heading at each location. **B** Average daily air temperature; **C** daily air temperature range; **D** daily photosynthetically active radiation (PAR). The dotted lines show the average values throughout the grain-filling stage at each location. CC, Changchun city; WC, Wuchang city; GZL, Gongzhuling city

In this study, we used eight rice cultivars that are widely planted in Jilin and Heilongjiang provinces: Jigeng809 (JG809), Jigeng812 (JG812), Jigeng511 (JG511), Daohuaxiang (DHX), Yunlangxiang (YLX), Akihikari (Qiuguang, QG), Jiyugeng (JYG), and Tong35 (T35) (Supplemental Table 1). Field experiments were conducted during the 2017 and 2018 growing seasons in three locations: Changchun city (Jilin Province, China; E125.35°, N43.88°), Gongzhuling city (Jilin Province, China; E124.82°, N43.50°), and Wuchang city (Heilongjiang Province, China; E127.17°, N44.93°) (Fig. 1A). The daily air temperature, range of air temperatures, and photosynthetically active radiation (PAR) in each location were obtained from the China Meteorological Data Service Center (http://data.cma.cn/). Because heading dates differed among cultivars and among locations, we recorded the average daily air temperature, air temperature range, and PAR beginning from the heading date of each plant at each location, and then averaged the values recorded each day for each location. In this way, we calculated the daily mean air temperature, mean air temperature range, and mean PAR during each day of the grain-filling stage of each cultivar at each location.

In 2017 and 2018, all seeds were sown on April 10 in the plates with same matrix. In both years, seedlings were transplanted on May 18 at GZL, May 19 at WC, and May 21 at CC. At each location, seedlings of each cultivar were transplanted into three experimental plots (6 m × 3 m) in randomized blocks, with a plant spacing of 30 cm × 20 cm. Each plot was fertilized with a total of 140 kg nitrogen, 100 kg phosphorous, and 80 kg potassium per hectare. Cultivation methods and field management practices were identical among locations.

For further RNA-seq analyses, we selected three cultivars with different grain quality (DHX, JG809, and T35). To exclude the effects of soil type on grain quality, we transplanted nine seedlings for each cultivars into separate pots filled with soil from Wuchang (three seedlings in a hole and three holes per pot, with 12.5 cm diameter). Fertilizer application and management methods were identical to those used in the field experiments. Three replications were performed.

### Grain quality measurements and data analysis

We harvested 36 plants per cultivar per location (12 plants with uniform performance per plot) for grain quality measurements: brown rice ratio, milled rice ratio, head rice ratio, chalkiness ratio, chalkiness level, protein content, fat content, amylose content, and STA value (indicator for the taste value). Prior to quality measurements, grains were threshed, air dried, and stored at room temperature for 2 months. The brown rice ratio was measured after dehulling using a rice huller (FC2K, Otake, Japan). Then, the brown rice was milled with a rice-polishing machine (VP-32, Yamamoto, Japan), and the milled rice ratio and head rice ratio were calculated. The chalkiness ratio and chalkiness level were measured using a Rice Appearance Quality Analyzer (Beijing Dongfu Jiuheng, China). The protein content, fat content, and amylose content were measured using near-infrared spectroscopy with a Vector 22/N (Bruker Optics, Germany). The STA value was an indicator for the taste value which was measured using a rice taste scoring apparatus (STA1B, Satake, Japan).

Correlation analysis and path analysis, testing the associations between environmental factors and grain quality traits were performed using the 'psych' package (Revelle 2019) in R (version 3.6.1). For these analyses, we used the daily mean air temperature, mean air temperature range, and mean PAR during each day of the grain-filling stage of each seedling at each location (calculated in “Plant materials and growth conditions”). We performed analyses of variance (ANOVAs) and Additive main multiplicative interaction (AMMI) analyses to evaluate the effects of environment, genotype, and the genotype × environment interaction using the R package ‘agricolae’ (version 1.3–3).

### RNA-seq analysis

Both external (i.e., environmental conditions) and internal (i.e., genetic background) factors influence the expression patterns of functional genes. To characterize the transcriptomic responses of rice grains to internal and external factors (environment, genotype, and genotype × environment interaction), we used RNA-seq to generate the transcriptomes of the developing grains at 25 days after heading, which is amid an important stage for grain quality formation (Gong et al. 2013). The developing grains of three panicles per cultivar were collected 25 days after heading from the three cultivars (DHX, JG809, and T35); the lemma and palea were removed from each grain. Total RNA was extracted from the grains using RNAprep Pure kits (TianGen, Beijing). cDNA library construction and next-generation sequencing were performed by Huazhi Rice Bio-Tech (Changsha, China) on an Illumina Hiseq2500 platform (2 × 101 bp). Three biological replicates were performed. The raw reads were filtered using the FASTQ_Quality_Filter tool from the FASTX-Toolkit (http://hannonlab.cshl. edu/fastx_toolkit). Clean sequence reads were submitted to the GenBank GEO database (accession number PRJNA610676). Next, the clean reads were mapped to the rice reference genome (MSU, Rice Genome Annotation Release 7) using Hisat2. Gene expression profiles were analyzed using Stringtie, and transcript abundance was measured using the Fragment Per Kilobase of transcript sequence per Millions base pairs Sequenced (FPKM) method. Differentially expressed genes were identified using the DESeq2 package in R as follows: for each cultivar, we identified the genes differentially expressed between pairs of locations; conversely, for each location, we identified the genes differentially expressed between pairs of cultivars.

To better understand the functional importance of the identified environment- and/or genotype-responsive DEGs, we performed gene ontology (GO) analysis using agriGO (http://bioinfo.cau.edu.cn/agriGO/index.php). Significantly enriched GO terms were identified using Singular Enrichment Analysis (SEA) against the rice reference genome (MSU Rice Genome Annotation Release 7). To avoid the identification of redundant GO terms, the GO terms identified as enriched in the environment-responsive DEGs, as well as the genotype-responsive DEGs, were further analyzed using REVIGO (http://revigo.irb.hr/).

### qRT-PCR analysis

cDNAs were synthesized using One-step gDNA Removal and cDNA Synthesis Super Mix (TRansScript, Beijing). qRT-PCR was performed with SYBR Green mix (TOYOBO, Japan) using Chromo4 real-time PCR detection system (BIO-RAD, CFX96). The rice *Actin1* gene was used as the internal control. The primers used for qRT-PCR were given in Suppelmental Table 9.

## Results

### Environmental conditions at the three locations

The average air temperature dropped gradually during grain filling at all the three locations, with the lowest average air temperatures recorded in Wuchang (Fig. 1b). The range of daily air temperatures tended to increase at all three locations during grain filling, with the smallest daily range recoded in Changchun (Fig. 1c). Daily PAR was more fluctuating among the three locations during grain filling, with the greatest average daily PAR recorded at Changchun, followed by Wuchang and Gongzhuling (Fig. 1d).

### Grain quality among locations

**Fig. 2:**
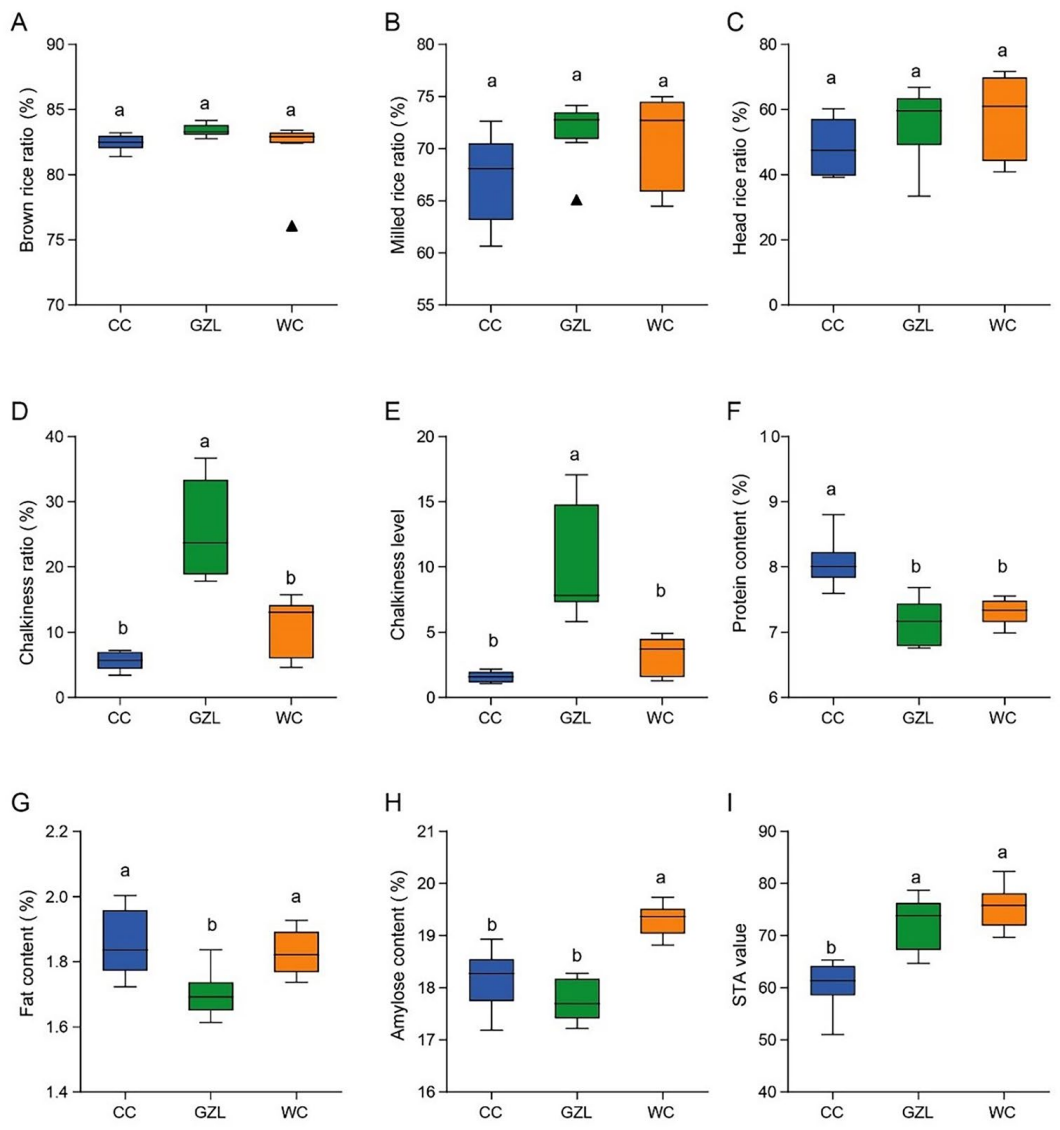
Average grain quality parameters across the eight rice cultivars at each location. **A** brown rice ratio; **B**, milled rice ratio; **C**, head rice ratio; **D**, chalkiness ratio; **E**, chalkiness level; **F** protein content; **G**, fat content; **H**, amylose contents; **I**, STA value (taste score). CC, Changchun city; WC, Wuchang city; GZL, Gongzhuling city. Different letters above boxes indicate significant differences (*P* < 0.05, Duncan’s new multiple range method, values = means ± SD, *n* = 3 biological replicates)

As the grain-quality data collected in 2017 and 2018 were highly correlated and had similar relationships with environment factors, we used the data collected in 2017 for all subsequent analysis (Supplemental Tables 2 and 3). Factors associated with milling quality (brown rice ratio, milled rice ratio, and head rice ratio) did not differ significantly among locations (Fig. 2a–c). Chalkiness ratio and level were significantly higher in the grains grown in Gongzhuling as compared to those grown in Wuchang or Changchun (Fig. 2d, e). Nutritional quality markers varied among locations. Protein content was significantly higher in the grains grown in Changchun than in those grown in Gongzhuling or Wuchang (Fig. 2f). Fat content was significantly lower in the grains grown in Gongzhuling than in those grown in Changchun or Wuchang (Fig. 2g). Amylose content was significant higher in the grains grown in Wuchang than in those grown in Changchun or Gongzhuling (Fig. 2h). STA value was significantly lower in the grains grown in Changchun than in those grown in Gongzhuling or Wuchang (Fig. 2i).

In addition, the yield performance of the eight varieties was analyzed (Supplemental Fig. 1). No significant changes were found among three locations for cultivars including JG511, JG809, JYG, and T35. As for the other cultivars: DHX showed highest yield in CC; JG812 and YLX showed highest yield in GZL; QG showed lowest yield in CC.

### Relationships between environmental factors and rice quality

**Fig. 3:**
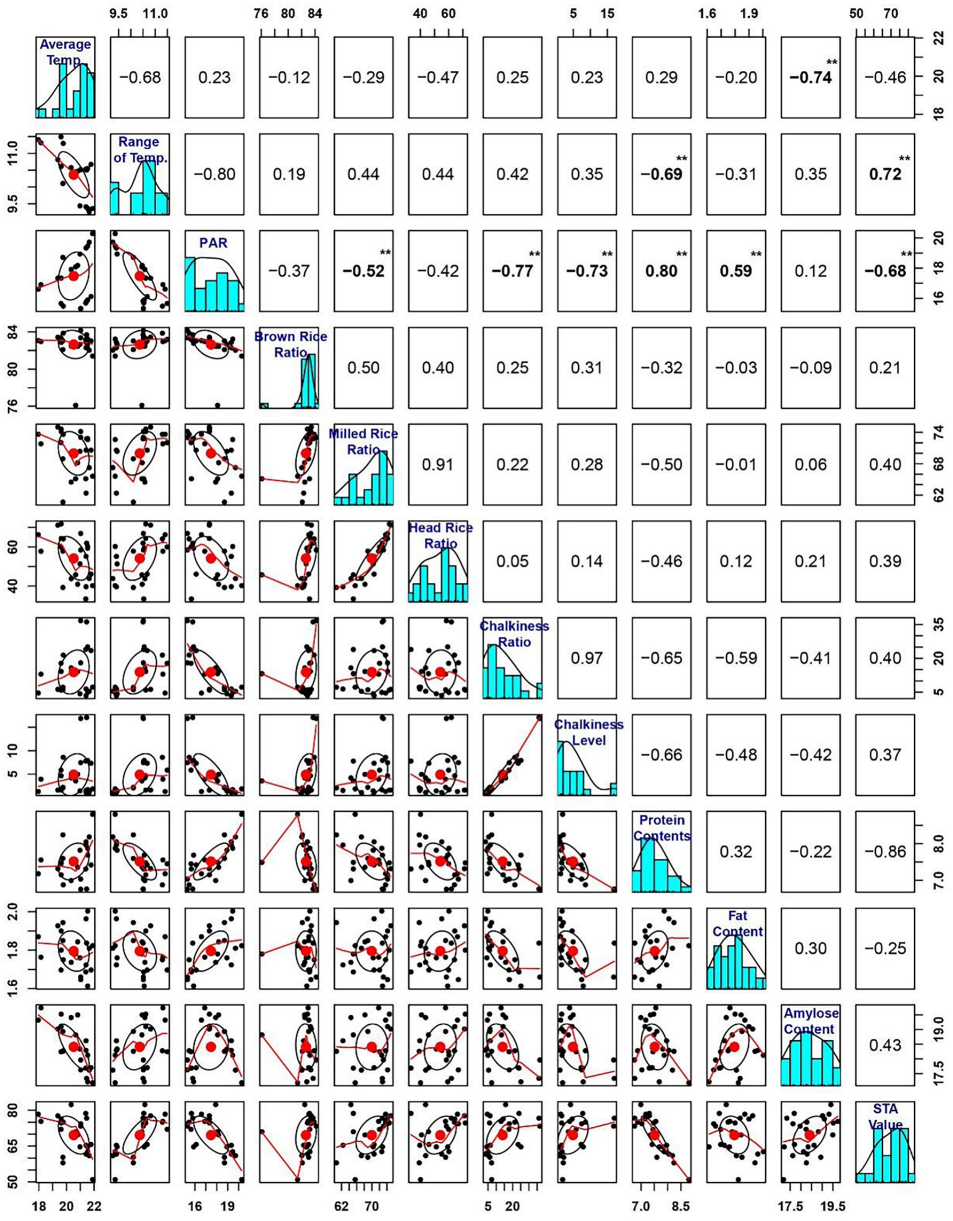
Pairwise distributions and correlations between environmental factors and grain quality parameters. Frequency distributions for each trait or index are illustrated as histograms along the central diagonal. The numbers in cells above diagonal are the correlation coefficients; significant correlations are displayed in bold (*P < 0.05, **P < 0.01). Scatter plots of the correlations between pairs of traits are shown in cells below the diagonal; the red lines show the correlation trends and the red circles indicate the medians

Of the three environmental factors studied here, PAR was significantly correlated with more grain quality traits than average air temperature or average air temperature range (Fig. 3). PAR was significantly negatively correlated with milled rice ratio, chalkiness ratio, chalkiness level, and STA value, but significantly positively correlated with fat content and protein content (Fig. 3). Temperature range was significantly negatively correlated with protein content, but significantly positively correlated with STA value (Fig. 3). Finally, temperature was significantly negatively correlated with amylose content (Fig. 3). Interestingly, the correlation was at a relatively low level between environmental factors and milling quality (i.e., brown rice ratio and head rice ratio).

Our path analysis of the interactions between environmental factors and rice grain quality showed that the indirect path coefficients from temperature range through PAR were − 0.649 for protein content and 0.393 for STA value. Therefore, the interaction between temperature range and PAR affected protein content and STA value.

### Effects of genotype, environment, and the genotype × environment interaction

Genotype most strongly affected the factors associated with milling quality (brown rice ratio, milled rice ratio, and head rice ratio), while environmental conditions had a greater effect on chalkiness ratio, chalkiness level, protein content, fat content, amylose content, and STA value (all effects were significant, *P* < 0.05; Table 1). The genotype × environment interaction also significantly affected all tested grain quality traits except brown rice ratio, with the strength of the effects ranging from 4.62% for STA value to 23.53% for chalkiness level (Table 1).

**Table 1:** Analyses of variance and AMMI models for the grain quality traits of eight cultivars in three locations

Source	D.F	Brown Rice Ratio		Milled Rice Ratio		Head Rice Ratio		Chalkiness Ratio		Chalkiness Level		Protein Content		Fat Content		Amylose Content		STA value	
		MS^a^	SS%^b^	MS	SS%	MS	SS%	MS	SS%	MS	SS%	MS	SS%	MS	SS%	MS	SS%	MS	SS%
Environment	2	11.3	14.1	134.1**	22.2	598.3**	13.7	2498.8**	75.9	459.2**	66. 2	5.616**	64.7	0.175**	46.0	15.363**	73.0	1521.1**	70.1
Genotype	7	9.6*	41.8	100.2**	58.2	896.0**	71.9	79.4**	8.4	20.4**	10.3	0.588**	23.7	0.036**	32.7	0.968**	16.1	156.6**	25.3
G × E	14	5.0	44.1	16.9*	19.6	89.3**	14.3	73.9**	15.7	23.3**	23.5	0.144**	11.6	0.012*	21.3	0.328**	10.9	14.3*	4.6
AMMI																			
PC1	8	8.6	97.4	18.2	61.5	98.1	62.8	107.7	83.2	37.8	92.5	0.144	56.8	0.016	77.9	0.475	82.6	21.4	85.3
PC2	6	0.3	2.6	15.2	38.5	77.4	37.2	28.9	16.8	4.1	7.5	0.146	43.2	0.006	22.1	0.133	17.4	4.9	14.7
Residual	42	3.7		8.4		22.8		6.6		1.3		0.034		0.005		0.102		7.2	

^a^ Mean squares; ^b^ Percentage of sum of squares; * P < 0.05; ** P < 0.01

**Fig. 4:**
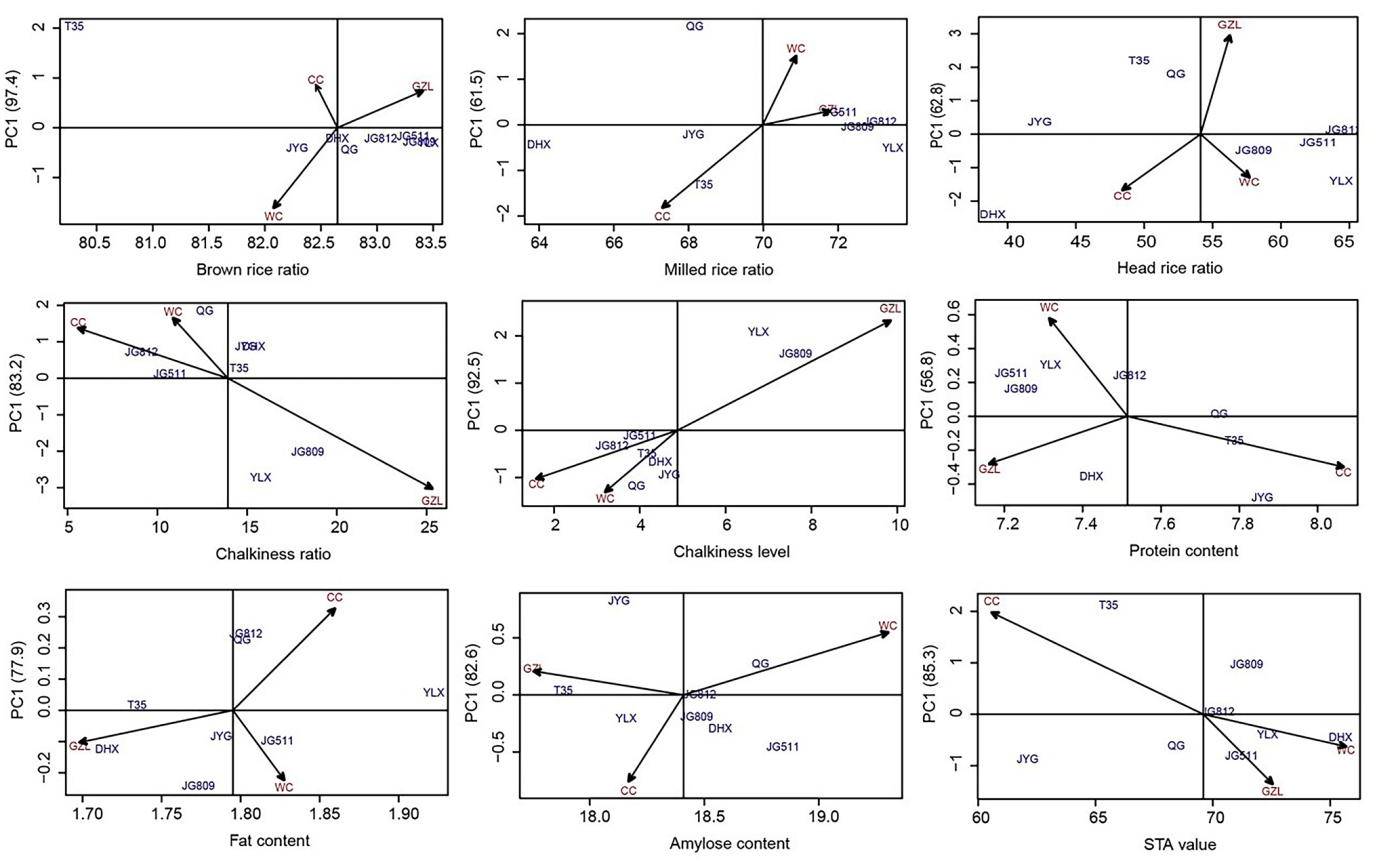
Biplots of the AMMI models. Each biplot was drawn between the mean value of each grain quality trait and the first interactive principal component. The three locations (CC, Changchun city; WC, Wuchang city; GZL, Gongzhuling city) are marked in red, and the eight rice cultivars are marked in blue

The AMMI model biplots also showed that genotypic effects dominated milling quality factors (brown rice ratio, milled rice ratio, and head rice ratio) (Fig. 4). Protein content, fat content, and amylose content were more similar among cultivars grown in Gongzhuling, while STA value was more similar among cultivars grown in Wuchang (Fig. 4). This indicated that some grain quality traits were affected similarly by certain environmental conditions, irrespective of genotype.

### Environment- and genotype-associated transcriptomic changes

**Fig. 5:**
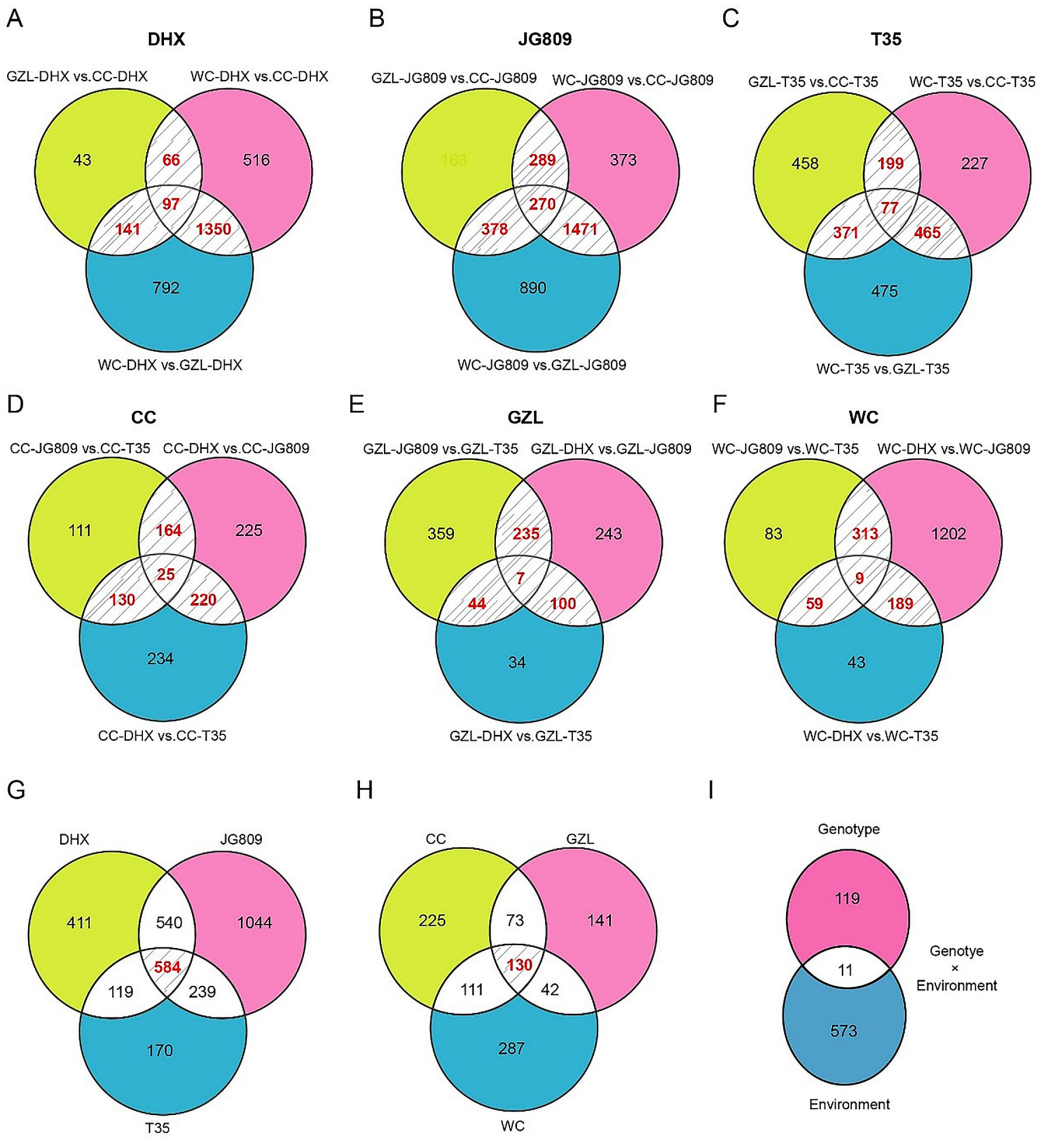
Numbers of differentially expressed genes (DEGs) among genotype and location. **A**–**C**, numbers of DEGs between pairs of locations for each cultivar: **A**, DHX; **B**, JG809; and **C**, T35 (see Supplemental Table 1 for cultivar details). **D–F**, numbers of DEGs between pairs of cultivars at each location: **D**, Changchun city (CC); **E**, Gongzhuling city (GZL); **F**, Wuchang city (WC). Numbers of DEGs shared across cultivars or locations are shown in shaded areas. **G**, Numbers of environment-responsive DEGs shared among genotypes. H, numbers of genotype-responsive DEGs shared among locations. I, numbers of shared DEGs responsive to environment only, genotype only, and genotype × environment interactions

DHX is a well-known good eating quality rice variety with long grain length cultivated in Northeast China. JG809 is also a good-eating quality cultivar but with short grain length. T35 is a relatively low grain quality cultivar. Therefore, we performed RNA-seq analysis for these three cultivars. We first identified the genes differently expressed in the same cultivar in different locations (i.e., the environment-responsive DEGs). In total, 1654 shared DEGs were identified in variety DHX (shaded area in Fig. 5a), 2408 shared DEGs were identified in variety JG809 (shaded area in Fig. 5b), and 1112 shared DEGs were identified in variety T35 (shaded area in Fig. 5c). Then, we identified the genes differentially expressed in different cultivars at the same location (i.e., the genotype-responsive DEGs). In total, 539 shared DEGs were identified across the cultivars grown in Changchun (shaded area in Fig. 5d), 386 shared DEGs were identified across the cultivars grown in Gongzhuling (shaded area in Fig. 5e), and 570 shared DEGs were identified across the cultivars grown in Wuchang (shaded area in Fig. 5f).

Of the 4174 environment-responsive DEGs (Fig. 5a–c), 584 DEGs were shared across the three cultivars (shaded area in Fig. 5g). Of the 1495 genotype-responsive DEGs (Fig. 5d–f), 130 DEGs were shared across the three locations (shaded area in Fig. 5h). Of these environment- or genotype-responsive DEGs, 11 were both environment- and genotype-responsive, 573 were environment-responsive only, and 119 were genotype-responsive only (Fig. 5i; Supplemental Tables 5–7). We defined the 11 environment- and genotype-responsive DEGs as DEGs affected by genotype × environment interactions (Table 2). The relative expression of the 11 DEGs affected by genotype × environment interactions was also analyzed by qRT-PCR and the results were consistent with RNA-seq data, with the correlation coefficient R square ranging from 0.495 to 0.907 (Supplemental Table 8).

**Table 2:** Genes differentially expressed in response to genotype × environment interactions

Gene ID	Annotation
LOC_Os11g18570	Cytochrome P450 family protein
LOC_Os11g09020	Amino acid permease 11C, OsAAP11C
LOC_Os10g01060	Serine/threonine protein kinase-related domain containing protein
LOC_Os09g25150	Cinnamoyl CoA reductase 19
LOC_Os07g47550	UDP-glucosyltransferase
LOC_Os12g08760	Phosphoenolpyruvate kinase
LOC_Os08g43334	Heat stress transcription factor B2b
LOC_Os12g36880	Pathogenesis-related protein PR-10a, OsPR10a
LOC_Os10g41550	OsBAM5, beta-amylase, putative, expressed
LOC_Os09g23540	Cinnamyl alcohol dehydrogenase, OsCAD8B
LOC_Os07g38070	LRR protein kinase

### Functional associations of the DEGs

**Fig. 6:**
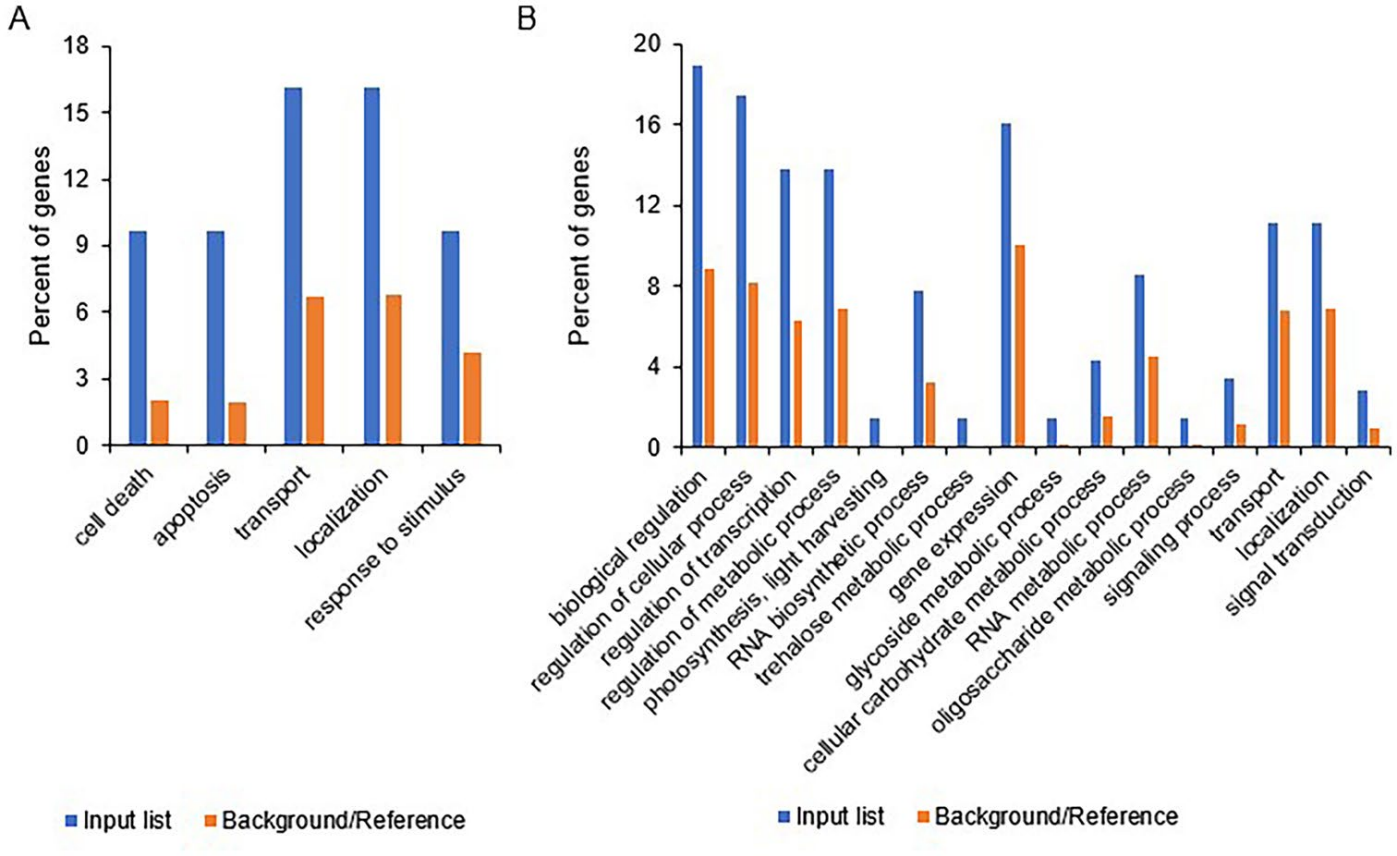
Gene ontology (GO) terms enriched in the shared differentially expressed genes (DEGs). **A** GO terms overrepresented in the 119 genotype-specific DEGs. **B** GO terms overrepresented in the 573 environment-specific DEGs

Only one GO term, “metabolic process” was significantly enriched in the 11 genotype × environment interaction DEGs. After the elimination of redundant GO terms, we found that GO terms related to “cell death”, “apoptosis”, “transport”, “localization”, and “response to stimulus” were significantly enriched in the 119 genotype-responsive DEGs (Fig. 6a). In the 573 environment-responsive DEGs, the significantly enriched GO terms included “cellular carbohydrate metabolic process”, “photosynthesis, light harvesting”, and “regulation of transcription”.

## Discussion

Although Northeast China is an important area for rice production in China, grain quality and yield varies among cultivars and among ecological zones within this territory. Moreover, it is unclear how climate change in this area might threaten the production of high-quality rice. Therefore, it is critical to characterize the effects of environmental factors on various rice cultivars during the grain-filling stage, in order to develop new rice cultivars that produce high-quality rice under various environmental conditions. Here, we found that the mechanisms underlying differences in rice performance which were affected by genotype, environment, and genotype × environment interactions.

Previous studies of rice grown in other regions have shown that air temperature is negatively correlated with amylose content and head rice ratio, but positively correlated with protein content, chalkiness ratio, and chalkiness level (Asaoka et al. 1985; Shi et al. 2016; Xiong et al. 2017; Li et al. 2018). Consistent with these previous studies, we found that, for the cultivars grown in northeast China, amylose content was significantly negatively correlated with average air temperature during the grain-filling stage. Average air temperature was also negatively correlated with head rice ratio, and positively correlated with chalkiness ratio, chalkiness level, and protein content, although these correlations were not significant. STA value, which directly reflects rice eating quality, was significantly negatively correlated with air temperature, but significantly positively correlated with air temperature range. In contrast, protein content was significantly negatively correlated with air temperature range. Therefore, the larger air temperature range during grain filling stage could reduce the protein content and increase the eating quality of rice grains in the Northeast China.

Interestingly, PAR was significantly correlated with more parameters of rice quality than either air temperature or air temperature range. In addition, PAR tended to have the opposite effect on rice quality as compared to air temperature range: PAR was significantly negatively correlated with milled rice ratio, chalkiness ratio, chalkiness level, and STA value, but significantly positively correlated with fat content and protein content. Our path analysis also showed that the interaction between temperature range and PAR strongly affected protein content and STA value. Therefore, the quality of the rice produced in Northeast China was strongly affected by environmental factors, particularly PAR, as well as the interactions among these factors. In general, environmental conditions had a stronger effect on eating quality, while genotype had a stronger effect on milling quality. Importantly, due to the relatively weak correlation between environmental factors and milling quality, rice varieties bred to have high milling quality should produce grains with high milling quality irrespective of environmental conditions. Therefore, it may be efficient to focus on milling quality in particular when breeding rice varieties for Northeast China. In addition, improvement of the cultivation practice and innovations in the breeding technology should be adopted to improve the eating quality of rice in Northeast China.

ANOVA and AMMI analysis showed that different grain quality traits were differentially affected by genotype, environment, and genotype × environment interactions; some of the effects of genotype × environment interactions were significant. We identified more environment-responsive DEGs than genotype-responsive DEGs, suggesting that the environment changes have a dominant role in regulating the grain filling of rice. In addition, many environment-responsive DEGs were shared across genotypes, while many genotype-responsive DEGs were shared across locations.

Many of the GO terms significantly enriched in the 573 DEGs identified as environment responsive (e.g., “cellular carbohydrate metabolic process”, “photosynthesis”, “light harvesting”, and “regulation of transcription”) were related to the response to environmental conditions. This suggests that these genes may be reliably considered sensitive to environmental conditions. For the genotype responsive DEGs, GO terms related to “cell death”, “transport” and “response to stimulus” were significantly enriched, indicating that the differentiation of those genes plays an important role for the grain quality of different cultivars. The GO term "metabolic process," which was the only term identified as significantly enriched in the 11 genotype × environment interaction DEGs, was consistent with the annotations of these DEGs (Table 2). One of these 11 genes, *LOC_Os10g41550*, encodes a beta-amylase: OsBAM5 (Hirano et al. 2016). Beta-amylase plays an important role in starch digestion. Another, *LOC_Os11g09020*, encodes an amino acid permease, which facilitates the transport of amino acids across the cellular membrane (Tegeder and Ward 2012). Due to the important roles played by starch and protein in grain quality, it is unsurprising that the transcriptional regulation of genes involved in starch and protein metabolism underlies the genotype × environment interaction. Two more of the 11 genotype × environment interaction DEGs, *LOC_Os09g25150* and *LOC_Os09g23540*, encode cinnamoyl CoA reductase and cinnamyl alcohol dehydrogenase, respectively; both of these proteins are involved in lignin synthesis (Park et al. 2017; Li et al. 2019). As an important component of dietary fiber, lignin content also affects grain quality in cereals (Marcotuli et al. 2020). Further analysis of the DEGs associated with genotype × environment interactions may provide useful information for the future breeding of high-quality rice varieties adapted to changing global environments.

## Conclusions

By comparing grain quality among eight rice cultivars grown in three different locations in Northeast China, we found that environmental conditions had a stronger effect on eating quality, while genotype had a stronger effect on milling quality. Of the three environmental factors studied herein, PAR was significantly correlated with the most traits associated with grain quality. Using RNA-seq, we identified various genes differentially expressed depending on genotype, environment, and genotype × environment interactions. These DEGs provide useful targets for further studies aiming to breed high-quality rice varieties suitable for cultivation throughout Northeast China.

## Supplementary Information

Below is the link to the electronic supplementary material.

Supplementary file1 (DOCX 308 KB)
