# Supplementary material for: Dissecting the meteorological and genetic factors affecting rice grain quality in Northeast China
Source: Genes Genomics. 2021 Jun 24;43(8):975–86. doi: 10.1007/s13258-021-01121-z (PMC8292277; doi:10.1007/s13258-021-01121-z)
Supplement: Supplementary file 1 — Supplementary file1 (DOCX 308 KB) [file 13258_2021_1121_MOESM1_ESM.docx]

**
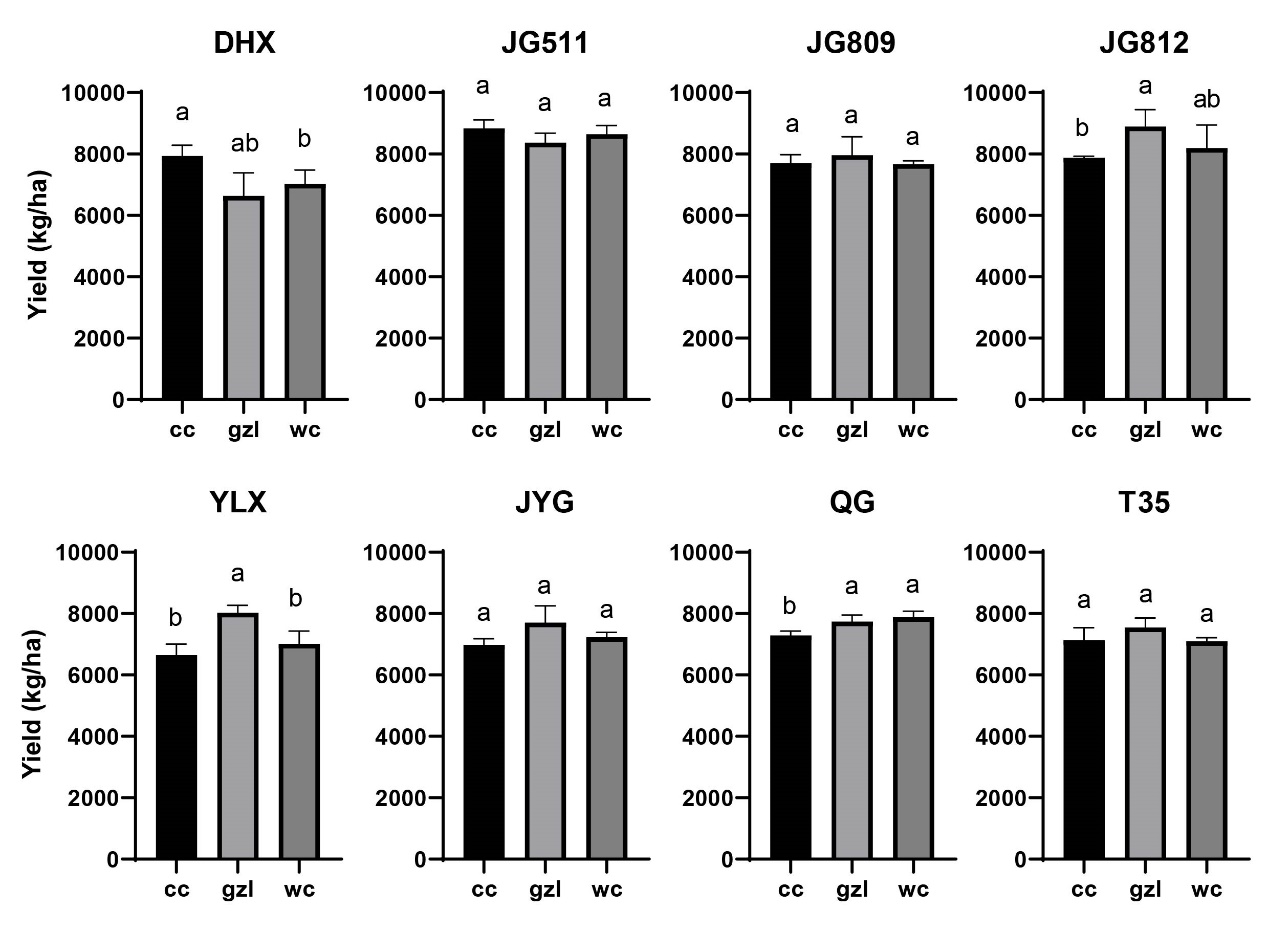
**

**Supplemental Figure 1. Yield performance of rice varieties in 2017.** Different letters indicate significant differences (P < 0.05, Duncan’s new multiple range method, values = means ± SD, n = 3 biological replicates).

| **Supplemental Table 1 Information of selected varieties** | | |  |
| --- | --- | --- | --- |
| Variety name | Abbreviated name | Breeding unit | Eating quality^a^ |
| Jigeng809 | JG809 | Jilin Academy of Agricultural Sciences | Good |
| Jigeng812 | JG812 | Jilin Academy of Agricultural Sciences | Good |
| Jigeng511 | JG511 | Jilin Academy of Agricultural Sciences | Good |
| Wuyoudao4 | DHX | Liyuan Techseeds in Wuchang, Heilongjiang Province | Good |
| Yunlangxiang | YLX | Jilin Academy of Agricultural Sciences | Good |
| Akihikari (Qiuguang) | QG | Bred in Japan, introduced in China by Jilin Academy of Agricultural Sciences | Poor |
| Jiyugeng | JYG | Jilin Academy of Agricultural Sciences | Poor |
| Tong35 | T35 | Tonghua Academy of Agricultural Sciences, Jilin Province | Poor |
| a: the eating quality was concluded according to various results of eating quality tests. | | |  |

**Supplemental Table 2 Grain quality traits in 2017 and 2018**

| Location | Variety | Brown rice ratio (%) | | Milled rice ratio (%) | | Head rice ratio (%) | | Chalkiness ratio (%) | | Chalkiness level | | Protein content (%) | | Fat content (%) | | Amylose content (%) | | STA value | |
| --- | --- | --- | --- | --- | --- | --- | --- | --- | --- | --- | --- | --- | --- | --- | --- | --- | --- | --- | --- |
|  |  | 2017 | 2018 | 2017 | 2018 | 2017 | 2018 | 2017 | 2018 | 2017 | 2018 | 2017 | 2018 | 2017 | 2018 | 2017 | 2018 | 2017 | 2018 |
| CC | DHX | 82.3±0.78 | 78.87±1.65 | 62.37±2.79 | 64.31±2.11 | 39.63±10.63 | 49.03±4.8 | 6.4±1.76 | 27.7±1.73 | 1.5±0.56 | 8.57±0.95 | 7.96±0.14 | 8.66±0.23 | 1.72±0.07 | 1.65±0.08 | 18.62±0.31 | 14.58±1 | 65.33±2.08 | 66±1.73 |
|  | JG511 | 83.03±0.9 | 81.67±0.68 | 68.95±2.48 | 70.57±1.13 | 57.83±10.75 | 67.03±1.01 | 6.07±1.3 | 17.7±0.79 | 2.17±0.38 | 7.3±0.17 | 7.6±0.25 | 8.6±0.15 | 1.85±0.18 | 1.74±0.07 | 18.93±0.28 | 14.8±0.12 | 60.33±5.51 | 64.33±0.58 |
|  | JG809 | 82.67±0.81 | 83.2±0.7 | 70.37±3.71 | 75±0.9 | 51.3±12.3 | 69.67±2.11 | 4.27±0.86 | 13.83±2.16 | 1.4±0.2 | 6.23±0.93 | 7.82±0.23 | 8.24±0.12 | 1.76±0.04 | 1.72±0.09 | 18.34±0.29 | 15.16±0.27 | 64.67±1.15 | 67.33±1.53 |
|  | JG812 | 82.87±0.7 | 81.8±0.52 | 70.59±2.22 | 72.7±1.29 | 55.13±4.04 | 70±1.18 | 3.43±1.67 | 12.2±1.25 | 1.07±0.5 | 5.27±0.21 | 8.05±0.29 | 8.28±0.14 | 1.96±0.09 | 1.61±0.07 | 18.29±0.15 | 14.99±0.14 | 61.67±1.53 | 64.33±2.08 |
|  | JYG | 81.4±1.31 | 82.9±0.3 | 65.61±1.34 | 74.75±0.33 | 40.17±3.45 | 69.73±2.89 | 7.2±5.86 | 31.7±2.78 | 1.83±1.17 | 10.8±0.79 | 8.8±0.21 | 8.69±0.31 | 1.82±0.03 | 1.89±0.08 | 17.19±0.07 | 14.51±0.43 | 51±1 | 46.67±1.53 |
|  | QG | 82.1±1.3 | 81.33±0.57 | 60.66±8.45 | 71.85±0.96 | 39.23±6.17 | 67.47±1.85 | 7.17±4.3 | 17.37±0.85 | 2.03±1.26 | 6.63±0.65 | 8.24±0.36 | 8.4±0.08 | 1.94±0.09 | 1.65±0.04 | 18.26±0.23 | 14.72±0.21 | 58±4.36 | 61±3 |
|  | T35 | 82.03±1.12 | 81.77±0.25 | 67.25±3.02 | 73.25±0.68 | 43.8±8.3 | 66.73±1.83 | 5.37±0.95 | 44.7±1.84 | 1.07±0.55 | 12.87±1.85 | 8.18±0.23 | 8.27±0.1 | 1.81±0.06 | 1.65±0.02 | 17.62±0.81 | 14.37±0.1 | 61±5.57 | 56±1.73 |
|  | YLX | 83.23±0.85 | 82.8±0.53 | 72.63±1.85 | 72.93±0.86 | 60.2±6.62 | 69.37±0.87 | 4.9±3.04 | 16±0.3 | 1.67±0.93 | 6.67±0.06 | 7.87±0.11 | 8.17±0.18 | 2±0.01 | 1.86±0.04 | 18.12±0.39 | 14.33±0.23 | 62.67±4.93 | 67.67±3.06 |
| GZL | DHX | 83.17±0.5 | 81.67±1.38 | 65.12±0.4 | 68.71±1.46 | 33.4±1.71 | 48.1±0.2 | 23.93±4.14 | 26.47±1.45 | 7.77±1.76 | 7.87±0.55 | 7.31±0.4 | 7.97±0.02 | 1.69±0.03 | 1.74±0.11 | 17.69±0.36 | 16.56±0.41 | 78.67±1.53 | 71±0 |
|  | JG511 | 83.87±0.29 | 82.63±0.6 | 73.63±0.73 | 74.3±0.74 | 64.1±2.16 | 65.97±0.93 | 21.43±2.91 | 30.27±2.34 | 8.57±1.36 | 12±1.18 | 6.88±0.3 | 7.48±0.12 | 1.75±0.01 | 1.79±0.04 | 18.28±0.04 | 16.95±0.85 | 75±5.2 | 70.33±2.52 |
|  | JG809 | 84.17±0.42 | 82.93±0.81 | 73.04±1.61 | 75.62±0.55 | 58.4±6.54 | 67.23±1.5 | 36.67±3.75 | 17.6±0.95 | 16.87±3.74 | 7.03±0.59 | 6.76±0.07 | 7.77±0.18 | 1.66±0.04 | 1.7±0.07 | 17.96±0.55 | 16.58±0.69 | 73.33±0.58 | 70±0 |
|  | JG812 | 83.37±0.51 | 83.43±0.51 | 74.16±1.18 | 74.86±2.22 | 66.87±2.99 | 69.33±2.74 | 18±1.14 | 14.8±3.7 | 7.43±0.95 | 5.8±1.5 | 7.03±0.14 | 7.79±0.05 | 1.65±0.05 | 1.65±0.1 | 17.63±0.25 | 16.79±0.27 | 74.33±2.31 | 66±1 |
|  | JYG | 83.03±0.74 | 82.1±0.36 | 70.59±1.85 | 69.1±0.38 | 46.07±3.56 | 43.9±5.51 | 23.43±2.4 | 54.07±4.05 | 7.3±0.95 | 18.9±2.1 | 7.42±0.15 | 7.92±0.32 | 1.7±0.07 | 1.89±0.01 | 17.7±0.23 | 15.58±0.67 | 65.67±3.06 | 49±1 |
|  | QG | 83.2±0.1 | 83±0.3 | 72.04±1.09 | 71.91±0.57 | 59.97±3.1 | 57.73±1.02 | 17.8±1.61 | 27.57±0.45 | 5.83±1.23 | 9.27±0.25 | 7.45±0.3 | 7.49±0.23 | 1.7±0.13 | 1.49±0.19 | 18.24±0.31 | 16.84±0.69 | 72±0 | 65±3.61 |
|  | T35 | 82.77±0.06 | 81.07±1.01 | 72.81±0.53 | 71.4±2.18 | 59.33±1.57 | 58.5±4.5 | 25.1±3.96 | 49.77±3.15 | 7.9±1.23 | 16.37±1.85 | 7.69±0.04 | 7.75±0.11 | 1.61±0.05 | 1.71±0.05 | 17.22±0.34 | 15.81±0.68 | 64.67±2.08 | 56±2 |
|  | YLX | 83.7±0.17 | 82.57±0.21 | 72.77±0.5 | 73.63±1.07 | 61.9±5.55 | 64.8±3.52 | 36.13±2.97 | 30.03±3.66 | 17.07±1.64 | 13.27±2.04 | 6.76±0.15 | 7.31±0.03 | 1.84±0.03 | 1.81±0.03 | 17.34±0.14 | 16.45±0.62 | 76.67±3.21 | 69±1 |
| WC | DHX | 82.47±0.21 | 82.17±0.31 | 64.48±1.53 | 65.07±1.59 | 43.73±1.76 | 48.1±3.06 | 15.77±1.46 | 16.17±3.29 | 4.17±0.35 | 4.03±0.67 | 6.99±0.23 | 7.74±0.16 | 1.74±0.04 | 1.85±0.07 | 19.41±0.38 | 16.85±0.72 | 82.33±4.04 | 76±2.65 |
|  | JG511 | 83.07±0.21 | 82.93±0.25 | 73.64±0.42 | 74.89±1.05 | 66.23±0.91 | 66.67±1.78 | 4.6±0.4 | 4.67±0.68 | 1.27±0.15 | 1.2±0.53 | 7.18±0.04 | 7.78±0.11 | 1.87±0.08 | 1.79±0.14 | 19.32±0.14 | 16.91±0.3 | 78.33±3.06 | 72±0 |
|  | JG809 | 83.3±0.52 | 82.03±0.46 | 74.15±0.74 | 74.04±0.59 | 64.3±0.3 | 71.53±3.06 | 14.2±1.39 | 4.47±2.63 | 4.63±0.4 | 2±1.31 | 7.15±0.32 | 7.46±0.36 | 1.9±0.08 | 1.75±0.02 | 19.1±0.69 | 16.76±0.62 | 76.33±1.53 | 71.67±3.21 |
|  | JG812 | 82.87±0.49 | 82.33±0.64 | 74.63±0.52 | 74.37±1.38 | 71.73±0.47 | 69.33±2.24 | 5.93±0.21 | 3.87±0.55 | 1.57±0.23 | 1.2±0 | 7.48±0.28 | 7.66±0.08 | 1.8±0.05 | 1.71±0.09 | 19.52±0.22 | 17.08±0.22 | 74.67±1.53 | 73.67±1.53 |
|  | JYG | 82.43±0.42 | 81.2±0.36 | 68.19±0.31 | 69.59±0.81 | 40.87±2.06 | 56.4±4.08 | 14.17±0.86 | 20.07±2.51 | 4.93±0.6 | 5.17±0.76 | 7.36±0.11 | 7.92±0.15 | 1.84±0.09 | 1.82±0.05 | 19.5±0.15 | 15.89±0.29 | 69.67±2.31 | 61.33±1.53 |
|  | QG | 82.97±1.07 | 82.03±0.12 | 71.8±1.01 | 67.49±2.18 | 57.77±0.47 | 53.8±1.75 | 12.97±0.76 | 12.2±0.52 | 3.9±0.46 | 3.1±0.44 | 7.56±0.24 | 7.42±0.16 | 1.76±0.09 | 1.7±0.07 | 19.74±0.32 | 16.88±0.45 | 75.33±1.53 | 74±1 |
|  | T35 | 76.1±9.18 | 80.37±0.4 | 65.12±7.95 | 69.67±0.56 | 45.73±4.9 | 53.83±4.05 | 13.2±2.43 | 21.23±3.9 | 3.53±0.64 | 4.7±0.8 | 7.49±0.28 | 7.64±0.2 | 1.78±0.05 | 1.73±0.12 | 18.82±0.39 | 15.98±0.47 | 71±2.65 | 58.67±3.21 |
|  | YLX | 83.43±0.32 | 83.3±0.17 | 74.99±0.33 | 73.58±1.33 | 71.13±1.16 | 70.57±2.06 | 6.23±1.62 | 3.97±1.11 | 1.57±0.45 | 1±0.52 | 7.32±0.17 | 7.64±0.13 | 1.93±0.04 | 1.89±0.09 | 19.02±0.35 | 15.93±0.79 | 77.67±1.53 | 71.33±3.51 |

**Supplemental Table 3. Correlation analysis between the data of 2017 and 2018**

| Data term | Correlation | *P* value |
| --- | --- | --- |
| Average Temperature | 0.956 | 5.07E-11 |
| Range of Temperature | 0.612 | 0.0089 |
| Photosynthetically Active Radiation | 0.695 | 1.38E-03 |
| Brown Rice Ratio | 0.474 | 0.059 |
| Milled Rice Ratio | 0.599 | 0.0105 |
| Head Rice Ratio | 0.558 | 0.021 |
| Chalkiness Ratio | 0.399 | 0.12 |
| Chalkiness Level | 0.401 | 0.12 |
| Protein Content | 0.768 | 1.42E-04 |
| Fat Content | 0.235 | 0.415 |
| Amylose Content | 0.341 | 0.212 |
| STA Value | 0.763 | 0.000158 |

**Supplemental Table 4. Path analysis of the environmental factors to rice grain quality.**

| Brown rice ratio | Correlation coefficient | Direct effect | Indirect effect path coefficients | | |
| --- | --- | --- | --- | --- | --- |
|  |  |  | Average Temperature | Range of Temperature | Photosynthetically Active Radiation |
| Average Temperature | -0.115 | -0.686 |  | 0.843 | -0.277 |
| Range of Temperature | 0.192 | -1.240 | 0.467 |  | 0.963 |
| Photosynthetically Active Radiation | -0.373 | -1.204 | -0.158 | 0.992 |  |
| Residual Effect^2 = 0.708 | | | | | |
| Milled rice ratio | Correlation coefficient | Direct effect | Indirect effect path coefficients | | |
|  |  |  | Average Temperature | Range of Temperature | Photosynthetically Active Radiation |
| Average Temperature | -0.289 | -0.521 |  | 0.443 | -0.212 |
| Range of Temperature | 0.441 | -0.651 | 0.354 |  | 0.737 |
| Photosynthetically Active Radiation | -0.518* | -0.921 | -0.120 | 0.521 |  |
| Residual Effect^2 = 0.656 | | | | | |
| Head rice ratio | Correlation coefficient | Direct effect | Indirect effect path coefficients | | |
|  |  |  | Average Temperature | Range of Temperature | Photosynthetically Active Radiation |
| AverageTemperature | -0.474 | -0.873 |  | 0.621 | -0.218 |
| RangeTemperature | 0.444 | -0.913 | 0.593 |  | 0.760 |
| Photosynthetically Active Radiation | -0.418 | -0.950 | -0.201 | 0.731 |  |
| Residual Effect^2 = 0.593 | | | | | |
| Chalkiness Ratio | Correlation coefficient | Direct effect | Indirect effect path coefficients | | |
|  |  |  | Average Temperature | Range of Temperature | Photosynthetically Active Radiation |
| AverageTemperature | 0.246 | 0.596 |  | -0.188 | -0.158 |
| RangeTemperature | 0.42 | 0.276 | -0.405 |  | 0.549 |
| Photosynthetically Active Radiation | -0.772** | -0.686 | 0.137 | -0.221 |  |
| Residual Effect^2 = 0.207 | | | | | |
| Chalkiness Level | Correlation coefficient | Direct effect | Indirect effect path coefficients | | |
|  |  |  | Average Temperature | Range of Temperature | Photosynthetically Active Radiation |
| AverageTemperature | 0.234 | 0.286 |  | 0.174 | -0.230 |
| RangeTemperature | 0.352 | -0.256 | -0.195 |  | 0.800 |
| Photosynthetically Active Radiation | -0.727** | -1.000 | 0.066 | 0.204 |  |
| Residual Effect^2 = 0.293 | | | | | |
| Protein Content | Correlation coefficient | Direct effect | Indirect effect path coefficients | | |
|  |  |  | Average Temperature | Range of Temperature | Photosynthetically Active Radiation |
| AverageTemperature | 0.291 | 0.141 |  | -0.037 | 0.187 |
| RangeTemperature | -0.688** | 0.055 | -0.096 |  | -0.649 |
| Photosynthetically Active Radiation | 0.798** | 0.812 | 0.032 | -0.044 |  |
| Residual Effect^2 = 0.348 | | | | | |
| Fat Content | Correlation coefficient | Direct effect | Indirect effect path coefficients | | |
|  |  |  | Average Temperature | Range of Temperature | Photosynthetically Active Radiation |
| AverageTemperature | -0.204 | -0.427 |  | 0.094 | 0.133 |
| RangeTemperature | -0.311 | -0.138 | 0.290 |  | -0.462 |
| Photosynthetically Active Radiation | 0.587** | 0.578 | -0.098 | 0.110 |  |
| Residual Effect^2 = 0.531 | | | | | |
| Amylose Content | Correlation coefficient | Direct effect | Indirect effect path coefficients | | |
|  |  |  | Average Temperature | Range of Temperature | Photosynthetically Active Radiation |
| AverageTemperature | -0.740** | -0.581 |  | -0.299 | 0.139 |
| RangeTemperature | 0.355 | 0.439 | 0.395 |  | -0.484 |
| Photosynthetically Active Radiation | 0.12 | 0.605 | -0.134 | -0.351 |  |
| Residual Effect^2 = 0.344 | | | | | |
| STA Value | Correlation coefficient | Direct effect | Indirect effect path coefficients | | |
|  |  |  | Average Temperature | Range of Temperature | Photosynthetically Active Radiation |
| AverageTemperature | -0.464 | -0.232 |  | -0.115 | -0.113 |
| RangeTemperature | 0.721** | 0.170 | 0.158 |  | 0.393 |
| Photosynthetically Active Radiation | -0.678** | -0.491 | -0.053 | -0.136 |  |
| Residual Effect^2 = 0.437 | | | | | |

**Supplemental Table 5. DEGs affected by genotype × environment interaction**

| GeneName | ccT35 | ccDHX | ccJG809 | gzlT35 | gzlDHX | gzlJG809 | wcT35 | wcDHX | wcJG809 |
| --- | --- | --- | --- | --- | --- | --- | --- | --- | --- |
| LOC_Os11g18570 | 0.361539 | 1.004012 | 2.641211 | 6.648018 | 2.063102 | 5.604852 | 0.267799 | 0.165038 | 0.925119 |
| LOC_Os11g09020 | 7.755746 | 8.302721 | 16.78531 | 16.28815 | 12.96856 | 28.20348 | 1.522343 | 1.232383 | 15.41795 |
| LOC_Os10g01060 | 1.751227 | 1.453851 | 0.485863 | 1.363961 | 1.3279 | 0.397493 | 5.76747 | 7.926626 | 3.72916 |
| LOC_Os09g25150 | 4.047402 | 2.826423 | 14.05523 | 1.488216 | 1.106466 | 7.803015 | 0.744547 | 1.09548 | 3.161921 |
| LOC_Os07g47550 | 3.690773 | 2.937185 | 1.472346 | 0.943914 | 0.912541 | 0.335682 | 0.74002 | 1.055976 | 0.292218 |
| LOC_Os12g08760 | 9.196433 | 20.93714 | 3.771097 | 12.98937 | 11.33194 | 1.415862 | 1.56065 | 1.00803 | 0.397208 |
| LOC_Os08g43334 | 22.26704 | 13.7925 | 7.873304 | 12.40834 | 12.44109 | 5.307941 | 1.562487 | 2.659973 | 0.876074 |
| LOC_Os12g36880 | 2.365245 | 21.92449 | 2.417708 | 29.06989 | 93.06533 | 19.32785 | 2.556581 | 8.938933 | 14.22475 |
| LOC_Os10g41550 | 9.951816 | 5.605369 | 9.518979 | 8.443276 | 3.608059 | 3.957136 | 29.20304 | 31.64935 | 60.06172 |
| LOC_Os09g23540 | 0.215199 | 1.763221 | 0.482262 | 2.378543 | 6.57263 | 1.922072 | 0.919065 | 2.369834 | 1.788414 |
| LOC_Os07g38070 | 0.764827 | 0.305168 | 0.813481 | 0.987609 | 0.320871 | 0.561853 | 3.846699 | 0.956195 | 2.847834 |

**Supplemental Table 6. Environmentally specific DEGs.**

| Gene ID | ccT35 | ccDHX | ccJG809 | gzlT35 | gzlDHX | gzlJG809 | wcT35 | wcDHX | wcJG809 |
| --- | --- | --- | --- | --- | --- | --- | --- | --- | --- |
| LOC_Os12g12560 | 2.403753 | 3.687208 | 2.715533 | 1.281517 | 1.028678 | 0.886964 | 0.869805 | 1.385413 | 1.007958 |
| LOC_Os12g02130 | 11.65391 | 9.998555 | 10.75034 | 8.930021 | 7.560883 | 8.348925 | 4.891822 | 5.773737 | 5.83241 |
| LOC_Os12g01740 | 8.162464 | 7.776723 | 5.780192 | 5.66897 | 4.673165 | 3.451665 | 2.597138 | 2.415575 | 2.216598 |
| LOC_Os11g43820 | 45.93284 | 36.2602 | 43.45853 | 25.88157 | 24.19787 | 34.21448 | 20.75376 | 21.04015 | 20.06652 |
| LOC_Os11g42510 | 4.527351 | 4.22432 | 5.865209 | 3.770855 | 2.279589 | 3.717657 | 1.868381 | 2.016536 | 3.554494 |
| LOC_Os11g05470 | 1.409578 | 1.498603 | 1.055309 | 0.676914 | 0.690002 | 1.397906 | 0.164612 | 0.287918 | 0.266664 |
| LOC_Os11g02190 | 13.30272 | 10.83494 | 11.67768 | 9.232376 | 8.049974 | 8.691118 | 5.219338 | 6.621069 | 6.265549 |
| LOC_Os11g01740 | 11.71905 | 9.168839 | 9.81622 | 6.883711 | 5.717136 | 5.698745 | 4.717895 | 4.050829 | 4.241722 |
| LOC_Os10g30850 | 1.244597 | 1.691238 | 2.069693 | 3.499953 | 4.253414 | 5.624126 | 5.600842 | 3.465819 | 4.98309 |
| LOC_Os10g21460 | 1.720349 | 2.405408 | 2.53558 | 6.542525 | 6.277171 | 6.615176 | 3.685334 | 3.016675 | 5.977161 |
| LOC_Os10g11310 | 0.662936 | 1.172878 | 0.539274 | 0.369625 | 0.587846 | 0.237731 | 0.070304 | 0.519565 | 0.243801 |
| LOC_Os09g26780 | 22.92297 | 32.51347 | 23.50897 | 15.59079 | 17.35909 | 20.8476 | 8.588204 | 11.08027 | 9.86894 |
| LOC_Os09g24980 | 3.282812 | 2.960709 | 3.581122 | 2.611069 | 1.929597 | 1.843172 | 1.160338 | 1.260094 | 2.126569 |
| LOC_Os09g24710 | 1.866132 | 3.413778 | 3.0241 | 2.760721 | 2.501389 | 2.643687 | 0.664332 | 1.003681 | 0.892618 |
| LOC_Os09g23550 | 0.20411 | 0.856348 | 0.344514 | 2.002906 | 3.322883 | 1.659058 | 0.834336 | 1.656278 | 1.851426 |
| LOC_Os09g04160 | 0.422116 | 0.472278 | 0.616545 | 2.633332 | 1.55268 | 1.459999 | 1.207509 | 0.943006 | 1.185015 |
| LOC_Os08g34190 | 47.14031 | 45.23255 | 51.25634 | 27.93412 | 29.61421 | 32.02677 | 20.85999 | 22.00475 | 41.87301 |
| LOC_Os08g29570 | 0.267098 | 0.317492 | 0.301608 | 0.626465 | 1.160211 | 0.657393 | 0.819581 | 0.957939 | 0.948457 |
| LOC_Os07g44140 | 1.141131 | 1.183205 | 1.167932 | 1.879448 | 3.294138 | 1.472977 | 2.7579 | 2.958464 | 2.739531 |
| LOC_Os07g43810 | 8.430266 | 8.805876 | 5.628822 | 11.30622 | 16.11034 | 17.26353 | 11.8232 | 9.957535 | 16.66205 |
| LOC_Os07g38380 | 31.67414 | 22.62341 | 24.96349 | 12.30545 | 10.30633 | 13.37511 | 12.03124 | 12.78445 | 8.587341 |
| LOC_Os07g36820 | 10.93543 | 9.37628 | 9.000285 | 6.389538 | 5.157824 | 4.834595 | 3.314173 | 4.896937 | 4.560854 |
| LOC_Os07g09420 | 8.701545 | 12.83773 | 9.89864 | 6.608915 | 7.902972 | 6.41681 | 4.389024 | 6.573316 | 10.37716 |
| LOC_Os07g08150 | 30.74883 | 77.23016 | 56.62132 | 26.90235 | 25.46559 | 32.83756 | 10.38267 | 19.89851 | 50.1308 |
| LOC_Os06g49660 | 1.993901 | 1.772713 | 2.011274 | 3.730807 | 0.457439 | 0.807155 | 0.17609 | 0.174013 | 0.067055 |
| LOC_Os06g43930 | 0.618685 | 1.504088 | 1.128842 | 3.272623 | 4.218191 | 3.058678 | 1.527327 | 1.936682 | 3.062768 |
| LOC_Os06g31060 | 132.4211 | 93.61105 | 137.9776 | 62.45653 | 40.42195 | 45.713 | 25.71781 | 42.68891 | 31.61569 |
| LOC_Os06g24490 | 11.38939 | 12.10286 | 10.0423 | 4.407802 | 5.461997 | 5.042395 | 7.179627 | 7.119303 | 6.643889 |
| LOC_Os06g16350 | 2.762623 | 2.541821 | 2.175847 | 0.566565 | 0.486353 | 0.267135 | 0.506296 | 0.530312 | 0.407275 |
| LOC_Os06g14324 | 4.098037 | 7.200106 | 7.300803 | 6.986979 | 4.162139 | 3.427538 | 1.4002 | 2.205061 | 2.801125 |
| LOC_Os06g12370 | 3.230093 | 1.70871 | 2.302291 | 1.303391 | 0.580594 | 0.448187 | 0.546826 | 0.702254 | 0.35943 |
| LOC_Os12g44090 | 1.116249 | 2.27552 | 0.407688 | 4.852435 | 5.712013 | 1.399278 | 0.71641 | 0.39626 | 0.35403 |
| LOC_Os12g42570 | 7.073204 | 6.944609 | 5.550912 | 4.555202 | 3.736583 | 3.655225 | 5.582498 | 9.993809 | 5.521094 |
| LOC_Os12g15400 | 1.381197 | 1.04689 | 1.717909 | 3.634465 | 2.564402 | 2.920263 | 3.614613 | 2.63562 | 5.146151 |
| LOC_Os12g13670 | 7.784519 | 7.595999 | 6.682267 | 3.716781 | 3.925492 | 4.91038 | 2.820849 | 4.197584 | 3.186661 |
| LOC_Os12g08810 | 14.49478 | 20.52693 | 13.41746 | 14.02538 | 8.673667 | 9.565433 | 14.87956 | 24.08901 | 17.03662 |
| LOC_Os12g02250 | 6.370333 | 6.353222 | 4.421624 | 4.12141 | 2.892564 | 3.635482 | 4.971481 | 7.285003 | 2.522355 |
| LOC_Os11g38870 | 2.996223 | 2.856284 | 2.400692 | 2.212204 | 2.033832 | 1.730654 | 3.117929 | 3.255185 | 5.377734 |
| LOC_Os11g31740 | 0.535513 | 0.59694 | 0.461919 | 1.922767 | 1.838416 | 0.597152 | 0.122464 | 0.170594 | 0.100657 |
| LOC_Os11g26760 | 32.27086 | 17.29161 | 32.08058 | 15.96144 | 9.098573 | 9.941076 | 36.33191 | 53.40424 | 48.43354 |
| LOC_Os11g05400 | 18.95554 | 8.879215 | 13.11801 | 4.273582 | 3.563356 | 5.347187 | 25.86061 | 19.76856 | 20.78806 |
| LOC_Os10g41820 | 1.180357 | 1.683243 | 1.238669 | 5.093731 | 4.355268 | 2.255403 | 0.676893 | 0.842131 | 0.746592 |
| LOC_Os10g38870 | 9.864724 | 7.120771 | 8.76384 | 2.470712 | 1.256824 | 2.598757 | 0.98313 | 2.737249 | 0.814751 |
| LOC_Os10g35770 | 27.10686 | 29.62162 | 26.1167 | 24.30965 | 19.17002 | 21.71005 | 31.49984 | 33.27247 | 29.38818 |
| LOC_Os10g32980 | 1.049475 | 0.861923 | 0.945002 | 0.283979 | 0.342896 | 0.81012 | 2.248234 | 0.999137 | 1.537062 |
| LOC_Os10g31530 | 0.405443 | 0.761911 | 0.614465 | 3.578204 | 3.012801 | 2.968676 | 0.149865 | 0.183081 | 0.585466 |
| LOC_Os10g25850 | 6.800788 | 4.726162 | 8.155669 | 18.28353 | 12.38124 | 17.06814 | 1.803113 | 2.278185 | 4.163635 |
| LOC_Os10g21590 | 1.171186 | 1.338342 | 0.709506 | 0.797112 | 0.742831 | 0.506313 | 6.29421 | 5.243456 | 3.065077 |
| LOC_Os10g18370 | 6.375768 | 7.564463 | 5.65108 | 2.362216 | 1.384125 | 1.542006 | 5.58625 | 12.34585 | 11.85145 |
| LOC_Os10g11580 | 55.87786 | 49.76711 | 61.53204 | 126.7142 | 129.7978 | 138.033 | 27.64714 | 20.61303 | 57.98762 |
| LOC_Os09g38772 | 6.834209 | 10.52724 | 9.232489 | 16.869 | 19.78986 | 18.60863 | 4.551484 | 4.456567 | 8.675125 |
| LOC_Os09g38390 | 5.828128 | 5.715565 | 5.52686 | 3.809001 | 3.036142 | 4.061341 | 4.952475 | 7.242551 | 6.040313 |
| LOC_Os09g29930 | 12.43644 | 11.21174 | 10.85607 | 8.410477 | 7.890072 | 8.993682 | 17.30129 | 17.88019 | 23.71503 |
| LOC_Os09g28220 | 10.49987 | 10.13668 | 9.430384 | 8.92093 | 8.238738 | 7.261143 | 9.47591 | 13.9687 | 17.27888 |
| LOC_Os09g11480 | 1.376388 | 1.802869 | 3.150286 | 4.904994 | 4.425748 | 4.665652 | 0.698761 | 0.399117 | 0.496986 |
| LOC_Os09g11250 | 1.997753 | 2.634571 | 2.762909 | 5.245048 | 5.668888 | 3.525229 | 1.344539 | 1.083727 | 1.039655 |
| LOC_Os08g40200 | 5.933307 | 5.436304 | 6.031852 | 4.58133 | 4.133562 | 5.128011 | 6.513177 | 5.591109 | 8.495597 |
| LOC_Os08g39300 | 3.231399 | 8.2169 | 5.828483 | 14.52215 | 5.990713 | 7.817234 | 10.70117 | 16.68867 | 15.78571 |
| LOC_Os08g38210 | 14.87872 | 14.10203 | 12.47869 | 8.335237 | 7.08054 | 9.943812 | 17.04381 | 18.07144 | 18.94529 |
| LOC_Os08g38170 | 2.279091 | 1.652618 | 1.708529 | 0.775728 | 0.402091 | 0.13457 | 0.016809 | 0.011606 | 0.070407 |
| LOC_Os08g37350 | 7.374237 | 8.245864 | 7.47553 | 6.305244 | 5.98827 | 6.230602 | 6.550223 | 10.00044 | 11.42493 |
| LOC_Os08g19170 | 5.382805 | 7.061765 | 5.691459 | 4.490597 | 5.447529 | 3.993665 | 9.01924 | 22.76484 | 13.88752 |
| LOC_Os08g15230 | 10.33732 | 8.081698 | 8.738574 | 6.458505 | 5.718279 | 10.07816 | 12.78969 | 12.06013 | 16.56036 |
| LOC_Os08g14330 | 4.385868 | 5.593588 | 5.84766 | 8.915524 | 9.581891 | 8.087511 | 2.51123 | 2.590783 | 3.535972 |
| LOC_Os08g09690 | 6.905445 | 8.494878 | 6.871387 | 15.85363 | 16.45375 | 11.36561 | 2.063807 | 2.38309 | 2.175385 |
| LOC_Os08g01410 | 5.584483 | 4.444769 | 5.262426 | 2.652643 | 1.85869 | 2.149776 | 2.504895 | 5.209299 | 2.287189 |
| LOC_Os07g48430 | 11.33181 | 11.1545 | 15.07073 | 8.104312 | 7.936198 | 9.029253 | 11.68456 | 11.54702 | 15.32777 |
| LOC_Os07g46240 | 12.49883 | 14.12951 | 15.79151 | 8.522723 | 9.36498 | 13.23999 | 15.00481 | 17.27467 | 21.67515 |
| LOC_Os07g43740 | 7.602185 | 6.229478 | 5.658916 | 18.545 | 20.27378 | 15.72128 | 1.107214 | 1.835252 | 0.887977 |
| LOC_Os07g43700 | 0.452992 | 0.901088 | 0.792749 | 0.962972 | 0.447134 | 0.640875 | 0.088055 | 0.036594 | 0.223756 |
| LOC_Os07g43390 | 13.9162 | 11.57455 | 13.445 | 12.12459 | 9.804191 | 10.36122 | 10.47954 | 11.75029 | 14.72022 |
| LOC_Os07g34520 | 7.759221 | 6.238641 | 3.991243 | 1.718908 | 1.56491 | 1.008067 | 6.135111 | 6.333359 | 2.960331 |
| LOC_Os07g34510 | 2.312549 | 1.746837 | 1.483523 | 1.090202 | 0.779275 | 1.003104 | 3.084017 | 2.093848 | 1.174434 |
| LOC_Os07g31650 | 8.06435 | 7.963939 | 6.79589 | 2.254245 | 2.660838 | 2.475691 | 9.57965 | 10.73679 | 8.625452 |
| LOC_Os07g30960 | 1.444337 | 1.861946 | 1.655338 | 0.769426 | 0.870439 | 1.134946 | 3.830942 | 2.352542 | 3.403992 |
| LOC_Os07g28400 | 13.6447 | 13.32425 | 14.55252 | 11.12628 | 8.069978 | 10.99452 | 15.73653 | 13.64536 | 17.70992 |
| LOC_Os07g26940 | 11.03569 | 14.62861 | 19.34041 | 24.75866 | 29.23015 | 24.46847 | 8.83238 | 7.915914 | 11.74675 |
| LOC_Os07g07080 | 18.79063 | 14.25854 | 17.82568 | 9.899765 | 9.454831 | 8.898063 | 44.34714 | 56.54042 | 71.44394 |
| LOC_Os07g02800 | 9.235728 | 9.98559 | 11.89527 | 8.397586 | 6.451725 | 8.512252 | 35.50609 | 37.43504 | 31.62419 |
| LOC_Os06g51390 | 6.379239 | 5.723942 | 4.614107 | 4.972109 | 4.208556 | 3.535324 | 17.92964 | 17.31402 | 14.74211 |
| LOC_Os06g49860 | 0.772912 | 1.257054 | 0.573985 | 0.325288 | 0.659415 | 0.209065 | 0.070173 | 0.187576 | 0.110818 |
| LOC_Os06g45090 | 20.21069 | 14.10486 | 15.55743 | 8.657189 | 7.648375 | 8.533129 | 16.19103 | 20.12723 | 22.22306 |
| LOC_Os06g45040 | 2.485334 | 1.618739 | 1.551801 | 1.001745 | 0.724685 | 0.867858 | 2.247467 | 2.503761 | 3.162722 |
| LOC_Os06g44450 | 8.489838 | 11.62032 | 9.235728 | 11.05414 | 7.810308 | 7.865553 | 17.79222 | 23.80872 | 28.89832 |
| LOC_Os06g33880 | 1.767995 | 3.705974 | 4.308523 | 8.898131 | 9.825546 | 13.03548 | 1.571199 | 1.541898 | 6.420418 |
| LOC_Os06g14420 | 6.836377 | 2.413111 | 4.89117 | 21.528 | 11.92377 | 6.305659 | 0.863672 | 0.766349 | 1.322428 |
| LOC_Os12g19290 | 5.805212 | 5.564333 | 5.415951 | 4.202237 | 4.228752 | 3.980633 | 5.350026 | 4.919543 | 6.753217 |
| LOC_Os12g16720 | 1.190223 | 1.448387 | 1.030914 | 0.702467 | 0.58395 | 0.523159 | 1.291783 | 1.425291 | 1.08762 |
| LOC_Os12g14070 | 694.0168 | 657.9641 | 688.1962 | 392.0857 | 429.3008 | 691.8442 | 639.7358 | 601.0275 | 937.3179 |
| LOC_Os12g07820 | 3.295885 | 4.520863 | 2.848433 | 10.14348 | 8.175889 | 5.350527 | 5.328949 | 3.173077 | 4.170949 |
| LOC_Os11g42490 | 0.962069 | 1.30691 | 1.288385 | 0.464978 | 0.325373 | 0.447282 | 0.766221 | 1.070611 | 1.227795 |
| LOC_Os11g40590 | 41.26674 | 43.11734 | 32.25608 | 31.30348 | 28.1849 | 44.10852 | 34.18221 | 28.27742 | 67.56346 |
| LOC_Os11g38980 | 8.688922 | 10.92759 | 7.283441 | 4.834366 | 5.036943 | 4.705289 | 3.606218 | 8.544064 | 5.881699 |
| LOC_Os11g34460 | 1.351766 | 3.326097 | 2.322384 | 5.649644 | 9.492308 | 11.10563 | 2.196541 | 2.623572 | 2.703002 |
| LOC_Os11g05820 | 0.867105 | 1.275409 | 1.651191 | 2.244453 | 2.585395 | 1.29431 | 0.669129 | 0.846425 | 0.664797 |
| LOC_Os10g40100 | 0.807707 | 1.140415 | 1.038853 | 3.41272 | 3.122231 | 1.964512 | 0.604513 | 0.5975 | 0.927195 |
| LOC_Os10g37660 | 2.448576 | 2.089585 | 1.543911 | 0.738334 | 0.708819 | 0.623197 | 1.410057 | 2.006565 | 2.332958 |
| LOC_Os10g37400 | 10.92623 | 7.818908 | 6.472959 | 3.772919 | 5.070868 | 1.282135 | 7.953108 | 6.131991 | 4.133813 |
| LOC_Os10g34020 | 0.435397 | 0.863845 | 0.988085 | 2.909092 | 2.396254 | 2.341091 | 0.610297 | 0.35507 | 1.134744 |
| LOC_Os09g35030 | 1.418723 | 0.767296 | 0.859665 | 6.412916 | 3.962581 | 2.483713 | 0.559444 | 0.292623 | 0.14978 |
| LOC_Os09g33780 | 13.3136 | 11.50287 | 9.031143 | 7.228014 | 6.731111 | 6.07237 | 5.302482 | 7.929892 | 12.77686 |
| LOC_Os09g27220 | 11.79434 | 6.734995 | 8.41655 | 5.776708 | 4.969088 | 4.226244 | 7.120481 | 6.245963 | 6.790681 |
| LOC_Os09g26870 | 20.72257 | 23.32635 | 24.18067 | 16.60479 | 16.64107 | 17.60747 | 15.16215 | 16.78891 | 20.52074 |
| LOC_Os09g23300 | 8.121428 | 7.270555 | 5.344957 | 3.292143 | 2.676424 | 2.518025 | 16.99336 | 7.124848 | 11.11935 |
| LOC_Os09g21180 | 2.857533 | 3.797535 | 3.772323 | 0.416165 | 0.37306 | 0.811379 | 0.460866 | 2.502969 | 1.370608 |
| LOC_Os08g39420 | 1.222475 | 2.016291 | 1.717223 | 4.411884 | 3.85435 | 3.690291 | 1.041086 | 1.288102 | 1.3826 |
| LOC_Os08g39100 | 10.61531 | 6.549365 | 9.390416 | 5.952314 | 3.57232 | 4.531049 | 6.018716 | 4.74061 | 7.019565 |
| LOC_Os08g37456 | 3.896801 | 8.469355 | 5.832985 | 14.76736 | 27.25406 | 30.54548 | 5.118902 | 5.931547 | 5.632302 |
| LOC_Os08g20570 | 0.430028 | 0.474775 | 0.565928 | 1.914708 | 1.256829 | 2.182398 | 0.51772 | 0.284552 | 1.178746 |
| LOC_Os08g10510 | 7.151263 | 10.94778 | 8.573467 | 38.8568 | 27.32514 | 21.20011 | 10.54467 | 11.05008 | 14.42447 |
| LOC_Os08g07730 | 2.711276 | 2.25067 | 1.739568 | 1.284552 | 1.358701 | 0.682376 | 0.700955 | 1.63568 | 0.516768 |
| LOC_Os08g06480 | 30.14233 | 32.84707 | 29.08333 | 24.20011 | 23.78006 | 26.12386 | 25.03221 | 29.41762 | 45.29009 |
| LOC_Os08g05660 | 84.90136 | 53.50022 | 86.62833 | 42.33871 | 32.44426 | 47.19001 | 24.54771 | 32.95774 | 26.03628 |
| LOC_Os07g48229 | 7.616515 | 6.425926 | 6.586668 | 4.336363 | 3.412027 | 4.712214 | 3.172676 | 4.166605 | 2.683924 |
| LOC_Os07g47590 | 20.06645 | 13.35811 | 15.14411 | 10.6504 | 7.523606 | 9.282462 | 9.295708 | 11.57143 | 7.240153 |
| LOC_Os07g46410 | 5.276334 | 5.561844 | 6.114295 | 3.288742 | 3.700276 | 3.750339 | 5.074128 | 5.483875 | 6.075008 |
| LOC_Os07g44290 | 8.491478 | 6.415032 | 7.378482 | 3.975688 | 3.028463 | 5.265321 | 2.858619 | 4.245201 | 3.577763 |
| LOC_Os07g42970 | 6.716711 | 6.111936 | 6.089672 | 1.314088 | 1.802238 | 1.792509 | 2.738595 | 3.556446 | 1.53332 |
| LOC_Os07g39010 | 7.794379 | 6.072962 | 5.320922 | 3.14843 | 1.799308 | 1.328715 | 3.831832 | 4.046805 | 2.351331 |
| LOC_Os07g38840 | 5.025292 | 4.852372 | 4.291242 | 2.557717 | 2.285391 | 2.584756 | 3.810592 | 3.189759 | 4.006506 |
| LOC_Os07g33850 | 17.10859 | 15.56361 | 11.55904 | 6.739136 | 7.190235 | 6.475893 | 11.69615 | 14.98347 | 9.371747 |
| LOC_Os07g23730 | 10.26214 | 8.856711 | 12.05314 | 4.307841 | 3.825886 | 7.356299 | 7.56198 | 7.189296 | 3.755467 |
| LOC_Os07g17310 | 70.3644 | 40.24769 | 60.57541 | 16.01991 | 13.54733 | 20.06211 | 52.54562 | 32.19035 | 37.34959 |
| LOC_Os07g08970 | 6.374968 | 7.301833 | 6.726277 | 5.749872 | 3.662965 | 4.253603 | 6.384448 | 6.25044 | 8.686082 |
| LOC_Os07g07560 | 21.09907 | 20.89204 | 21.8023 | 14.84953 | 15.47996 | 16.65813 | 15.1537 | 17.82406 | 19.48791 |
| LOC_Os07g06740 | 1.549923 | 2.028716 | 2.247805 | 4.252517 | 3.873214 | 3.880739 | 1.034316 | 1.187937 | 1.735908 |
| LOC_Os06g47550 | 16.10194 | 13.42463 | 12.57436 | 11.13289 | 10.55848 | 8.860042 | 12.2661 | 10.45702 | 11.74305 |
| LOC_Os06g42850 | 14.06414 | 12.26901 | 12.31378 | 5.711207 | 7.300587 | 9.667077 | 10.54835 | 10.92442 | 12.05604 |
| LOC_Os06g29180 | 46.31197 | 50.04712 | 48.35573 | 108.096 | 92.35503 | 84.18928 | 39.77932 | 38.89001 | 60.1987 |
| LOC_Os06g28880 | 0.522423 | 0.604693 | 0.418482 | 2.09228 | 1.730878 | 1.390509 | 0.444091 | 0.254813 | 0.271122 |
| LOC_Os12g43870 | 16.66034 | 10.53849 | 9.518772 | 9.453524 | 8.670993 | 4.45287 | 17.21724 | 18.40001 | 12.76716 |
| LOC_Os12g43640 | 0.452852 | 0.749862 | 0.434819 | 1.181805 | 1.176751 | 0.869729 | 0.989327 | 1.157824 | 1.169752 |
| LOC_Os12g43600 | 484.2883 | 440.7268 | 639.2478 | 464.9867 | 437.6521 | 673.1805 | 1045.019 | 639.0959 | 1023.196 |
| LOC_Os12g43490 | 0.580667 | 0.814022 | 0.26746 | 1.231699 | 1.014189 | 0.335031 | 1.817516 | 1.658392 | 1.393239 |
| LOC_Os12g43340 | 2.91691 | 4.172962 | 5.03729 | 5.080031 | 5.88672 | 5.112357 | 1.102038 | 1.578469 | 2.187907 |
| LOC_Os12g42420 | 5.100385 | 4.715007 | 4.153567 | 4.55021 | 3.580853 | 4.349749 | 5.6067 | 7.150623 | 7.873781 |
| LOC_Os12g42280 | 1.27342 | 2.312298 | 1.779304 | 3.677069 | 3.01036 | 3.130632 | 7.603535 | 8.18276 | 16.22776 |
| LOC_Os12g41780 | 0.82572 | 0.887799 | 0.767129 | 1.184596 | 1.057746 | 0.869743 | 1.234534 | 1.986765 | 1.25653 |
| LOC_Os12g41670 | 1.020435 | 1.475776 | 1.115812 | 2.899098 | 2.722184 | 2.397845 | 0.730332 | 0.495088 | 1.240546 |
| LOC_Os12g41400 | 5.709175 | 8.08877 | 7.200927 | 9.431208 | 9.179144 | 8.010331 | 2.834176 | 2.740365 | 3.84856 |
| LOC_Os12g40880 | 1.727906 | 2.712638 | 2.529486 | 4.867029 | 3.784392 | 3.590847 | 1.135697 | 0.901688 | 1.576125 |
| LOC_Os12g40500 | 1.284426 | 1.457785 | 1.345517 | 2.028557 | 2.536779 | 1.797093 | 0.452576 | 0.644957 | 0.53086 |
| LOC_Os12g40490 | 4.667264 | 5.336353 | 5.850042 | 5.028463 | 5.365285 | 5.566645 | 6.640487 | 7.408175 | 7.81751 |
| LOC_Os12g39830 | 0.666792 | 0.855685 | 0.960776 | 1.161086 | 1.378717 | 1.194927 | 0.218639 | 0.058094 | 0.322816 |
| LOC_Os12g39640 | 0.847797 | 0.934215 | 0.873233 | 0.813722 | 1.496612 | 2.752216 | 2.744152 | 2.869858 | 4.813914 |
| LOC_Os12g39160 | 1.12901 | 1.81762 | 1.329877 | 3.331029 | 2.724662 | 2.648303 | 0.55812 | 0.445438 | 1.416972 |
| LOC_Os12g38620 | 2.699143 | 2.57446 | 2.992394 | 3.694132 | 2.635861 | 2.858377 | 3.868601 | 3.751141 | 4.918174 |
| LOC_Os12g38490 | 5.605547 | 5.664397 | 5.557209 | 7.476682 | 7.012786 | 7.990743 | 7.44137 | 7.726805 | 9.965201 |
| LOC_Os12g38180 | 2.373767 | 3.162639 | 2.680211 | 3.862142 | 3.556666 | 2.160585 | 1.062296 | 1.121035 | 1.686095 |
| LOC_Os12g38140 | 11.20449 | 9.817054 | 9.713805 | 18.60132 | 16.32946 | 14.73005 | 2.952487 | 1.896027 | 4.754959 |
| LOC_Os12g37840 | 0.847502 | 1.447689 | 1.686001 | 2.747416 | 1.914112 | 1.975562 | 0.696009 | 0.632284 | 0.882686 |
| LOC_Os12g37260 | 0.144938 | 0.33277 | 0.252893 | 0.467067 | 0.352896 | 0.369695 | 0.777278 | 0.854603 | 1.211471 |
| LOC_Os12g36670 | 3.854531 | 3.881902 | 3.371176 | 6.464923 | 5.555502 | 5.496349 | 6.347402 | 5.762795 | 8.834443 |
| LOC_Os12g35610 | 1.276315 | 1.791557 | 1.450906 | 2.002012 | 3.106822 | 3.155919 | 3.029252 | 3.00275 | 2.652534 |
| LOC_Os12g34500 | 2.283095 | 2.707438 | 2.766716 | 4.044936 | 3.216424 | 2.729057 | 0.408121 | 0.837007 | 1.010989 |
| LOC_Os12g34460 | 3.016197 | 3.331021 | 3.864048 | 4.142014 | 4.789287 | 3.188256 | 1.138765 | 1.438817 | 1.411641 |
| LOC_Os12g33160 | 0.967714 | 1.109508 | 1.067567 | 2.485896 | 1.764177 | 1.527291 | 0.415312 | 0.495423 | 0.601635 |
| LOC_Os12g33090 | 0.589476 | 0.769128 | 0.666472 | 0.766435 | 0.740845 | 0.75688 | 1.225261 | 1.05908 | 1.267894 |
| LOC_Os12g32630 | 3.671176 | 2.58149 | 1.851497 | 4.176298 | 3.510082 | 2.670248 | 8.849356 | 8.368609 | 9.102638 |
| LOC_Os12g32499 | 0.438 | 0.675804 | 0.563359 | 1.340578 | 1.098699 | 0.762461 | 0.258557 | 0.218955 | 0.288349 |
| LOC_Os12g32374 | 1.777616 | 1.708711 | 2.602138 | 2.489286 | 2.962806 | 2.896791 | 0.493633 | 0.64076 | 0.908852 |
| LOC_Os12g31880 | 1.630304 | 1.752869 | 1.659065 | 2.062335 | 1.828201 | 1.746142 | 2.31485 | 3.817359 | 4.353616 |
| LOC_Os12g29400 | 24.68173 | 18.75526 | 20.43796 | 18.61748 | 15.59251 | 12.35017 | 23.79429 | 29.26631 | 39.1608 |
| LOC_Os12g21710 | 2.515154 | 3.29924 | 2.691076 | 2.783207 | 3.241884 | 2.376919 | 6.005994 | 8.216901 | 9.147704 |
| LOC_Os12g18640 | 2.150765 | 1.098018 | 2.000187 | 1.670348 | 0.807121 | 1.63421 | 2.229764 | 1.707754 | 3.483345 |
| LOC_Os12g18530 | 0.604745 | 1.072445 | 0.759783 | 3.517273 | 2.462001 | 1.673993 | 0.321041 | 0.488848 | 0.666703 |
| LOC_Os12g14140 | 0.978162 | 0.809033 | 1.04259 | 1.428474 | 1.139653 | 1.071158 | 0.191628 | 0.270153 | 0.243413 |
| LOC_Os12g13910 | 3.997311 | 3.793804 | 4.426909 | 8.450168 | 6.275268 | 5.246386 | 14.14501 | 12.04589 | 15.2456 |
| LOC_Os12g13130 | 1.970813 | 3.56636 | 3.161859 | 5.122506 | 4.287781 | 4.101766 | 1.497864 | 1.216658 | 1.800496 |
| LOC_Os12g09300 | 4.653099 | 7.974974 | 6.015856 | 5.280578 | 8.6704 | 6.884073 | 0.900174 | 0.696733 | 0.663332 |
| LOC_Os12g07640 | 4.174226 | 5.765215 | 3.185505 | 3.791177 | 6.140543 | 5.059528 | 0.345742 | 0.467151 | 0.565846 |
| LOC_Os12g06800 | 1.215274 | 1.695683 | 1.65826 | 2.520936 | 1.854948 | 2.371766 | 2.900197 | 2.522656 | 5.159752 |
| LOC_Os12g06630 | 3.713593 | 3.259351 | 3.522839 | 4.913044 | 3.898517 | 4.026072 | 4.695058 | 4.306359 | 5.826838 |
| LOC_Os12g06260 | 2.224497 | 2.108539 | 3.172083 | 2.540378 | 3.032399 | 4.686567 | 0.733424 | 0.827601 | 0.980272 |
| LOC_Os12g03822 | 3.006306 | 3.413803 | 3.140547 | 5.503727 | 4.771927 | 3.299859 | 1.206017 | 1.015742 | 1.315133 |
| LOC_Os12g02530 | 1.814782 | 2.512588 | 2.322377 | 2.726757 | 1.992111 | 2.258492 | 3.204261 | 3.139803 | 7.562471 |
| LOC_Os12g02310 | 527.0429 | 372.0948 | 277.3778 | 294.414 | 365.2566 | 215.1571 | 428.1369 | 500.8195 | 156.9443 |
| LOC_Os12g02200 | 0.646859 | 0.375365 | 1.361259 | 1.13741 | 0.277416 | 0.920639 | 4.800416 | 3.917504 | 5.545416 |
| LOC_Os12g01590 | 1.23784 | 0.872583 | 0.414609 | 1.118355 | 0.855276 | 0.493713 | 1.967199 | 1.381827 | 1.183687 |
| LOC_Os12g01560 | 0.772268 | 0.67699 | 0.446839 | 0.643169 | 1.083477 | 0.581397 | 0.129394 | 0.04262 | 0.080406 |
| LOC_Os12g01530 | 6.071286 | 6.878538 | 7.103613 | 5.115296 | 4.730601 | 7.511148 | 14.20507 | 23.50486 | 23.06132 |
| LOC_Os11g44800 | 1.499409 | 1.346752 | 1.060433 | 1.653491 | 1.582916 | 0.944295 | 0.254877 | 0.258958 | 0.176921 |
| LOC_Os11g42170 | 11.99654 | 20.00477 | 12.85608 | 2.852664 | 5.696757 | 4.040214 | 6.832415 | 21.66559 | 5.882235 |
| LOC_Os11g41860 | 2.00257 | 2.183099 | 2.932289 | 1.484193 | 2.010262 | 1.717571 | 0.348907 | 0.26985 | 0.493109 |
| LOC_Os11g41410 | 1.990807 | 2.657773 | 2.555901 | 6.474083 | 6.052046 | 4.254775 | 0.777454 | 0.548805 | 0.8159 |
| LOC_Os11g40200 | 2.246548 | 2.273568 | 2.39413 | 3.324047 | 2.408921 | 1.970554 | 0.880476 | 0.677457 | 1.146187 |
| LOC_Os11g40070 | 2.634255 | 2.922981 | 3.564986 | 4.779882 | 3.392699 | 3.493804 | 4.631529 | 5.133748 | 6.531893 |
| LOC_Os11g39650 | 9.433541 | 9.854686 | 8.458979 | 12.09247 | 11.88188 | 11.29195 | 14.55458 | 17.10411 | 19.15171 |
| LOC_Os11g37670 | 0.580493 | 0.438826 | 0.607704 | 0.895442 | 0.591539 | 0.70167 | 1.348884 | 0.993847 | 1.567011 |
| LOC_Os11g37640 | 17.55823 | 17.88623 | 20.22445 | 15.52487 | 17.01812 | 18.86889 | 19.5659 | 24.36505 | 28.44658 |
| LOC_Os11g37260 | 8.47078 | 6.732951 | 6.07237 | 6.838032 | 5.057798 | 4.173935 | 6.724339 | 8.887372 | 7.912241 |
| LOC_Os11g35710 | 1.900913 | 2.481701 | 1.681966 | 5.5259 | 4.644935 | 2.793511 | 0.602171 | 1.094612 | 0.837334 |
| LOC_Os11g34910 | 1.187865 | 1.164431 | 1.01341 | 2.125271 | 1.700571 | 1.264922 | 0.260046 | 0.559346 | 0.302772 |
| LOC_Os11g34824 | 4.625866 | 4.689984 | 5.475254 | 7.039329 | 6.069167 | 6.128126 | 1.462717 | 1.262065 | 2.571787 |
| LOC_Os11g34080 | 10.00756 | 14.04109 | 13.8924 | 11.5772 | 20.99824 | 21.98304 | 26.25329 | 26.68928 | 26.22576 |
| LOC_Os11g33100 | 4.023244 | 3.280801 | 3.354036 | 2.675318 | 2.715728 | 2.377731 | 4.101254 | 5.063828 | 5.932662 |
| LOC_Os11g32880 | 0.467213 | 0.412998 | 0.318238 | 0.931541 | 0.644176 | 0.626311 | 1.293332 | 1.449143 | 1.626068 |
| LOC_Os11g24630 | 0.725973 | 0.528491 | 1.01479 | 1.260948 | 1.05072 | 0.552607 | 1.993936 | 1.482899 | 1.828316 |
| LOC_Os11g20750 | 1.234116 | 1.480925 | 1.376473 | 1.844649 | 1.794641 | 1.391016 | 0.269112 | 0.507056 | 0.497719 |
| LOC_Os11g18830 | 1.26353 | 1.705424 | 1.790939 | 3.367421 | 2.922044 | 1.994926 | 0.418007 | 0.523638 | 0.710144 |
| LOC_Os11g15000 | 6.541557 | 7.044721 | 6.808257 | 10.86474 | 9.471664 | 9.7243 | 2.243712 | 3.68529 | 3.959888 |
| LOC_Os11g13890 | 11.83049 | 27.7949 | 16.31407 | 44.41119 | 28.84042 | 26.41066 | 10.25023 | 10.06869 | 18.72984 |
| LOC_Os11g13670 | 1.282173 | 3.615896 | 1.547045 | 4.412424 | 5.244162 | 3.038054 | 1.352836 | 1.808729 | 1.978695 |
| LOC_Os11g11100 | 4.993771 | 5.216308 | 4.147955 | 5.42759 | 6.41134 | 5.346874 | 6.508972 | 8.496334 | 6.682531 |
| LOC_Os11g11020 | 4.145943 | 4.356615 | 3.338269 | 9.126816 | 7.341045 | 4.26631 | 1.177284 | 1.425932 | 1.434754 |
| LOC_Os11g10510 | 10.31676 | 11.60957 | 14.17932 | 5.033722 | 11.68324 | 17.16073 | 4.815345 | 4.917324 | 6.899953 |
| LOC_Os11g09280 | 244.5271 | 141.4682 | 242.8546 | 223.2152 | 189.3114 | 310.2999 | 381.9631 | 179.7371 | 427.5045 |
| LOC_Os11g09140 | 2.219012 | 2.513019 | 1.46531 | 3.117894 | 2.978467 | 2.100702 | 3.361532 | 3.685331 | 3.353057 |
| LOC_Os11g09010 | 3.050708 | 3.502786 | 2.87501 | 4.276004 | 3.437517 | 4.098807 | 5.211299 | 5.366646 | 6.54784 |
| LOC_Os11g08210 | 3.885737 | 3.619171 | 4.316256 | 3.883775 | 3.453952 | 3.606456 | 21.62894 | 18.53461 | 16.40167 |
| LOC_Os11g07600 | 14.3759 | 10.66901 | 11.11398 | 10.72113 | 8.946957 | 10.78582 | 24.41734 | 25.91174 | 27.05845 |
| LOC_Os11g07440 | 0.544551 | 0.611144 | 0.871759 | 1.937016 | 1.142182 | 0.849791 | 2.312507 | 1.465884 | 3.007822 |
| LOC_Os11g06170 | 9.830902 | 7.972253 | 9.428978 | 8.872176 | 10.24987 | 13.82872 | 16.6422 | 18.05065 | 23.69708 |
| LOC_Os11g05930 | 3.892546 | 7.030822 | 4.697603 | 5.62517 | 7.71856 | 4.917715 | 5.95326 | 10.26998 | 7.400253 |
| LOC_Os11g05760 | 2.439211 | 5.706826 | 5.672309 | 7.519011 | 7.829706 | 5.709046 | 1.753347 | 1.799078 | 1.833091 |
| LOC_Os11g05640 | 5.345467 | 4.631855 | 5.816353 | 7.600049 | 5.22972 | 5.010369 | 13.42832 | 17.35731 | 21.01581 |
| LOC_Os11g05540 | 1.178192 | 1.294528 | 1.155326 | 2.397873 | 1.746035 | 1.301573 | 0.31387 | 0.37224 | 0.402715 |
| LOC_Os11g05170 | 81.92951 | 62.34189 | 58.27488 | 90.72902 | 73.84359 | 33.9876 | 8.469886 | 13.38257 | 8.847569 |
| LOC_Os11g05140 | 1.616349 | 1.702675 | 1.662804 | 2.580373 | 2.340202 | 2.022395 | 0.405639 | 0.699336 | 0.610829 |
| LOC_Os11g03990 | 2.494738 | 4.826028 | 4.78586 | 5.777491 | 5.751087 | 4.806548 | 1.312956 | 1.817755 | 2.770753 |
| LOC_Os11g03550 | 0.861887 | 0.852094 | 0.614813 | 0.91258 | 0.839374 | 0.936236 | 1.406852 | 1.385192 | 1.706305 |
| LOC_Os11g03540 | 0.362717 | 0.979414 | 0.685397 | 1.879093 | 1.558414 | 1.446631 | 1.606003 | 2.28295 | 2.27944 |
| LOC_Os11g03300 | 0.458965 | 0.817203 | 1.054248 | 2.49556 | 0.577576 | 0.683307 | 2.551678 | 1.860161 | 2.447692 |
| LOC_Os11g02630 | 0.412183 | 1.326416 | 0.709633 | 1.395661 | 2.319539 | 1.597304 | 0.856064 | 0.54276 | 0.553871 |
| LOC_Os11g02464 | 5.967377 | 8.842413 | 8.180349 | 14.27873 | 14.00752 | 13.44848 | 3.598105 | 3.547263 | 6.908704 |
| LOC_Os11g02389 | 899.472 | 806.0043 | 726.351 | 602.4511 | 798.5503 | 445.4026 | 954.0176 | 1191.142 | 476.3718 |
| LOC_Os11g02240 | 0.095266 | 0.118534 | 0.188592 | 0.358409 | 0.125234 | 0.277365 | 2.609857 | 2.891826 | 3.142311 |
| LOC_Os11g02090 | 3.391288 | 3.642414 | 3.620301 | 4.373486 | 3.816834 | 2.963791 | 1.398484 | 1.614024 | 1.525427 |
| LOC_Os11g02070 | 2.629695 | 1.922851 | 2.09195 | 0.891592 | 1.072135 | 2.383683 | 1.392352 | 2.68986 | 0.760113 |
| LOC_Os11g01570 | 1.046627 | 1.347993 | 1.557158 | 2.409176 | 1.738559 | 1.717919 | 0.109653 | 0.148591 | 0.229898 |
| LOC_Os11g01530 | 3.815097 | 4.761924 | 4.860991 | 3.428537 | 3.47625 | 5.525566 | 10.84228 | 15.00653 | 16.94648 |
| LOC_Os10g41960 | 19.546 | 25.17151 | 23.20605 | 31.67336 | 29.82353 | 26.69857 | 9.059129 | 9.956311 | 16.41547 |
| LOC_Os10g41930 | 0.802178 | 1.256827 | 0.887819 | 0.724097 | 0.659419 | 0.571921 | 1.490709 | 2.550304 | 5.314562 |
| LOC_Os10g41760 | 0.788993 | 0.89172 | 0.452101 | 0.78889 | 0.80797 | 0.593044 | 1.514248 | 1.850814 | 1.197254 |
| LOC_Os10g41650 | 2.846715 | 3.536221 | 3.198764 | 4.128743 | 3.976784 | 3.711149 | 4.325263 | 5.28173 | 4.835488 |
| LOC_Os10g41250 | 17.71946 | 12.70544 | 12.95815 | 6.643359 | 6.47137 | 7.011893 | 12.31544 | 16.09764 | 9.169012 |
| LOC_Os10g41230 | 2.48781 | 3.159626 | 3.150951 | 2.90272 | 3.812087 | 3.235548 | 10.0488 | 5.575394 | 4.572609 |
| LOC_Os10g40934 | 0.752565 | 1.34509 | 1.622387 | 1.443757 | 1.476987 | 0.933687 | 0.260158 | 0.460588 | 0.315493 |
| LOC_Os10g40660 | 0.039674 | 0.323924 | 0.256714 | 0.524491 | 0.871987 | 0.904778 | 0.00765 | 0.0185 | 0.104585 |
| LOC_Os10g40640 | 1.142124 | 1.119106 | 1.25758 | 1.660839 | 1.528359 | 1.511653 | 2.99899 | 3.422515 | 4.462768 |
| LOC_Os10g40550 | 3.707776 | 3.296658 | 4.789197 | 1.946713 | 1.675809 | 3.164466 | 3.837244 | 5.700013 | 4.980829 |
| LOC_Os10g39980 | 0.649828 | 0.42368 | 0.868528 | 0.745365 | 0.44756 | 0.863033 | 1.662569 | 1.720833 | 3.291046 |
| LOC_Os10g39930 | 1.346945 | 1.714906 | 0.978988 | 1.798306 | 2.318384 | 2.106542 | 0.433607 | 0.747419 | 0.876531 |
| LOC_Os10g39640 | 6.548541 | 6.683762 | 8.781641 | 9.666397 | 8.119907 | 8.379171 | 13.09242 | 13.47885 | 18.4725 |
| LOC_Os10g38740 | 5.389794 | 4.50011 | 3.529946 | 3.192104 | 4.5876 | 2.630023 | 6.687559 | 8.228981 | 5.976646 |
| LOC_Os10g38030 | 3.091715 | 2.865147 | 3.662775 | 4.147471 | 3.451537 | 4.177083 | 4.481919 | 4.451126 | 8.070575 |
| LOC_Os10g36980 | 1.803191 | 1.270556 | 0.665806 | 1.119297 | 1.419001 | 0.812567 | 1.682162 | 2.105108 | 1.265441 |
| LOC_Os10g36848 | 2.854121 | 5.921787 | 5.672544 | 10.52709 | 12.91391 | 9.270886 | 2.628401 | 1.871252 | 2.12327 |
| LOC_Os10g36703 | 11.12474 | 11.52698 | 11.92937 | 17.93909 | 12.77551 | 7.928415 | 23.64779 | 20.29046 | 30.3593 |
| LOC_Os10g36180 | 58.55342 | 38.7564 | 37.33 | 37.0692 | 27.24426 | 22.83328 | 55.83745 | 76.04943 | 67.8468 |
| LOC_Os10g35710 | 8.098502 | 8.158503 | 6.228953 | 6.919296 | 8.030071 | 6.125715 | 9.037337 | 11.523 | 11.95672 |
| LOC_Os10g35630 | 4.129068 | 4.938191 | 4.624783 | 8.673439 | 9.154368 | 9.493936 | 18.41302 | 14.19189 | 21.0558 |
| LOC_Os10g35550 | 5.731146 | 5.930882 | 5.587117 | 5.920091 | 5.509853 | 5.193674 | 7.414428 | 10.11916 | 13.94181 |
| LOC_Os10g35370 | 1.174385 | 2.453587 | 2.203986 | 4.848601 | 4.41067 | 4.42649 | 0.639851 | 0.599167 | 2.700566 |
| LOC_Os10g35180 | 0.189593 | 0.248338 | 0.181619 | 0.463556 | 0.541977 | 0.592174 | 0.882493 | 0.896764 | 0.720051 |
| LOC_Os10g34420 | 1.255057 | 1.255939 | 1.376821 | 1.477364 | 1.095355 | 1.356884 | 1.88727 | 2.260659 | 2.47096 |
| LOC_Os10g33310 | 2.607017 | 3.23588 | 2.777617 | 5.089652 | 4.120073 | 5.068748 | 6.501319 | 6.573027 | 8.014988 |
| LOC_Os10g33240 | 0.119006 | 0.171277 | 0.138168 | 0.093522 | 0.133623 | 0.088737 | 0.487107 | 0.486966 | 0.564664 |
| LOC_Os10g32770 | 1.843585 | 2.758451 | 1.985334 | 4.764765 | 4.381684 | 3.016802 | 0.905623 | 1.39825 | 1.147111 |
| LOC_Os10g32550 | 17.947 | 20.21992 | 17.14556 | 40.60352 | 31.41786 | 21.24314 | 5.66748 | 6.118872 | 6.354533 |
| LOC_Os10g30150 | 36.172 | 15.45904 | 20.7055 | 28.45955 | 19.71196 | 22.29272 | 64.05464 | 49.01931 | 45.76153 |
| LOC_Os10g30054 | 2.010865 | 2.111753 | 1.655554 | 2.318499 | 2.656687 | 1.571252 | 3.725676 | 3.069361 | 4.165095 |
| LOC_Os10g29660 | 8.526859 | 10.09589 | 8.800518 | 7.575441 | 9.114267 | 6.567675 | 13.63128 | 13.90128 | 15.6288 |
| LOC_Os10g28360 | 3.417477 | 4.769986 | 4.690878 | 5.304177 | 4.476833 | 4.063784 | 0.616911 | 0.941796 | 1.141332 |
| LOC_Os10g28200 | 4.573389 | 4.075936 | 5.001567 | 6.13565 | 3.521116 | 4.362321 | 9.263023 | 8.509576 | 13.42667 |
| LOC_Os10g28000 | 2.066066 | 1.876459 | 1.823657 | 1.419429 | 1.454108 | 0.895525 | 2.558438 | 4.19454 | 2.838228 |
| LOC_Os10g27000 | 1.947244 | 1.656017 | 1.936935 | 2.298668 | 1.511398 | 1.481726 | 0.551475 | 0.637151 | 0.801758 |
| LOC_Os10g26620 | 2.050903 | 1.856177 | 1.728822 | 1.077202 | 1.358199 | 1.145462 | 3.975655 | 5.27943 | 6.353551 |
| LOC_Os10g26570 | 0.349295 | 0.400469 | 0.261628 | 0.554403 | 0.713 | 0.439798 | 0.773595 | 1.155943 | 1.102576 |
| LOC_Os10g26560 | 0.133891 | 0.198734 | 0.162273 | 0.237628 | 0.387885 | 0.173169 | 0.376998 | 0.505152 | 0.501986 |
| LOC_Os10g26150 | 2.149411 | 9.745553 | 6.810467 | 12.83966 | 23.58784 | 10.35458 | 2.216036 | 3.542823 | 2.707363 |
| LOC_Os10g25830 | 0.765228 | 0.741274 | 0.977765 | 2.443415 | 0.913287 | 1.720947 | 1.640631 | 1.163477 | 2.198061 |
| LOC_Os10g25487 | 0.515508 | 1.14421 | 0.74927 | 1.640047 | 1.64453 | 0.903888 | 0.231681 | 0.479607 | 0.241812 |
| LOC_Os10g25310 | 9.353529 | 6.002587 | 9.023982 | 8.207743 | 7.215606 | 7.068631 | 31.70295 | 28.83664 | 35.04398 |
| LOC_Os10g25290 | 10.14465 | 4.56995 | 9.146527 | 8.474726 | 3.740315 | 6.417116 | 12.15338 | 5.611871 | 13.03983 |
| LOC_Os10g25140 | 2.636406 | 4.166389 | 3.636639 | 7.263193 | 6.564842 | 5.349971 | 1.492596 | 1.791629 | 1.871467 |
| LOC_Os10g21660 | 0.428037 | 0.483421 | 0.395621 | 1.221671 | 0.72251 | 0.714189 | 0.093018 | 0.106791 | 0.144288 |
| LOC_Os10g20470 | 4.152417 | 2.732049 | 3.267019 | 4.536989 | 2.681118 | 2.702241 | 5.441299 | 5.443365 | 4.316838 |
| LOC_Os10g17260 | 0.683229 | 1.106537 | 0.513288 | 0.332708 | 0.862664 | 0.345087 | 1.316478 | 1.464485 | 1.123955 |
| LOC_Os10g12390 | 4.548176 | 11.55323 | 10.77765 | 8.753553 | 11.86366 | 10.03826 | 0.783247 | 1.930227 | 2.168471 |
| LOC_Os10g12354 | 0.646842 | 0.606844 | 0.451357 | 1.160657 | 0.88495 | 0.84475 | 1.830483 | 1.259751 | 1.695236 |
| LOC_Os10g11810 | 7.11134 | 8.744599 | 9.889405 | 10.9888 | 11.09786 | 10.72085 | 3.105537 | 3.531397 | 5.992058 |
| LOC_Os10g09240 | 3.58102 | 1.919244 | 2.391429 | 2.387345 | 2.074126 | 0.693606 | 1.01342 | 10.81544 | 1.687801 |
| LOC_Os10g04700 | 0.995741 | 1.461489 | 1.086894 | 2.929201 | 2.445946 | 1.717873 | 0.504775 | 0.423638 | 0.432576 |
| LOC_Os10g04130 | 1.397601 | 1.591649 | 1.483039 | 2.747665 | 2.397522 | 1.990438 | 0.425898 | 0.497687 | 0.790642 |
| LOC_Os10g01480 | 7.946974 | 10.77521 | 7.688306 | 13.20938 | 18.1968 | 15.28262 | 25.1165 | 26.64916 | 33.55347 |
| LOC_Os10g01470 | 1.66124 | 1.880299 | 2.037194 | 3.917858 | 3.074345 | 2.203206 | 4.530734 | 5.553695 | 6.844263 |
| LOC_Os10g01134 | 15.77102 | 16.43354 | 14.44687 | 18.73617 | 26.12031 | 20.73131 | 6.521381 | 8.148365 | 6.844989 |
| LOC_Os10g01100 | 0.426469 | 0.796662 | 0.497449 | 0.8949 | 1.142259 | 0.663099 | 1.075175 | 1.23682 | 1.190036 |
| LOC_Os09g39760 | 0.183776 | 0.30107 | 0.407814 | 0.486249 | 0.555709 | 0.265995 | 0.799546 | 1.149661 | 1.488398 |
| LOC_Os09g39310 | 1.88622 | 2.282439 | 1.980493 | 3.29128 | 2.898119 | 2.796312 | 0.803068 | 0.811917 | 1.201977 |
| LOC_Os09g38570 | 1.84837 | 2.420924 | 2.43708 | 2.43375 | 2.703592 | 2.871479 | 4.075304 | 4.063068 | 5.937419 |
| LOC_Os09g38110 | 0.812326 | 0.555727 | 1.155584 | 1.820673 | 1.598766 | 2.045747 | 0.029967 | 0.049495 | 0.131142 |
| LOC_Os09g37006 | 5.881186 | 6.780165 | 6.290469 | 9.8668 | 8.041555 | 6.571154 | 2.231513 | 2.922419 | 3.215513 |
| LOC_Os09g36790 | 14.20725 | 11.02582 | 15.68516 | 5.308404 | 5.355281 | 9.595602 | 14.21124 | 14.41288 | 11.46978 |
| LOC_Os09g36470 | 0.919311 | 1.383381 | 1.024175 | 2.693526 | 2.208513 | 2.458188 | 0.27617 | 0.321097 | 0.576529 |
| LOC_Os09g36290 | 5.111811 | 6.092319 | 4.47151 | 7.864776 | 6.644667 | 5.198018 | 10.4559 | 11.6588 | 11.82121 |
| LOC_Os09g36250 | 3.049647 | 2.068284 | 2.641055 | 3.094218 | 2.819609 | 2.804609 | 0.952726 | 1.511702 | 1.353807 |
| LOC_Os09g35940 | 1.229055 | 0.616721 | 0.67956 | 1.491094 | 0.686825 | 1.347238 | 8.160096 | 3.811096 | 4.45086 |
| LOC_Os09g35800 | 11.11071 | 12.3275 | 13.66479 | 14.85738 | 15.17636 | 12.19205 | 25.58477 | 31.85031 | 39.12897 |
| LOC_Os09g35790 | 34.21213 | 21.76042 | 26.47032 | 20.97276 | 25.06625 | 18.5714 | 6.580404 | 10.36537 | 3.495536 |
| LOC_Os09g34010 | 2.021269 | 2.213296 | 2.313103 | 3.147551 | 2.626464 | 2.314878 | 0.816839 | 0.734416 | 1.261177 |
| LOC_Os09g33550 | 0.504262 | 0.552203 | 0.618476 | 1.010605 | 1.317788 | 1.821404 | 1.551492 | 1.896813 | 1.763988 |
| LOC_Os09g33520 | 4.914866 | 5.2373 | 4.960782 | 5.933228 | 5.533571 | 5.878405 | 1.589428 | 1.646969 | 1.710191 |
| LOC_Os09g32270 | 0.770318 | 0.966465 | 1.190959 | 1.664719 | 1.052271 | 1.102213 | 0.243418 | 0.325166 | 0.457795 |
| LOC_Os09g32030 | 1.425085 | 1.11517 | 1.583336 | 1.587387 | 1.336083 | 1.229481 | 6.088462 | 5.854593 | 6.328288 |
| LOC_Os09g32010 | 1.850051 | 1.603988 | 1.80345 | 1.687749 | 1.58156 | 1.648212 | 2.508677 | 3.316305 | 3.59307 |
| LOC_Os09g31482 | 30.10797 | 30.84697 | 28.06234 | 44.47833 | 36.1414 | 25.59386 | 7.167465 | 11.1225 | 7.589853 |
| LOC_Os09g31270 | 1.031347 | 1.369343 | 1.358481 | 3.105844 | 1.955116 | 2.102557 | 0.38295 | 0.348036 | 0.496854 |
| LOC_Os09g31190 | 0.854449 | 0.477067 | 0.385382 | 0.218377 | 0.252523 | 0.283488 | 3.92967 | 2.026798 | 2.914407 |
| LOC_Os09g31120 | 3.802816 | 3.131988 | 5.806351 | 5.770164 | 4.302407 | 3.533497 | 1.369309 | 1.480273 | 2.232639 |
| LOC_Os09g30400 | 1.781711 | 1.705813 | 1.66403 | 2.051341 | 1.656809 | 1.764232 | 3.259116 | 4.269832 | 4.06709 |
| LOC_Os09g30120 | 0.556983 | 1.202806 | 0.951714 | 1.705956 | 1.758688 | 1.42736 | 0.346605 | 0.49311 | 0.520841 |
| LOC_Os09g29390 | 0.573463 | 0.421059 | 0.826624 | 1.561134 | 0.731919 | 0.852437 | 0.072459 | 0.029037 | 0.049609 |
| LOC_Os09g28390 | 0.191724 | 0.370379 | 0.45624 | 0.411791 | 0.296172 | 0.278714 | 0.946797 | 1.614537 | 1.938512 |
| LOC_Os09g28000 | 6.531924 | 6.305339 | 5.69424 | 4.655964 | 5.310288 | 4.941994 | 6.566384 | 8.242872 | 9.22729 |
| LOC_Os09g27980 | 8.276545 | 5.900205 | 6.462284 | 4.059638 | 4.91014 | 3.815906 | 10.7838 | 14.72255 | 12.05529 |
| LOC_Os09g27330 | 0.857728 | 0.360211 | 0.29184 | 0.592231 | 0.500456 | 0.468687 | 5.317943 | 6.427615 | 5.035553 |
| LOC_Os09g27010 | 1.820855 | 1.013208 | 1.969845 | 1.510432 | 0.987813 | 1.324717 | 2.624787 | 3.036684 | 2.610728 |
| LOC_Os09g26920 | 0.267326 | 0.210653 | 0.220873 | 0.220113 | 0.267892 | 0.346497 | 1.081081 | 1.536948 | 0.933235 |
| LOC_Os09g26730 | 14.69212 | 18.92532 | 17.40274 | 34.60291 | 33.85806 | 27.34883 | 11.43559 | 6.604488 | 14.52775 |
| LOC_Os09g26004 | 1.531224 | 1.891538 | 2.028523 | 2.914606 | 2.27216 | 2.598524 | 0.603225 | 0.812004 | 1.271223 |
| LOC_Os09g25410 | 1.321537 | 1.71449 | 1.625566 | 1.757432 | 1.66851 | 1.264243 | 2.448797 | 2.080151 | 2.956069 |
| LOC_Os09g24490 | 0.254115 | 0.704977 | 0.431257 | 0.306291 | 0.611729 | 0.38738 | 0.908372 | 2.116127 | 1.160487 |
| LOC_Os09g24330 | 1.424192 | 2.252596 | 1.691505 | 2.336823 | 3.176714 | 2.205252 | 3.860149 | 4.314669 | 4.32391 |
| LOC_Os09g23350 | 2.140039 | 1.779862 | 1.787524 | 3.192552 | 2.034622 | 2.757503 | 4.438756 | 4.17786 | 4.436329 |
| LOC_Os09g23220 | 0.201627 | 0.602285 | 0.172219 | 1.077516 | 1.011803 | 0.710303 | 0.106167 | 0.101232 | 0.170238 |
| LOC_Os09g22440 | 15.66698 | 15.1928 | 15.26595 | 15.97099 | 13.99431 | 13.48745 | 19.77039 | 20.48443 | 22.21571 |
| LOC_Os09g21230 | 3.311623 | 3.584132 | 3.303664 | 6.500762 | 6.068785 | 4.594325 | 6.745255 | 6.099573 | 7.822268 |
| LOC_Os09g21120 | 0.70655 | 0.794792 | 0.703971 | 0.827792 | 0.99676 | 0.531737 | 2.856223 | 2.913887 | 3.305726 |
| LOC_Os09g20480 | 0.425429 | 0.446077 | 0.540665 | 0.752185 | 0.441299 | 0.657786 | 1.477564 | 1.440454 | 1.617139 |
| LOC_Os09g20350 | 21.66721 | 23.58288 | 17.73486 | 29.88084 | 28.16283 | 25.52299 | 32.88446 | 34.01374 | 36.47325 |
| LOC_Os09g18320 | 5.400871 | 6.053713 | 6.250455 | 8.564677 | 7.261703 | 5.581377 | 2.591765 | 2.415107 | 2.965958 |
| LOC_Os09g17740 | 41.73177 | 93.90243 | 67.30023 | 123.3976 | 125.2296 | 100.2175 | 15.97507 | 16.24241 | 43.74781 |
| LOC_Os09g15820 | 4.678989 | 3.460931 | 5.242158 | 5.443117 | 3.598643 | 4.304215 | 7.576072 | 6.322769 | 10.79536 |
| LOC_Os09g15810 | 4.998682 | 6.153378 | 6.418078 | 5.939476 | 8.564516 | 8.735608 | 9.443801 | 11.26243 | 16.69143 |
| LOC_Os09g15780 | 2.008002 | 1.853759 | 2.854498 | 0.911616 | 1.526134 | 1.423044 | 0.387147 | 0.283741 | 0.29771 |
| LOC_Os09g15770 | 42.46457 | 43.56756 | 43.12977 | 52.06473 | 43.08313 | 40.25523 | 10.00103 | 14.10096 | 19.95142 |
| LOC_Os09g15670 | 13.72686 | 10.76515 | 11.88577 | 11.9408 | 10.67694 | 8.070345 | 45.58072 | 50.65453 | 40.699 |
| LOC_Os09g15420 | 2.723747 | 3.02538 | 4.49466 | 3.157735 | 2.618074 | 3.675823 | 4.966468 | 6.100471 | 7.83649 |
| LOC_Os09g13940 | 1.163349 | 1.689567 | 1.444157 | 3.667951 | 2.742007 | 2.554007 | 2.655809 | 3.36061 | 4.167528 |
| LOC_Os09g13820 | 0.247883 | 0.353028 | 0.274093 | 0.864447 | 0.602142 | 0.560739 | 0.080113 | 0.090423 | 0.07894 |
| LOC_Os09g12590 | 3.569381 | 6.505519 | 2.622928 | 6.974308 | 9.150987 | 4.005866 | 1.436359 | 1.874374 | 0.974283 |
| LOC_Os09g11510 | 2.059461 | 2.317507 | 1.31042 | 3.259612 | 2.683515 | 1.537484 | 0.648336 | 0.5894 | 0.73684 |
| LOC_Os09g11460 | 3.022275 | 5.034118 | 5.125947 | 6.619055 | 6.547407 | 6.837545 | 0.635363 | 0.694534 | 0.642704 |
| LOC_Os09g10910 | 2.365039 | 2.773465 | 2.755349 | 5.490178 | 5.10796 | 5.235943 | 1.782071 | 1.243682 | 1.702235 |
| LOC_Os09g10780 | 3.31127 | 2.238777 | 2.778429 | 3.607189 | 3.855997 | 3.349765 | 9.519893 | 9.810768 | 13.29545 |
| LOC_Os09g10300 | 6.003453 | 4.466262 | 5.427022 | 1.655427 | 2.905701 | 2.426006 | 4.302693 | 5.278365 | 5.651312 |
| LOC_Os09g09980 | 1.302034 | 1.389702 | 1.218651 | 2.326127 | 2.676272 | 1.824996 | 0.305101 | 0.395518 | 0.573162 |
| LOC_Os09g08660 | 2.428293 | 2.401411 | 2.763537 | 2.431478 | 2.621719 | 2.24497 | 5.583068 | 7.59195 | 8.109725 |
| LOC_Os09g06970 | 1.293383 | 2.29483 | 1.690471 | 3.8253 | 2.569437 | 2.517576 | 0.836358 | 0.981252 | 1.039685 |
| LOC_Os09g06910 | 1.142986 | 1.990569 | 1.177209 | 2.185683 | 2.742595 | 1.627533 | 0.480909 | 0.688998 | 0.56568 |
| LOC_Os09g05020 | 1.32606 | 1.766091 | 1.945564 | 1.993224 | 1.912651 | 2.199263 | 2.62151 | 3.730223 | 4.864154 |
| LOC_Os09g04504 | 4.022973 | 5.222489 | 5.697629 | 11.39136 | 7.873749 | 9.323954 | 1.693609 | 1.322011 | 2.342392 |
| LOC_Os09g04380 | 1.077879 | 1.179125 | 1.332013 | 1.898043 | 1.885922 | 1.572825 | 0.355347 | 0.237967 | 0.374475 |
| LOC_Os09g04290 | 9.300143 | 9.698595 | 11.58529 | 14.64388 | 15.49102 | 20.90858 | 4.227252 | 5.154259 | 7.902499 |
| LOC_Os09g04050 | 1.502662 | 1.184448 | 1.56574 | 1.111415 | 1.622861 | 1.845703 | 0.176883 | 0.204977 | 0.105151 |
| LOC_Os08g44930 | 2.176481 | 2.228611 | 3.351461 | 3.165064 | 2.498168 | 2.921982 | 0.584528 | 0.451811 | 1.1009 |
| LOC_Os08g44850 | 8.11718 | 8.726227 | 1.797703 | 14.52901 | 13.54732 | 2.647289 | 3.383703 | 5.256582 | 5.631523 |
| LOC_Os08g44680 | 7.041221 | 12.66244 | 10.20861 | 24.37735 | 17.29878 | 18.40227 | 4.996331 | 6.085236 | 19.21136 |
| LOC_Os08g44190 | 4.629748 | 5.411747 | 5.228479 | 5.326828 | 5.339613 | 4.987388 | 0.829938 | 1.270721 | 1.317707 |
| LOC_Os08g44180 | 1.231352 | 1.499636 | 1.183559 | 2.867199 | 1.990339 | 1.265425 | 0.523161 | 0.331949 | 0.334248 |
| LOC_Os08g43630 | 0.350024 | 0.781279 | 0.562243 | 0.512237 | 0.565897 | 0.271599 | 1.225837 | 2.101016 | 1.524569 |
| LOC_Os08g43160 | 1.985906 | 2.08305 | 2.663108 | 3.294019 | 2.930724 | 2.705311 | 4.529891 | 4.342679 | 7.620148 |
| LOC_Os08g41750 | 5.417304 | 3.477371 | 4.553428 | 3.523933 | 3.943696 | 3.043985 | 13.10215 | 12.97968 | 11.57376 |
| LOC_Os08g41720 | 7.117897 | 4.343212 | 5.427106 | 5.038907 | 3.364701 | 7.8268 | 1.505673 | 1.039821 | 1.106554 |
| LOC_Os08g41390 | 14.72874 | 17.52233 | 17.66547 | 27.97371 | 25.82223 | 19.34086 | 5.975305 | 7.399152 | 8.91209 |
| LOC_Os08g40620 | 16.92929 | 10.67391 | 10.43617 | 8.591572 | 7.515572 | 7.774179 | 14.35483 | 13.80946 | 13.91787 |
| LOC_Os08g39550 | 2.43916 | 2.82464 | 1.902161 | 3.471162 | 4.114477 | 3.458285 | 1.031937 | 0.939749 | 1.465241 |
| LOC_Os08g39500 | 13.37139 | 11.22169 | 13.89384 | 18.74041 | 15.5816 | 14.95764 | 5.190908 | 6.311988 | 7.514475 |
| LOC_Os08g39310 | 4.589138 | 4.176142 | 3.795508 | 4.827944 | 4.178125 | 3.97161 | 5.739907 | 6.324238 | 7.201776 |
| LOC_Os08g39150 | 4.002466 | 3.699773 | 3.998836 | 5.106009 | 3.963641 | 3.718338 | 0.801214 | 1.008988 | 0.764395 |
| LOC_Os08g38900 | 19.55665 | 30.24092 | 27.53225 | 34.45836 | 27.27108 | 23.03786 | 7.711309 | 6.547795 | 12.33769 |
| LOC_Os08g38820 | 2.985933 | 5.129029 | 3.905697 | 6.336017 | 6.79003 | 5.174629 | 1.264263 | 1.85583 | 1.802549 |
| LOC_Os08g37810 | 3.924337 | 3.382229 | 3.592963 | 3.456683 | 3.846388 | 4.20841 | 3.814107 | 3.835896 | 6.111127 |
| LOC_Os08g36450 | 4.795953 | 4.83389 | 4.888311 | 6.825104 | 5.330001 | 4.838272 | 6.047539 | 6.767163 | 9.10481 |
| LOC_Os08g36410 | 0.263569 | 0.363606 | 0.504994 | 0.292596 | 0.440228 | 0.401505 | 1.461767 | 0.949923 | 1.412209 |
| LOC_Os08g36250 | 0.133708 | 0.145935 | 0.118267 | 0.201445 | 0.145776 | 0.196242 | 0.436211 | 0.455246 | 0.741975 |
| LOC_Os08g36150 | 11.87882 | 11.53033 | 11.41802 | 13.32841 | 12.536 | 9.946806 | 2.156616 | 3.374524 | 3.887041 |
| LOC_Os08g35670 | 0.153969 | 0.199012 | 0.099532 | 0.555818 | 0.248201 | 0.391895 | 2.967729 | 0.802617 | 2.9908 |
| LOC_Os08g35620 | 6.61856 | 6.397384 | 5.050316 | 5.608793 | 5.205448 | 4.399581 | 8.513026 | 11.15334 | 10.1347 |
| LOC_Os08g35190 | 1.65618 | 1.612856 | 2.72054 | 5.699772 | 1.910599 | 2.261827 | 6.843402 | 5.409839 | 6.145866 |
| LOC_Os08g34580 | 3.179788 | 4.757504 | 3.94752 | 5.623826 | 4.991113 | 5.706295 | 10.14732 | 9.605432 | 13.56463 |
| LOC_Os08g34380 | 0.537565 | 0.688365 | 0.508767 | 0.623062 | 0.842824 | 0.704439 | 0.932303 | 1.204911 | 1.227356 |
| LOC_Os08g34280 | 27.73923 | 34.32522 | 28.03925 | 28.88564 | 30.09622 | 36.19952 | 4.882093 | 6.840232 | 7.137301 |
| LOC_Os08g34240 | 8.830229 | 12.05837 | 9.802228 | 13.66358 | 15.27291 | 11.92056 | 1.504765 | 3.259381 | 4.503218 |
| LOC_Os08g33940 | 1.718047 | 2.375881 | 2.420066 | 3.068058 | 3.027744 | 2.742584 | 3.941689 | 4.272214 | 5.681056 |
| LOC_Os08g33820 | 11.05494 | 23.69626 | 18.57207 | 34.27659 | 31.92935 | 20.91062 | 3.305223 | 3.199319 | 8.879398 |
| LOC_Os08g33640 | 3.058235 | 2.356621 | 1.978692 | 3.080556 | 2.293027 | 1.602964 | 9.814784 | 8.846416 | 7.81007 |
| LOC_Os08g33050 | 0.422823 | 0.524003 | 0.283201 | 0.591636 | 0.507076 | 0.44911 | 1.276878 | 0.873133 | 1.349838 |
| LOC_Os08g31690 | 0.808025 | 0.793379 | 0.767418 | 0.732519 | 0.617451 | 0.499274 | 1.73285 | 2.244088 | 1.356437 |
| LOC_Os08g31670 | 0.305161 | 0.37001 | 0.451774 | 0.467254 | 0.398177 | 0.541191 | 0.965272 | 0.730844 | 1.194734 |
| LOC_Os08g31630 | 2.559373 | 3.121933 | 3.974775 | 3.891515 | 4.507957 | 5.592047 | 8.686225 | 10.34105 | 16.5025 |
| LOC_Os08g31580 | 12.70104 | 9.208482 | 14.3161 | 21.01633 | 13.94582 | 13.3614 | 29.8326 | 29.45889 | 38.11311 |
| LOC_Os08g31510 | 1.422775 | 2.580545 | 2.630623 | 1.643918 | 2.846822 | 2.623354 | 3.529443 | 4.807593 | 7.344308 |
| LOC_Os08g31090 | 7.637712 | 5.695134 | 9.915824 | 11.35607 | 9.375072 | 8.214885 | 2.128201 | 2.338709 | 1.256588 |
| LOC_Os08g30719 | 0.983253 | 1.612669 | 1.339862 | 1.534216 | 2.489202 | 2.285055 | 0.156375 | 0.387808 | 0.656285 |
| LOC_Os08g29530 | 8.661979 | 8.493126 | 10.27001 | 18.11035 | 14.58272 | 16.6716 | 3.977147 | 3.767864 | 4.728998 |
| LOC_Os08g28710 | 0.73973 | 0.27476 | 0.800087 | 0.346755 | 0.085393 | 0.284369 | 0.95526 | 0.510968 | 1.897529 |
| LOC_Os08g28670 | 2.299266 | 1.201319 | 2.110266 | 2.887016 | 2.166918 | 2.23009 | 0.484791 | 0.489408 | 0.531958 |
| LOC_Os08g27840 | 1.859746 | 3.57406 | 2.672841 | 7.548458 | 4.747507 | 4.7478 | 4.642296 | 5.732882 | 9.162245 |
| LOC_Os08g27580 | 1.854078 | 2.571599 | 2.320817 | 4.529532 | 3.569618 | 4.018103 | 0.846522 | 0.985 | 1.63684 |
| LOC_Os08g23410 | 1.066848 | 0.64196 | 0.874256 | 1.513165 | 1.055007 | 1.335301 | 2.467903 | 3.626274 | 3.492718 |
| LOC_Os08g23120 | 4.889094 | 6.34325 | 7.164111 | 7.587453 | 7.67482 | 6.248973 | 1.030567 | 1.836516 | 3.224016 |
| LOC_Os08g19590 | 3.470291 | 3.546689 | 4.993596 | 8.14776 | 5.600667 | 8.95254 | 1.748202 | 1.927944 | 3.882301 |
| LOC_Os08g19210 | 6.141821 | 5.107252 | 5.257276 | 4.469058 | 5.080806 | 6.446167 | 5.945699 | 7.25659 | 8.394001 |
| LOC_Os08g17784 | 0.949458 | 1.886727 | 1.272442 | 1.382244 | 2.118307 | 1.667881 | 8.840372 | 8.571086 | 7.906805 |
| LOC_Os08g16810 | 0.489587 | 1.590193 | 1.259827 | 3.603416 | 3.83955 | 2.469844 | 0.397294 | 0.364017 | 0.907003 |
| LOC_Os08g13469 | 1.557526 | 1.995419 | 2.349107 | 3.405969 | 2.085771 | 2.342489 | 0.478425 | 0.62871 | 0.901794 |
| LOC_Os08g12430 | 2.035259 | 1.972026 | 1.682692 | 2.957988 | 2.418789 | 1.397487 | 0.909946 | 1.041238 | 0.866679 |
| LOC_Os08g10550 | 1.856307 | 1.577777 | 1.355382 | 2.426936 | 2.217239 | 1.536244 | 4.176635 | 3.681819 | 4.52259 |
| LOC_Os08g09390 | 0.670309 | 0.816761 | 1.110484 | 2.450913 | 1.818843 | 1.263945 | 0.304033 | 0.180797 | 0.406447 |
| LOC_Os08g08592 | 2.113918 | 2.149738 | 2.654621 | 3.95957 | 3.489269 | 4.244283 | 0.763478 | 0.913403 | 1.438671 |
| LOC_Os08g08120 | 1.695501 | 2.362416 | 2.723987 | 3.844245 | 3.094212 | 3.106404 | 3.735944 | 3.251868 | 5.124268 |
| LOC_Os08g08100 | 0.510506 | 1.265165 | 1.119572 | 1.282867 | 2.997169 | 1.28882 | 0.071012 | 0.098643 | 0.133069 |
| LOC_Os08g07330 | 0.069491 | 0.111759 | 0.192505 | 0.283591 | 0.212721 | 0.188974 | 0.20341 | 0.363756 | 0.404673 |
| LOC_Os08g06140 | 5.545632 | 6.414388 | 6.496831 | 7.332363 | 8.913453 | 9.341467 | 9.977664 | 9.778755 | 11.36506 |
| LOC_Os08g06130 | 1.004136 | 1.121881 | 1.175605 | 2.046145 | 1.51179 | 1.338224 | 0.369442 | 0.500457 | 0.562017 |
| LOC_Os08g06060 | 9.005189 | 7.325743 | 8.047813 | 6.857014 | 8.647725 | 10.56935 | 2.615875 | 2.989761 | 2.997653 |
| LOC_Os08g06010 | 17.17401 | 11.98376 | 14.35959 | 21.53543 | 14.38906 | 18.57094 | 69.87005 | 62.08796 | 79.67699 |
| LOC_Os08g05910 | 9.061569 | 11.19223 | 10.56168 | 12.5924 | 10.08153 | 11.73794 | 1.788189 | 2.900449 | 5.181136 |
| LOC_Os08g05720 | 3.040669 | 1.712552 | 3.353603 | 1.255531 | 1.9263 | 1.944943 | 3.444081 | 2.575732 | 5.602849 |
| LOC_Os08g04840 | 27.60061 | 20.77807 | 26.17321 | 18.20919 | 16.29256 | 17.73117 | 18.50263 | 21.5348 | 20.79272 |
| LOC_Os08g04810 | 2.405659 | 3.2712 | 3.12765 | 4.014071 | 3.581611 | 3.327357 | 0.990449 | 1.203113 | 1.379568 |
| LOC_Os08g04630 | 3.335619 | 2.686498 | 2.704605 | 2.393344 | 2.144822 | 1.644864 | 4.702815 | 6.032689 | 5.779735 |
| LOC_Os08g03990 | 4.559398 | 5.983345 | 5.570988 | 7.018771 | 8.104628 | 6.395042 | 1.567108 | 1.821589 | 2.170126 |
| LOC_Os08g03840 | 5.0401 | 6.133175 | 6.05242 | 7.82946 | 6.784931 | 5.626429 | 2.187309 | 2.642622 | 3.304678 |
| LOC_Os08g02140 | 5.878902 | 7.692693 | 6.489356 | 10.32698 | 9.860919 | 6.940749 | 2.617901 | 2.828462 | 4.062394 |
| LOC_Os07g49400 | 30.82267 | 47.87966 | 44.97793 | 70.72445 | 75.03733 | 67.11713 | 17.21797 | 17.06934 | 22.02188 |
| LOC_Os07g49230 | 1.074638 | 1.701926 | 1.291659 | 2.980338 | 2.911111 | 2.160173 | 0.342161 | 0.325749 | 0.322393 |
| LOC_Os07g48970 | 0.322811 | 0.725799 | 0.566604 | 1.901616 | 1.70695 | 1.456402 | 0.287289 | 0.236533 | 0.802232 |
| LOC_Os07g48820 | 4.650144 | 5.285501 | 5.385764 | 8.613753 | 6.944346 | 5.492101 | 2.436535 | 3.171513 | 3.854639 |
| LOC_Os07g48810 | 2.268341 | 2.971656 | 2.639792 | 3.056297 | 2.411481 | 2.231741 | 5.108497 | 5.155181 | 5.918321 |
| LOC_Os07g48630 | 2.520106 | 3.654419 | 2.633579 | 3.728862 | 3.463991 | 3.679853 | 5.15508 | 6.127905 | 8.274312 |
| LOC_Os07g48570 | 2.703346 | 2.059928 | 2.54267 | 3.174705 | 1.771265 | 2.43173 | 4.432571 | 4.322025 | 7.4853 |
| LOC_Os07g48550 | 5.562539 | 8.733934 | 8.461919 | 10.23314 | 9.254069 | 7.582981 | 11.0827 | 10.79043 | 18.32582 |
| LOC_Os07g48510 | 3.822217 | 1.96481 | 3.755207 | 4.141644 | 2.112241 | 2.900422 | 9.772126 | 4.364441 | 11.5173 |
| LOC_Os07g48450 | 0.284529 | 0.437685 | 0.636925 | 1.781369 | 0.538138 | 0.544095 | 3.839568 | 3.820819 | 4.139994 |
| LOC_Os07g48410 | 3.866341 | 5.915296 | 4.414842 | 9.644483 | 8.91338 | 8.730154 | 8.987249 | 9.082114 | 11.71129 |
| LOC_Os07g48370 | 0.992834 | 2.19414 | 1.700956 | 2.973546 | 3.401393 | 3.838096 | 0.969738 | 0.805056 | 2.192187 |
| LOC_Os07g48090 | 1.931296 | 1.599376 | 1.8595 | 2.684005 | 2.068938 | 2.106564 | 3.295074 | 3.600449 | 3.760561 |
| LOC_Os07g47670 | 40.49651 | 38.62495 | 44.9587 | 36.9278 | 33.37493 | 33.56993 | 67.47137 | 83.93876 | 95.56181 |
| LOC_Os07g47490 | 0.420499 | 0.801109 | 0.517192 | 1.754147 | 1.344165 | 1.196909 | 0.210491 | 0.284506 | 0.219804 |
| LOC_Os07g47350 | 4.545592 | 4.891112 | 4.522887 | 5.721764 | 4.696059 | 4.450501 | 7.177983 | 7.812158 | 8.313728 |
| LOC_Os07g47100 | 3.051625 | 2.365938 | 2.164224 | 2.146844 | 1.738366 | 1.634376 | 2.568936 | 4.559669 | 2.978253 |
| LOC_Os07g46670 | 7.682539 | 13.42047 | 11.86 | 16.13212 | 20.7479 | 15.94377 | 5.689164 | 5.400972 | 8.182777 |
| LOC_Os07g46630 | 0.809388 | 2.803099 | 2.000199 | 3.814563 | 4.577259 | 4.013779 | 0.668057 | 0.692435 | 1.033871 |
| LOC_Os07g44690 | 0.801518 | 0.756747 | 0.734486 | 0.71754 | 0.812695 | 0.835087 | 0.107865 | 0.126813 | 0.140587 |
| LOC_Os07g44410 | 6.961965 | 7.935268 | 8.456541 | 12.37799 | 11.45505 | 13.42929 | 26.57809 | 24.0012 | 30.46993 |
| LOC_Os07g44320 | 1.628423 | 1.682959 | 1.66383 | 2.328924 | 1.980204 | 1.977441 | 2.506929 | 2.613626 | 4.246029 |
| LOC_Os07g44190 | 5.210042 | 7.688736 | 5.682977 | 11.44346 | 9.428248 | 6.818836 | 1.853579 | 2.679662 | 3.71148 |
| LOC_Os07g44070 | 7.348142 | 9.781392 | 8.883315 | 9.857335 | 10.96003 | 10.51619 | 2.904287 | 3.467373 | 3.248414 |
| LOC_Os07g44060 | 64.3008 | 82.51673 | 76.32383 | 45.37153 | 79.03694 | 60.01841 | 30.48131 | 32.92341 | 31.60573 |
| LOC_Os07g43970 | 0.15851 | 0.221279 | 0.288368 | 0.353125 | 0.230849 | 0.276719 | 0.813929 | 0.742821 | 0.67563 |
| LOC_Os07g43950 | 15.86874 | 18.72415 | 17.50297 | 24.15527 | 23.41366 | 15.49447 | 5.294744 | 7.722476 | 6.252665 |
| LOC_Os07g43770 | 6.728166 | 5.965192 | 8.611365 | 4.518293 | 3.926133 | 4.179778 | 0.787158 | 0.932027 | 1.09135 |
| LOC_Os07g43510 | 3.79834 | 3.317237 | 3.633187 | 5.194608 | 4.001203 | 3.090027 | 1.054915 | 1.447959 | 1.583338 |
| LOC_Os07g43470 | 10.4821 | 13.19522 | 12.22847 | 18.49983 | 17.29505 | 13.56155 | 4.337005 | 4.456536 | 7.203535 |
| LOC_Os07g43360 | 5.749509 | 5.823041 | 5.322302 | 7.519241 | 7.152446 | 6.754932 | 8.64432 | 9.832095 | 11.46567 |
| LOC_Os07g42580 | 0.842332 | 0.344823 | 0.658368 | 0.506287 | 0.985972 | 1.001478 | 2.401008 | 2.107123 | 3.127866 |
| LOC_Os07g42380 | 2.374711 | 3.147589 | 2.957598 | 8.511317 | 5.874337 | 4.145947 | 1.73117 | 1.39664 | 1.747381 |
| LOC_Os07g42324 | 6.611514 | 0.997632 | 3.409575 | 1.423651 | 1.150415 | 2.306442 | 72.01819 | 62.44172 | 101.7205 |
| LOC_Os07g42220 | 7.833894 | 5.594937 | 11.44021 | 4.518969 | 5.596004 | 7.849698 | 8.718264 | 8.921018 | 12.74001 |
| LOC_Os07g41180 | 4.080132 | 3.582943 | 4.23766 | 8.962882 | 5.998661 | 5.457129 | 1.636799 | 1.168956 | 2.036822 |
| LOC_Os07g41160 | 1.107132 | 0.792884 | 1.224006 | 1.815441 | 0.944309 | 1.852964 | 4.414892 | 3.476247 | 5.401031 |
| LOC_Os07g40630 | 0.592237 | 1.09075 | 0.646507 | 0.837803 | 1.139981 | 0.836554 | 1.11154 | 2.036217 | 1.614837 |
| LOC_Os07g40000 | 1.129954 | 1.324858 | 2.139467 | 4.611836 | 2.302446 | 2.206703 | 3.982766 | 4.014986 | 7.446214 |
| LOC_Os07g39880 | 1.87831 | 1.423265 | 1.817233 | 2.918885 | 2.607623 | 2.137467 | 4.003616 | 2.734728 | 3.780475 |
| LOC_Os07g39780 | 4.574562 | 5.004 | 5.278931 | 5.778206 | 5.337778 | 4.892923 | 5.796679 | 7.111307 | 9.506534 |
| LOC_Os07g39680 | 88.02356 | 71.20561 | 109.8818 | 80.51096 | 85.07938 | 86.18296 | 96.77903 | 99.96719 | 118.7431 |
| LOC_Os07g39620 | 7.001597 | 10.03912 | 7.51216 | 11.07053 | 12.07556 | 10.6355 | 10.70754 | 12.57908 | 15.39493 |
| LOC_Os07g39520 | 1.637776 | 1.427801 | 2.214124 | 1.396486 | 1.467401 | 2.224273 | 2.881637 | 3.326201 | 4.772255 |
| LOC_Os07g39510 | 14.78157 | 20.16899 | 20.99774 | 20.33869 | 23.10265 | 23.35299 | 6.45379 | 7.830645 | 7.906501 |
| LOC_Os07g39430 | 1.839014 | 1.739182 | 1.781549 | 2.476791 | 1.665734 | 1.558268 | 2.621514 | 2.725654 | 2.994585 |
| LOC_Os07g39320 | 0.210683 | 0.136114 | 0.215915 | 1.906351 | 0.480343 | 0.5281 | 0.990779 | 0.870887 | 1.929579 |
| LOC_Os07g38530 | 12.01978 | 14.00397 | 13.24368 | 15.46265 | 15.7598 | 13.08988 | 16.89681 | 14.24736 | 18.74852 |
| LOC_Os07g38090 | 4.683407 | 5.813055 | 5.909349 | 5.725688 | 5.945471 | 6.463203 | 6.870803 | 7.528959 | 10.04517 |
| LOC_Os07g37890 | 12.39371 | 11.88732 | 13.76717 | 10.18751 | 10.41279 | 12.17733 | 13.99741 | 16.43991 | 18.02087 |
| LOC_Os07g37550 | 17.66417 | 34.56351 | 23.63508 | 44.29336 | 47.51554 | 40.72311 | 11.32338 | 15.58379 | 21.20204 |
| LOC_Os07g37320 | 1.553056 | 1.907714 | 2.845838 | 5.144509 | 2.449446 | 2.937155 | 18.65004 | 25.64197 | 35.58022 |
| LOC_Os07g37310 | 2.542099 | 2.87843 | 2.957798 | 2.132339 | 2.316251 | 3.132867 | 5.233968 | 6.062016 | 6.103842 |
| LOC_Os07g37180 | 3.590448 | 3.526019 | 3.980943 | 3.678827 | 2.906328 | 3.124474 | 10.08864 | 10.81689 | 13.89568 |
| LOC_Os07g37110 | 1.199212 | 1.880301 | 1.789545 | 3.961808 | 3.470353 | 2.805055 | 0.926738 | 0.583261 | 1.198977 |
| LOC_Os07g37030 | 21.31773 | 26.83417 | 26.21264 | 21.18456 | 23.05895 | 28.75794 | 21.31859 | 24.72844 | 35.61928 |
| LOC_Os07g36544 | 1.148538 | 1.657141 | 1.406548 | 1.609763 | 2.111966 | 1.360458 | 2.182729 | 3.314635 | 2.357631 |
| LOC_Os07g36490 | 3.987453 | 4.932864 | 4.7933 | 6.823412 | 5.861299 | 3.762389 | 1.54317 | 1.486772 | 1.795532 |
| LOC_Os07g36170 | 2.162091 | 1.924813 | 2.781521 | 4.005361 | 2.557357 | 2.923681 | 5.3103 | 4.520576 | 7.21696 |
| LOC_Os07g35335 | 0.266556 | 0.277295 | 0.331468 | 0.253523 | 0.324941 | 0.258772 | 0.620202 | 0.605112 | 0.974363 |
| LOC_Os07g34640 | 4.253193 | 3.872146 | 5.032297 | 4.180203 | 3.807698 | 4.430046 | 6.386686 | 5.307012 | 6.757747 |
| LOC_Os07g34570 | 4.574743 | 12.40849 | 8.531425 | 15.24243 | 27.21897 | 21.33195 | 34.78983 | 43.6294 | 32.65051 |
| LOC_Os07g33480 | 0.14474 | 0.640853 | 0.47098 | 1.687389 | 1.642593 | 1.333225 | 0.3243 | 0.186979 | 0.475122 |
| LOC_Os07g32350 | 3.065521 | 3.853748 | 3.464538 | 5.932024 | 4.778722 | 3.406551 | 1.576893 | 1.586775 | 2.053463 |
| LOC_Os07g31840 | 0.559283 | 0.624063 | 0.666281 | 0.799817 | 0.749992 | 0.759969 | 2.219218 | 1.840667 | 2.810453 |
| LOC_Os07g31830 | 0.392415 | 0.432803 | 0.539372 | 0.596889 | 0.595835 | 0.530851 | 1.663608 | 1.22128 | 1.850898 |
| LOC_Os07g31430 | 0.300575 | 0.411361 | 0.261713 | 0.659401 | 0.960298 | 0.757893 | 0.04354 | 0.090362 | 0.189404 |
| LOC_Os07g30930 | 0.372914 | 0.401057 | 0.396454 | 0.48296 | 0.481634 | 0.29278 | 1.059223 | 0.881392 | 1.140811 |
| LOC_Os07g28880 | 3.092369 | 2.462396 | 1.822445 | 1.999853 | 2.006784 | 1.623973 | 0.352282 | 0.628631 | 0.277105 |
| LOC_Os07g26630 | 28.47017 | 34.18375 | 31.35623 | 23.10595 | 28.13128 | 26.68854 | 1.997807 | 2.995992 | 2.432866 |
| LOC_Os07g26000 | 7.635296 | 5.988875 | 6.484327 | 8.818868 | 7.179256 | 8.198341 | 10.53099 | 9.420507 | 12.65218 |
| LOC_Os07g25960 | 0.99446 | 1.410264 | 1.474112 | 0.298139 | 0.805832 | 1.095277 | 1.095629 | 3.731671 | 1.322652 |
| LOC_Os07g22024 | 1.551419 | 1.966333 | 1.831237 | 2.614915 | 2.625486 | 1.905675 | 0.704843 | 0.77393 | 0.981763 |
| LOC_Os07g16030 | 0.826693 | 1.147399 | 1.155245 | 1.824744 | 1.629519 | 1.596858 | 0.278361 | 0.473292 | 0.688045 |
| LOC_Os07g14540 | 2.971559 | 3.910791 | 3.887774 | 7.27695 | 8.125104 | 8.735271 | 1.995055 | 1.76023 | 4.945989 |
| LOC_Os07g13600 | 2.552184 | 2.944141 | 3.244941 | 3.678177 | 3.387149 | 3.191063 | 1.056808 | 1.252051 | 1.708804 |
| LOC_Os07g12560 | 0.119799 | 0.147893 | 0.233242 | 0.497832 | 0.236571 | 0.305067 | 0.488921 | 0.415507 | 0.638279 |
| LOC_Os07g12340 | 1.96027 | 1.443338 | 1.454818 | 3.199352 | 2.856912 | 2.797477 | 8.998617 | 7.82666 | 10.75172 |
| LOC_Os07g11490 | 5.235065 | 5.384429 | 5.398873 | 6.91066 | 6.641756 | 5.421606 | 2.352678 | 2.406893 | 3.945327 |
| LOC_Os07g11070 | 1.048203 | 1.141157 | 1.119093 | 1.812637 | 1.441304 | 1.365784 | 2.866845 | 2.659527 | 4.451861 |
| LOC_Os07g10970 | 0.201711 | 0.236246 | 0.308084 | 0.748394 | 0.383217 | 0.384001 | 0.072322 | 0.048456 | 0.062156 |
| LOC_Os07g10920 | 1.22801 | 1.574497 | 1.377471 | 2.241859 | 1.918821 | 1.740012 | 0.522484 | 0.770515 | 0.714733 |
| LOC_Os07g09914 | 2.503904 | 3.786317 | 4.418868 | 4.023611 | 3.815652 | 4.312015 | 1.011611 | 1.053298 | 1.955982 |
| LOC_Os07g09000 | 2.412153 | 2.540642 | 2.445 | 3.891093 | 3.114276 | 3.242271 | 7.889145 | 7.144385 | 8.430835 |
| LOC_Os07g08710 | 73.37188 | 82.38254 | 59.19363 | 103.058 | 123.2527 | 106.4976 | 33.04932 | 48.08262 | 38.74675 |
| LOC_Os07g08460 | 0.644531 | 0.803091 | 0.884731 | 2.377978 | 1.172788 | 0.921281 | 4.516104 | 3.072436 | 4.336589 |
| LOC_Os07g08070 | 36.14512 | 30.12403 | 36.04217 | 22.07306 | 26.45572 | 36.28789 | 31.87009 | 39.08962 | 47.17701 |
| LOC_Os07g07974 | 4.899323 | 5.349152 | 4.526259 | 7.048726 | 6.380718 | 7.611472 | 7.268828 | 8.939298 | 10.62667 |
| LOC_Os07g07920 | 1.187663 | 0.911623 | 0.697261 | 0.485748 | 0.840631 | 0.298384 | 1.543576 | 1.494913 | 1.310667 |
| LOC_Os07g07030 | 2.50682 | 2.338991 | 2.857309 | 2.951625 | 2.484541 | 2.746551 | 3.816385 | 4.258095 | 5.330915 |
| LOC_Os07g05940 | 0.330135 | 0.486355 | 0.111016 | 0.266908 | 0.159407 | 0.130333 | 2.939973 | 5.025767 | 3.81757 |
| LOC_Os07g04690 | 11.70259 | 14.05669 | 14.94628 | 20.1759 | 17.45944 | 23.20059 | 17.76774 | 17.66145 | 27.61308 |
| LOC_Os07g02330 | 0.875625 | 0.846126 | 0.693291 | 1.076274 | 0.639716 | 0.959675 | 1.795848 | 2.155861 | 1.435082 |
| LOC_Os07g02200 | 2.993179 | 12.10156 | 6.348159 | 14.54919 | 21.98511 | 26.74337 | 2.277225 | 2.788358 | 9.368888 |
| LOC_Os07g01540 | 0.836144 | 0.560688 | 0.975165 | 0.740727 | 0.638281 | 0.394649 | 2.672138 | 2.648484 | 5.753787 |
| LOC_Os07g01030 | 3.336098 | 2.518617 | 2.736489 | 3.914964 | 2.951881 | 2.128036 | 6.522485 | 6.756499 | 7.384453 |
| LOC_Os06g51100 | 1.566972 | 1.670594 | 1.722789 | 2.564432 | 1.973202 | 1.93555 | 0.649435 | 0.756531 | 0.843237 |
| LOC_Os06g50220 | 1.823098 | 1.752237 | 2.376311 | 2.40701 | 1.815916 | 1.905888 | 3.58653 | 3.532044 | 4.051077 |
| LOC_Os06g49430 | 4.359172 | 5.112367 | 5.046457 | 6.879286 | 5.498432 | 5.997939 | 6.666782 | 7.624532 | 11.10609 |
| LOC_Os06g49220 | 0.239268 | 1.212098 | 0.651238 | 1.214302 | 1.404133 | 1.291382 | 0.218902 | 0.253594 | 0.348203 |
| LOC_Os06g49030 | 6.961565 | 6.231016 | 6.627776 | 10.71607 | 10.25559 | 6.200713 | 3.117196 | 2.897114 | 1.766646 |
| LOC_Os06g48350 | 47.01094 | 45.14623 | 49.18113 | 51.9047 | 42.93622 | 41.97674 | 8.893046 | 13.29316 | 18.01207 |
| LOC_Os06g48330 | 2.308599 | 3.120341 | 2.329945 | 3.659461 | 4.2916 | 2.968359 | 3.966318 | 3.960514 | 4.393559 |
| LOC_Os06g48200 | 5.829333 | 6.807317 | 8.776575 | 13.86028 | 7.496352 | 9.229245 | 11.92841 | 8.045212 | 17.30576 |
| LOC_Os06g47600 | 3.539188 | 4.194774 | 6.659066 | 7.206011 | 6.494361 | 13.27122 | 0.702862 | 0.570739 | 4.629517 |
| LOC_Os06g46900 | 34.75884 | 34.71055 | 32.69682 | 47.32238 | 51.67145 | 30.26106 | 9.132637 | 12.60353 | 9.690108 |
| LOC_Os06g46060 | 5.60492 | 4.271525 | 4.19436 | 7.72197 | 7.272867 | 6.57775 | 12.12577 | 12.7354 | 15.09673 |
| LOC_Os06g45840 | 10.38924 | 8.702259 | 9.945725 | 7.734815 | 7.196549 | 7.22457 | 11.78515 | 14.85947 | 14.43636 |
| LOC_Os06g45350 | 0.729577 | 1.075941 | 1.733454 | 1.991878 | 1.469575 | 2.191787 | 0.486589 | 0.434685 | 0.958967 |
| LOC_Os06g44620 | 10.41811 | 14.6983 | 12.91231 | 14.48792 | 15.09417 | 14.76578 | 16.11298 | 17.64685 | 20.98621 |
| LOC_Os06g44610 | 0.674619 | 4.066002 | 1.688273 | 4.035406 | 5.621007 | 3.47409 | 0.839814 | 0.899269 | 3.973828 |
| LOC_Os06g43870 | 12.95365 | 10.13839 | 13.10168 | 8.746796 | 7.986174 | 13.44356 | 24.88272 | 23.06439 | 30.97259 |
| LOC_Os06g43660 | 4.882783 | 5.952482 | 6.332289 | 8.391623 | 7.156582 | 6.93849 | 13.18887 | 12.02099 | 13.06111 |
| LOC_Os06g42810 | 11.00284 | 11.94909 | 11.29509 | 16.01084 | 14.71508 | 13.08652 | 14.89764 | 13.09008 | 17.15292 |
| LOC_Os06g42690 | 2.153509 | 2.718791 | 2.888541 | 2.103695 | 2.889775 | 1.903718 | 0.188523 | 0.473073 | 0.147283 |
| LOC_Os06g41880 | 0.491888 | 0.848991 | 1.155202 | 2.360694 | 1.302209 | 1.491284 | 0.354253 | 0.260989 | 0.437243 |
| LOC_Os06g41360 | 5.99809 | 5.240469 | 7.569103 | 4.605419 | 3.677661 | 4.239666 | 9.447626 | 11.78471 | 10.69451 |
| LOC_Os06g40730 | 2.645352 | 3.267868 | 3.196373 | 4.752413 | 4.03052 | 3.484491 | 5.475614 | 5.28148 | 6.186853 |
| LOC_Os06g40200 | 2.648455 | 2.560985 | 2.382014 | 3.263982 | 2.822863 | 2.592241 | 3.916107 | 3.689237 | 4.148636 |
| LOC_Os06g40120 | 6.488245 | 2.8445 | 3.813825 | 6.136481 | 4.75456 | 5.59631 | 49.2544 | 51.74467 | 57.70583 |
| LOC_Os06g40060 | 14.49142 | 14.03999 | 12.70049 | 9.61673 | 9.311193 | 10.17475 | 15.94458 | 18.22805 | 14.35025 |
| LOC_Os06g39750 | 3.547008 | 3.422178 | 3.800253 | 5.187584 | 4.385653 | 4.250512 | 0.797985 | 1.194179 | 1.714121 |
| LOC_Os06g38680 | 0.989787 | 0.918676 | 0.874875 | 1.100304 | 1.061132 | 1.004245 | 1.865147 | 1.490295 | 2.162939 |
| LOC_Os06g37660 | 10.43103 | 9.24669 | 13.05781 | 9.93926 | 10.47775 | 12.12653 | 13.06975 | 11.5756 | 18.01728 |
| LOC_Os06g36800 | 4.509854 | 4.498078 | 5.020381 | 5.903831 | 5.958927 | 5.248123 | 1.513179 | 2.334826 | 3.061229 |
| LOC_Os06g35960 | 36.69861 | 28.0654 | 16.96164 | 19.56182 | 22.38755 | 11.0691 | 46.25556 | 44.43045 | 35.64442 |
| LOC_Os06g34440 | 4.519963 | 5.250834 | 4.361278 | 6.573235 | 5.400762 | 4.534946 | 6.685602 | 7.230062 | 9.253942 |
| LOC_Os06g29430 | 1.724284 | 2.798614 | 2.143256 | 4.866347 | 5.101329 | 4.806642 | 1.240209 | 1.587896 | 3.426528 |
| LOC_Os06g28950 | 0.902197 | 1.418685 | 1.482519 | 1.933918 | 1.968549 | 1.403601 | 2.795112 | 2.375108 | 4.556789 |
| LOC_Os06g24870 | 3.004915 | 3.091962 | 2.849709 | 4.023889 | 3.278798 | 2.916083 | 4.212701 | 4.180707 | 4.877888 |
| LOC_Os06g23870 | 12.98389 | 14.19463 | 9.953883 | 28.23348 | 21.66543 | 15.77113 | 8.195221 | 8.904923 | 8.574265 |
| LOC_Os06g23530 | 3.263799 | 3.294691 | 2.736836 | 3.220768 | 2.981468 | 3.04699 | 5.177221 | 7.238889 | 10.25363 |
| LOC_Os06g23350 | 41.28734 | 26.36242 | 26.91164 | 19.27952 | 16.39222 | 13.82715 | 88.37065 | 108.3307 | 84.35203 |
| LOC_Os06g22960 | 20.20976 | 26.28257 | 25.22354 | 53.89035 | 28.30833 | 32.8725 | 45.50888 | 50.20685 | 53.0974 |
| LOC_Os06g21590 | 8.244385 | 17.57427 | 12.46031 | 38.43121 | 33.63379 | 25.31608 | 6.292012 | 4.876579 | 12.21158 |
| LOC_Os06g20310 | 0.442478 | 0.444872 | 0.3177 | 0.629361 | 0.397073 | 0.424357 | 0.873989 | 0.842772 | 0.741771 |
| LOC_Os06g19444 | 1.475816 | 1.637333 | 1.449315 | 1.921145 | 2.782406 | 2.104922 | 5.931574 | 4.851049 | 7.502761 |
| LOC_Os06g18850 | 0.094885 | 0.084986 | 0.111439 | 0.173228 | 0.140053 | 0.210776 | 0.249783 | 0.446351 | 0.583055 |
| LOC_Os06g16410 | 0.960256 | 0.967445 | 0.904475 | 1.227935 | 1.17317 | 0.916786 | 1.370272 | 1.284417 | 1.468356 |
| LOC_Os06g14240 | 5.537345 | 3.812314 | 2.608018 | 8.804573 | 6.898573 | 2.165904 | 0.10079 | 0.102505 | 0.156998 |
| LOC_Os06g13870 | 0.582249 | 0.440928 | 0.310105 | 0.398176 | 0.266964 | 0.281325 | 1.254275 | 1.214091 | 0.700172 |
| LOC_Os06g13680 | 200.9648 | 136.7585 | 159.069 | 224.3935 | 252.9199 | 201.9859 | 56.90527 | 50.5454 | 105.7987 |
| LOC_Os06g13560 | 0.641097 | 2.05643 | 1.09762 | 1.652305 | 1.402659 | 0.637902 | 0.184992 | 0.241542 | 0.324469 |
| LOC_Os06g12960 | 2.424064 | 2.642751 | 3.183324 | 3.291411 | 4.184907 | 3.883111 | 0.648339 | 0.768478 | 1.307366 |
| LOC_Os06g12530 | 15.64821 | 15.60022 | 14.77849 | 23.70443 | 23.35666 | 17.28504 | 6.514142 | 8.241945 | 8.49507 |

**Supplemental Table 7. Genetically specific DEGs.**

| Gene ID | ccT35 | ccDHX | ccJG809 | gzlT35 | gzlDHX | gzlJG809 | wcT35 | wcDHX | wcJG809 |
| --- | --- | --- | --- | --- | --- | --- | --- | --- | --- |
| LOC_Os12g44350 | 0.601832 | 0.704241 | 0.176872 | 1.366104 | 0.630415 | 0.165358 | 0.216381 | 0.313075 | 0.019947 |
| LOC_Os12g44040 | 1.068541 | 1.250359 | 0.279563 | 2.039323 | 2.17795 | 0.155088 | 0.958958 | 0.955764 | 0.156563 |
| LOC_Os12g43970 | 0.577069 | 0.765846 | 0.168436 | 0.926708 | 1.193979 | 0.278445 | 0.577599 | 0.620589 | 0.129119 |
| LOC_Os12g10200 | 3.313022 | 3.254727 | 1.630676 | 4.784333 | 4.30392 | 1.888774 | 2.717183 | 2.095879 | 1.434711 |
| LOC_Os12g09540 | 0.763536 | 0.82003 | 2.880983 | 0.505131 | 0.751642 | 2.061197 | 0.480371 | 0.5961 | 1.534305 |
| LOC_Os12g07160 | 1.059796 | 1.267599 | 4.561547 | 1.954964 | 1.732622 | 5.642104 | 0.715106 | 0.858747 | 5.986811 |
| LOC_Os12g07020 | 3.613642 | 3.667222 | 2.154723 | 5.397046 | 4.595221 | 2.355622 | 3.009732 | 3.09738 | 1.896714 |
| LOC_Os12g06550 | 0.45595 | 0.866382 | 0.10427 | 1.765949 | 1.898933 | 0.251587 | 0.713937 | 0.99141 | 0.037615 |
| LOC_Os12g04740 | 2.361617 | 2.63534 | 0.891774 | 4.135743 | 3.20113 | 0.838561 | 1.980056 | 1.517235 | 0.603642 |
| LOC_Os12g04610 | 1.890387 | 2.108211 | 0.446374 | 2.009868 | 1.518423 | 0.475411 | 1.749867 | 1.481377 | 0.637589 |
| LOC_Os12g04424 | 0.564599 | 0.767633 | 0.094314 | 0.518798 | 0.980344 | 0.208767 | 0.388562 | 0.598338 | 0.091599 |
| LOC_Os12g04370 | 1.211664 | 1.130353 | 0.487672 | 2.400302 | 2.163956 | 0.927394 | 0.681746 | 0.806774 | 0.284661 |
| LOC_Os12g01010 | 2.054395 | 2.923306 | 0.498854 | 3.735312 | 4.17492 | 0.519335 | 2.108943 | 1.726768 | 0.252944 |
| LOC_Os11g41130 | 6.692659 | 6.583917 | 10.85807 | 8.665593 | 8.327678 | 13.78374 | 4.64513 | 5.040333 | 11.78685 |
| LOC_Os11g11960 | 0.023648 | 0.113338 | 0.741854 | 0.194361 | 0.205522 | 0.858342 | 0.101254 | 0.150084 | 0.451389 |
| LOC_Os11g11790 | 0.574615 | 0.744257 | 0.277921 | 0.963148 | 1.374983 | 0.43481 | 0.475945 | 0.40415 | 0.236182 |
| LOC_Os10g38140 | 1.988063 | 2.298587 | 0.527059 | 1.187287 | 1.726696 | 0.247854 | 1.297046 | 1.617814 | 0.09674 |
| LOC_Os10g37880 | 3.634132 | 2.801601 | 0.570402 | 3.165539 | 3.949605 | 0.830728 | 2.662989 | 2.482553 | 0.23064 |
| LOC_Os10g37600 | 0.540518 | 0.632472 | 0.146755 | 0.892552 | 1.049181 | 0.125303 | 0.516481 | 0.539511 | 0.233362 |
| LOC_Os10g36950 | 6.280209 | 8.217551 | 2.095257 | 7.281242 | 10.93846 | 3.126412 | 2.816754 | 5.167221 | 1.865701 |
| LOC_Os10g36690 | 2.345043 | 2.581679 | 4.931379 | 6.231764 | 5.17486 | 12.99536 | 3.437587 | 3.333127 | 7.192746 |
| LOC_Os10g36340 | 10.06342 | 9.841283 | 2.3599 | 6.298176 | 8.853318 | 1.910754 | 6.216167 | 7.139485 | 1.806558 |
| LOC_Os10g29180 | 0.549923 | 0.686371 | 0.149799 | 0.776825 | 1.123843 | 0.186292 | 0.535613 | 0.624459 | 0.14599 |
| LOC_Os10g04890 | 4.040718 | 3.930939 | 0.58077 | 4.057011 | 6.602439 | 1.32318 | 5.988631 | 5.44805 | 2.030571 |
| LOC_Os09g29270 | 1.513297 | 1.51386 | 15.35248 | 0.610285 | 0.987368 | 19.11733 | 0.565449 | 0.430843 | 13.26266 |
| LOC_Os09g03710 | 0.015595 | 0.042974 | 0.142271 | 0.092014 | 0.047028 | 0.21771 | 0.006638 | 0.003199 | 0.130409 |
| LOC_Os08g44870 | 5.426789 | 6.561348 | 0.425228 | 5.32002 | 5.938419 | 0.584208 | 5.034379 | 4.985251 | 0.350987 |
| LOC_Os08g44015 | 2.542529 | 2.979873 | 1.352621 | 3.411475 | 3.137623 | 1.375098 | 1.359937 | 1.565263 | 0.894049 |
| LOC_Os08g43440 | 1.323826 | 1.591806 | 0.412845 | 1.994566 | 2.429226 | 0.915818 | 1.033163 | 1.239305 | 0.572097 |
| LOC_Os08g43040 | 4.550939 | 6.773912 | 0.270596 | 7.312176 | 6.544614 | 0.599493 | 2.661126 | 3.263396 | 0.059184 |
| LOC_Os08g42700 | 0.268585 | 0.331984 | 0.06431 | 0.488112 | 0.590695 | 0.045367 | 0.145818 | 0.171492 | 0.010183 |
| LOC_Os08g42320 | 1.368566 | 1.927415 | 0.054339 | 3.27984 | 2.653283 | 0.115075 | 1.429159 | 1.398048 | 0.065269 |
| LOC_Os08g42310 | 2.519343 | 2.078723 | 0.802486 | 3.035288 | 2.156332 | 0.633291 | 2.290335 | 1.734334 | 0.263391 |
| LOC_Os08g01660 | 5.39043 | 4.588063 | 10.97927 | 7.638862 | 8.6128 | 14.17848 | 4.380436 | 4.220663 | 13.58809 |
| LOC_Os08g01370 | 467.5276 | 256.6567 | 36.80821 | 356.2607 | 203.8361 | 26.66033 | 227.0247 | 204.9456 | 11.93012 |
| LOC_Os07g05680 | 1.679283 | 1.043952 | 10.02313 | 1.489963 | 1.295584 | 10.28499 | 0.891188 | 0.681851 | 9.797297 |
| LOC_Os07g05510 | 4.510835 | 5.1192 | 9.166182 | 6.882847 | 6.565761 | 11.92264 | 1.830089 | 2.646629 | 10.50781 |
| LOC_Os07g05400 | 9.727587 | 10.90613 | 6.302883 | 11.7955 | 11.12931 | 6.299487 | 5.227097 | 6.919616 | 3.956713 |
| LOC_Os06g48210 | 1.094459 | 1.623292 | 0.282943 | 0.847174 | 0.978768 | 0.113998 | 0.69927 | 1.498892 | 0.380275 |
| LOC_Os06g47130 | 1.118579 | 1.122571 | 4.301386 | 0.687287 | 0.795091 | 3.415149 | 0.337733 | 0.726934 | 1.589611 |
| LOC_Os06g46770 | 64.98661 | 95.09876 | 184.7871 | 121.307 | 133.1205 | 367.8702 | 77.19138 | 72.90041 | 226.4508 |
| LOC_Os06g45360 | 2.539471 | 2.577078 | 0.133228 | 3.804041 | 4.051646 | 0.327236 | 2.551843 | 2.228546 | 0.083421 |
| LOC_Os06g39660 | 0.472273 | 0.511153 | 2.843364 | 0.571338 | 0.793266 | 2.463871 | 0.537673 | 0.467925 | 2.438361 |
| LOC_Os06g39650 | 0.357895 | 0.441056 | 1.887039 | 0.509712 | 0.393342 | 1.64126 | 0.289879 | 0.227921 | 1.700661 |
| LOC_Os06g36650 | 0.174267 | 0.221374 | 0.058754 | 0.344885 | 0.273257 | 0.094078 | 0.134901 | 0.098429 | 0.013968 |
| LOC_Os06g36560 | 4.288578 | 5.749194 | 11.34157 | 10.38753 | 8.834431 | 13.81101 | 7.082183 | 8.99278 | 14.41142 |
| LOC_Os06g34790 | 18.29189 | 21.22424 | 6.277987 | 24.09778 | 28.26681 | 7.758409 | 13.81731 | 14.67888 | 4.929859 |
| LOC_Os06g33810 | 9.632225 | 9.845888 | 5.511211 | 13.30797 | 12.45271 | 6.263831 | 5.515935 | 5.489314 | 4.008178 |
| LOC_Os06g33720 | 0.161309 | 0.331129 | 2.629385 | 0.526843 | 0.429848 | 2.636965 | 0.080141 | 0.072936 | 0.76094 |
| LOC_Os06g33170 | 0.956996 | 1.73354 | 0.417845 | 2.449147 | 2.887461 | 0.834261 | 1.402954 | 1.394133 | 0.439778 |
| LOC_Os06g21820 | 0.650747 | 0.530657 | 4.624129 | 0.55134 | 0.804401 | 3.368762 | 0.335171 | 0.472575 | 3.487343 |
| LOC_Os12g44270 | 21.71982 | 13.73128 | 7.329903 | 24.60761 | 16.36618 | 6.385435 | 11.68408 | 7.112299 | 2.43268 |
| LOC_Os12g38100 | 9.098558 | 16.62091 | 31.53257 | 14.68036 | 14.04621 | 24.97325 | 9.119144 | 8.964178 | 30.51152 |
| LOC_Os12g38090 | 3.637712 | 6.90627 | 12.50061 | 5.86345 | 5.438384 | 10.14986 | 3.293757 | 2.999338 | 12.73035 |
| LOC_Os11g10180 | 0.08299 | 0.775973 | 0.34894 | 0.277722 | 1.06292 | 0.39418 | 0.075417 | 0.603001 | 0.263478 |
| LOC_Os07g20720 | 0.308785 | 0.660107 | 0.101037 | 0.548953 | 0.939112 | 0.121438 | 0.621371 | 0.473539 | 0.111186 |
| LOC_Os07g09675 | 2.401341 | 1.45795 | 0.157439 | 1.647727 | 1.541643 | 0.195196 | 1.543394 | 1.335901 | 0.06228 |
| LOC_Os11g31060 | 27.82081 | 9.876376 | 12.31127 | 24.77968 | 11.33362 | 4.92434 | 6.170154 | 14.57699 | 7.45101 |
| LOC_Os11g28170 | 0.186555 | 0.853381 | 1.424424 | 0.109884 | 0.83182 | 0.907057 | 0.228906 | 1.294193 | 1.185013 |
| LOC_Os11g12810 | 2.575513 | 4.412192 | 5.022799 | 2.234039 | 2.987711 | 5.579437 | 1.781076 | 2.206993 | 4.967408 |
| LOC_Os09g27744 | 1.012504 | 8.191647 | 10.26692 | 2.022875 | 10.37363 | 10.22491 | 0.592986 | 4.405308 | 5.124995 |
| LOC_Os09g27734 | 0.338591 | 1.443823 | 1.979277 | 0.2361 | 1.795281 | 1.747211 | 0.197913 | 1.727143 | 2.715466 |
| LOC_Os08g25250 | 0.524454 | 1.443808 | 1.202517 | 0.386635 | 2.088712 | 1.321075 | 0.022298 | 0.899096 | 0.769555 |
| LOC_Os08g15840 | 10.18222 | 30.96214 | 29.32439 | 8.886384 | 22.7611 | 18.58855 | 7.367744 | 18.98865 | 13.6384 |
| LOC_Os07g47840 | 22.37714 | 7.641664 | 7.616673 | 13.33927 | 7.177978 | 3.242009 | 15.3871 | 16.19477 | 8.92297 |
| LOC_Os07g40860 | 6.53287 | 3.177505 | 4.037577 | 5.326519 | 1.414832 | 3.985916 | 4.562057 | 2.00928 | 2.088473 |
| LOC_Os07g40620 | 5.502157 | 1.23183 | 1.544291 | 7.466232 | 2.012941 | 1.604025 | 4.645636 | 1.147265 | 1.558283 |
| LOC_Os07g40580 | 70.77849 | 47.2366 | 55.39452 | 107.8243 | 54.09366 | 60.437 | 71.95664 | 37.00172 | 51.09615 |
| LOC_Os07g06500 | 6.195364 | 0.816488 | 0.799918 | 7.273124 | 0.91834 | 0.749026 | 4.877017 | 0.672958 | 0.630528 |
| LOC_Os12g39970 | 3.401293 | 27.62042 | 2.923636 | 3.837539 | 34.60109 | 2.821742 | 3.232434 | 25.42005 | 3.650079 |
| LOC_Os11g47320 | 0.598622 | 0.200253 | 0.874373 | 1.4272 | 0.192757 | 1.311175 | 0.735089 | 0.059525 | 0.888078 |
| LOC_Os11g47130 | 0.451077 | 0.069625 | 0.48225 | 0.653698 | 0.133646 | 0.69867 | 0.402151 | 0.115177 | 0.530014 |
| LOC_Os11g47120 | 2.957853 | 0.92758 | 2.992444 | 3.622948 | 1.2077 | 3.63162 | 2.541284 | 0.606698 | 3.440515 |
| LOC_Os11g45750 | 0.476533 | 0.059153 | 0.665857 | 1.032515 | 0.086898 | 0.766838 | 0.268532 | 0.048148 | 0.385268 |
| LOC_Os11g40150 | 0.526489 | 1.393682 | 0.553938 | 0.411338 | 1.026913 | 0.251935 | 0.335525 | 2.401519 | 0.514587 |
| LOC_Os11g40140 | 18.33934 | 5.444028 | 22.02676 | 24.94496 | 6.056263 | 26.98793 | 16.91477 | 2.822514 | 24.58743 |
| LOC_Os11g40120 | 1.049143 | 0.368251 | 1.358504 | 1.521439 | 0.474574 | 1.352703 | 0.487979 | 0.062383 | 0.747641 |
| LOC_Os11g37980 | 0.81472 | 2.589401 | 1.046397 | 1.361706 | 2.904584 | 1.023784 | 0.912621 | 2.95601 | 0.991299 |
| LOC_Os11g09990 | 0.855741 | 0.383069 | 1.062803 | 1.853763 | 0.500064 | 1.41275 | 0.709063 | 0.151509 | 0.824959 |
| LOC_Os11g08569 | 1.972112 | 0.379745 | 2.117225 | 3.253209 | 0.740014 | 2.553287 | 1.48293 | 0.263843 | 2.267429 |
| LOC_Os11g01180 | 4.718424 | 0.747557 | 3.420513 | 3.880912 | 0.916182 | 4.12655 | 2.456681 | 1.017765 | 3.903617 |
| LOC_Os11g01170 | 13.20651 | 7.87032 | 14.05217 | 20.01769 | 9.716115 | 15.36336 | 10.88804 | 5.771106 | 13.37297 |
| LOC_Os10g33830 | 3.311098 | 19.96728 | 2.02685 | 2.476563 | 35.19202 | 2.132752 | 0.996895 | 5.176634 | 1.214754 |
| LOC_Os10g33080 | 0.341994 | 0.094517 | 0.521118 | 0.891113 | 0.109033 | 0.884327 | 0.226587 | 0.022647 | 0.263646 |
| LOC_Os10g32690 | 14.73122 | 4.647027 | 16.54192 | 12.59068 | 4.292024 | 12.58734 | 9.318615 | 4.762163 | 15.19026 |
| LOC_Os10g28009 | 1.325604 | 2.630745 | 1.235348 | 1.325868 | 2.572456 | 1.492597 | 0.680187 | 2.483799 | 1.044263 |
| LOC_Os10g25810 | 3.603997 | 0.329793 | 2.603505 | 1.541583 | 0.477074 | 2.22331 | 1.710741 | 0.138658 | 1.710032 |
| LOC_Os10g22560 | 1.26093 | 0.243269 | 1.337216 | 1.508699 | 0.351983 | 1.544829 | 0.507797 | 0.121741 | 0.826117 |
| LOC_Os10g22484 | 0.831285 | 0.090524 | 0.656975 | 1.549852 | 0.149033 | 1.134496 | 0.541525 | 0.066354 | 0.794539 |
| LOC_Os10g22330 | 10.46236 | 4.538543 | 13.34113 | 16.14449 | 8.598111 | 19.02459 | 8.800861 | 3.286261 | 14.50796 |
| LOC_Os10g22039 | 17.01336 | 10.62921 | 17.3226 | 33.4228 | 11.43813 | 18.22875 | 16.86673 | 8.76338 | 22.21131 |
| LOC_Os10g17489 | 1.716762 | 0.152254 | 1.701867 | 2.348226 | 0.209204 | 1.606597 | 1.148118 | 0.095304 | 1.51124 |
| LOC_Os10g11340 | 0.345502 | 1.915452 | 0.565364 | 0.524973 | 1.945164 | 0.708319 | 0.045052 | 0.634535 | 0.440307 |
| LOC_Os10g08018 | 0.993376 | 0.365809 | 1.102189 | 1.653167 | 0.284303 | 1.123847 | 1.233231 | 0.526058 | 1.246492 |
| LOC_Os10g05680 | 1.261673 | 3.592579 | 1.212146 | 1.761768 | 3.891616 | 1.134819 | 0.484862 | 3.288667 | 0.774215 |
| LOC_Os09g17870 | 0.715191 | 0.079152 | 0.963294 | 1.585917 | 0.167848 | 1.188238 | 0.594436 | 0.047139 | 0.796041 |
| LOC_Os08g41780 | 1.24996 | 0.35536 | 1.203499 | 0.663987 | 0.164967 | 0.666106 | 1.184778 | 0.347098 | 1.055304 |
| LOC_Os08g34740 | 2.508819 | 1.195517 | 2.211334 | 3.30055 | 1.256635 | 1.623017 | 1.325299 | 0.682333 | 1.597897 |
| LOC_Os08g32870 | 12.75133 | 2.936798 | 13.70986 | 15.86898 | 3.608532 | 15.60478 | 12.13589 | 2.269214 | 18.4345 |
| LOC_Os08g30150 | 1.807356 | 0.73599 | 1.997461 | 2.980437 | 0.80798 | 3.268907 | 1.572863 | 0.515186 | 2.29237 |
| LOC_Os08g29200 | 0.353685 | 3.759717 | 0.50425 | 1.293732 | 5.014805 | 1.059006 | 0.214497 | 1.98977 | 0.422932 |
| LOC_Os08g29100 | 1.160069 | 0.23411 | 1.381861 | 1.352965 | 0.35272 | 0.983675 | 0.918249 | 0.202955 | 1.405238 |
| LOC_Os08g23754 | 1.233046 | 0.12788 | 0.842493 | 1.984631 | 0.231647 | 1.455313 | 0.40713 | 0.035198 | 0.447321 |
| LOC_Os08g23710 | 41.21748 | 28.3721 | 43.79935 | 59.09873 | 35.75364 | 44.2913 | 32.21836 | 20.85313 | 35.9888 |
| LOC_Os08g17370 | 3.620197 | 1.90248 | 4.856832 | 6.024368 | 2.415562 | 6.072426 | 1.889406 | 1.108988 | 3.515348 |
| LOC_Os08g16130 | 1.842193 | 0.629277 | 2.583027 | 4.00575 | 0.74088 | 3.348482 | 1.352097 | 0.321674 | 2.171318 |
| LOC_Os08g13060 | 1.506597 | 0.142406 | 1.417609 | 2.120352 | 0.206602 | 1.785575 | 1.148273 | 0.074627 | 1.358256 |
| LOC_Os07g38280 | 0.782354 | 0.317525 | 0.741958 | 1.591304 | 0.451152 | 1.000595 | 0.870886 | 0.268681 | 1.205087 |
| LOC_Os07g36150 | 0.686809 | 0.187864 | 0.612516 | 0.981408 | 0.250574 | 0.789468 | 0.462696 | 0.112596 | 0.681427 |
| LOC_Os07g34620 | 41.77775 | 20.31541 | 65.88784 | 58.59249 | 23.45954 | 78.87094 | 35.7255 | 6.746386 | 72.63446 |
| LOC_Os07g34250 | 0.411305 | 1.028504 | 0.279762 | 0.510928 | 1.513753 | 0.281341 | 0.096021 | 0.473336 | 0.284453 |
| LOC_Os07g34006 | 2.670379 | 6.859981 | 2.443023 | 3.244776 | 6.43676 | 2.735235 | 1.534963 | 3.471191 | 2.255196 |
| LOC_Os07g33954 | 2.881696 | 7.686295 | 2.706497 | 3.478577 | 7.202679 | 3.004718 | 1.66716 | 3.972679 | 2.500304 |
| LOC_Os07g07820 | 1.764305 | 0.155953 | 1.399274 | 2.488192 | 0.169604 | 1.547767 | 1.067904 | 0.091418 | 1.265429 |
| LOC_Os07g03180 | 14.88941 | 3.781576 | 18.62171 | 12.09354 | 3.114377 | 15.82106 | 11.89944 | 1.533128 | 19.60945 |
| LOC_Os06g49840 | 1.264162 | 2.881556 | 0.806309 | 0.597318 | 2.026152 | 0.638977 | 0.464862 | 1.445869 | 0.515976 |
| LOC_Os06g42000 | 23.09776 | 10.83657 | 20.25031 | 26.34072 | 13.712 | 22.14555 | 20.58667 | 10.70536 | 19.03968 |
| LOC_Os06g38320 | 1.854095 | 0.447056 | 2.27184 | 2.470767 | 0.384584 | 1.481727 | 0.647329 | 0.138647 | 1.215385 |
| LOC_Os06g16050 | 2.899374 | 0.218785 | 2.729661 | 3.945159 | 0.22863 | 2.572351 | 1.787064 | 0.148297 | 2.563723 |

**Supplemental Table 8. qRT-PCR analysis of DEGs affected by genotype × environment interactions.**

| Gene ID | ccT35 | ccDHX | ccJG809 | gzlT35 | gzlDHX | gzlJG809 | wcT35 | wcDHX | wcJG809 | R square |
| --- | --- | --- | --- | --- | --- | --- | --- | --- | --- | --- |
| LOC_Os11g18570 | 0.0083 | 0.0284 | 0.0850 | 0.9149 | 0.5662 | 1.5361 | 0.3754 | 0.1058 | 0.3596 | 0.6582 |
| LOC_Os11g09020 | 0.3022 | 0.7017 | 2.3998 | 2.1388 | 1.9377 | 7.0973 | 0.6226 | 0.5149 | 9.1088 | 0.4981 |
| LOC_Os10g01060 | 0.1313 | 0.1285 | 0.0313 | 0.1666 | 0.1609 | 0.1793 | 0.2639 | 0.4631 | 0.2668 | 0.8304 |
| LOC_Os09g25150 | 0.3447 | 0.1560 | 1.9927 | 0.0984 | 0.0913 | 0.0564 | 0.0507 | 0.0750 | 0.0398 | 0.7477 |
| LOC_Os07g47550 | 0.0010 | 0.0008 | 0.0003 | 0.0001 | 0.0001 | 0.0000 | 0.0006 | 0.0008 | 0.0003 | 0.5445 |
| LOC_Os12g08760 | 0.0119 | 0.1189 | 0.0013 | 0.0370 | 0.0148 | 0.0215 | 0.0299 | 0.0193 | 0.0143 | 0.5568 |
| LOC_Os08g43334 | 0.0137 | 0.0042 | 0.0016 | 0.0043 | 0.0043 | 0.0028 | 0.0006 | 0.0001 | 0.0008 | 0.8393 |
| LOC_Os12g36880 | 0.3925 | 2.4981 | 0.0619 | 3.4570 | 6.4584 | 2.6562 | 0.1137 | 0.3984 | 1.9441 | 0.9001 |
| LOC_Os10g41550 | 0.0005 | 0.0002 | 0.0009 | 0.0009 | 0.0004 | 0.0004 | 0.0020 | 0.0022 | 0.0501 | 0.7330 |
| LOC_Os09g23540 | 0.0002 | 0.0085 | 0.0010 | 0.0207 | 0.0570 | 0.0030 | 0.0018 | 0.0045 | 0.0139 | 0.8967 |
| LOC_Os07g38070 | 0.0038 | 0.0065 | 0.0204 | 0.0148 | 0.0048 | 0.0132 | 0.3831 | 0.0095 | 0.1073 | 0.8351 |

R square was calculated between the qRT-PCR data and RNA-seq data.

**Supplemental Table 9. Primers used in the qRT-PCR analysis.**

| **Gene ID** |  | **5’-3’** |
| --- | --- | --- |
| LOC_Os11g18570 | F | CGAGGTTTTGGTCGCTCAAG |
|  | R | AACTTGCACACACACACACA |
| LOC_Os11g09020 | F | ACGCGCTCAAGGTGTACC |
|  | R | CTGCCCATACGACGTACACA |
| LOC_Os10g01060 | F | CGCCCTTCGTTTTGCCATC |
|  | R | ACAGTGACATTTGCTCGATACA |
| LOC_Os09g25150 | F | GCACCTTCCAGTTCTGAGCA |
|  | R | TGAAGGGGCAGAATTCACGT |
| LOC_Os07g47550 | F | TCGGTGATGGAGTCGATGTC |
|  | R | GAACTCGCCGAACCGCTC |
| LOC_Os12g08760 | F | GCTTCTCCGTCAACTCCTCC |
|  | R | TGACAGCCACATTTCGATTGA |
| LOC_Os08g43334 | F | GAGGGAATGATGAGCGCGAA |
|  | R | GGATGGGCTGGTAAACGGG |
| LOC_Os12g36880 | F | TCGTGGATGGCTACTATGGC |
|  | R | TTGGACATTTCTGCGGCTCT |
| LOC_Os10g41550 | F | CGTACCTGCGCATGAACAAG |
|  | R | ACGTAGAGGTCCGACTGCT |
| LOC_Os09g23540 | F | CCAGGCCATGCTCGACTTC |
|  | R | CGGACGTCGTTCTTCTCCAG |
| LOC_Os07g38070 | F | TGAGGTCCATCGAAAGCACG |
|  | R | ATGCCCTTTCGTAGCGTCAA |
